# A 3q gene signature associated with triple negative breast cancer organ specific metastasis and response to neoadjuvant chemotherapy

**DOI:** 10.1038/srep45828

**Published:** 2017-04-07

**Authors:** Jun Qian, Heidi Chen, Xiangming Ji, Rosana Eisenberg, A. Bapsi Chakravarthy, Ingrid A. Mayer, Pierre P. Massion

**Affiliations:** 1 Division of Pulmonary and Critical Care Medicine, Department of Medicine, Vanderbilt University Medical Center, Nashville, TN, USA; 2 Vanderbilt Center for Quantitative Sciences, Department of Statistics, Vanderbilt University Medical Center, Nashville, TN, USA; 3 Department of Pathology, Microbiology and Immunology, Vanderbilt University Medical Center, Nashville, TN, USA; 4 Department of Radiation Oncology, Vanderbilt University Medical Center, Nashville, TN, USA; 5 Divsion of Oncology, Department of Medicine, Vanderbilt-Ingram Cancer Center, Vanderbilt University Medical Center, Nashville, TN, USA; 6 Veterans Affairs Medical Center, Nashville, TN, USA

## Abstract

Triple negative breast cancers (TNBC) are aggressive tumors, with high rates of metastatic spread and targeted therapies are critically needed. We aimed to assess the prognostic and predictive value of a 3q 19-gene signature identified previously from lung cancer in a collection of 4,801 breast tumor gene expression data. The 3q gene signature had a strong association with features of aggressiveness such as high grade, hormone receptor negativity, presence of a basal-like or TNBC phenotype and reduced distant metastasis free survival. The 3q gene signature was strongly associated with lung metastasis only in TNBC (P < 0.0001, Hazard ratio (HR) 1.44, 95% confidence interval (CI), 1.31–1.60), significantly associated with brain but not bone metastasis regardless of TNBC status. The association of one 3q driver gene FXR1 with distant metastasis in TNBC (P = 0.01) was further validated by immunohistochemistry. In addition, the 3q gene signature was associated with better response to neoadjuvant chemotherapy in TNBC (P < 0.0001) but not in non-TNBC patients. Our study suggests that the 3q gene signature is a novel prognostic marker for lung and/or brain metastasis and a predictive marker for the response to neoadjuvant chemotherapy in TNBC, implying a potential role for 3q genes in the mechanism of organ-specific metastasis.

Breast cancer is the most frequent malignant disease in women worldwide. Patients with breast cancer are at risk of experiencing metastasis for their lifetime. It is not the primary tumor, but its metastases at distant sites such as lung, bone and liver that are the main cause of death in these patients^1^. Many gene expression studies have demonstrated that breast cancer is a clinically and molecularly heterogeneous disease comprising subtypes with distinct gene expression patterns and outcomes, making it difficult not only to cure this disease, but also to assess risk factors for metastasis^2^. A small number of expression profiling strategies have been successfully developed and validated for clinical use, some of which are now commercially available^2^. Nevertheless, uncertainty remains in the clinical use of many breast gene signatures. Moreover, new prognostic markers are urgently needed to identify patients who are at the highest risk for developing metastases in each subtype of breast cancer, which might enable oncologists to begin tailoring treatment strategies^3^.

Amplification of the chromosomal region 3q26-29 is the most frequent genomic alteration in primary squamous cell lung cancers and occurs in many other cancers including breast cancer^4^,^5^. Recent comprehensive genomic studies in breast cancer reveal that gene copy number (CN) changes correlated with mRNA subtype including characteristic loss of 5q and gain of 3q, 10p in basal-like cancers and gain of 1q and 16q loss in luminal tumors^5^. Earlier studies showed that gains of chromosome 3q, 9p, 11p and 11q and loss of 17p are associated with breast cancer recurrence^6^. In an effort to identify oncogenic drivers in lung cancer associated the 3q26-29 amplicon, we previously integrated genomic and gene expression analysis of 593 primary lung squamous carcinoma from seven independent datasets and identified 20 driver genes in this amplicon^7^. Some of these driver genes such as phosphatidylinositol-4,5-bisphosphate 3-kinase catalytic subunit alpha (PIK3CA), fragile X mental retardation, autosomal homolog 1 (FXR1) and protein kinase C iota (PRKCI) have been implicated in the progression of lung or breast cancers^8^,^9^,^10^.

In this report, we interrogated the expression profiles of 4,801 breast tumors and report that this 3q gene expression signature is associated with poor outcomes in node negative breast cancer patients. We discovered that the 3q gene signature is strongly associated with the risk of developing lung and/or brain specific metastasis and the response to neoadjuvant chemotherapy in triple negative breast cancer (TNBC).

## Results

### 3q-gene signature is associated with aggressive behavior of breast cancer

**Figure 1:**
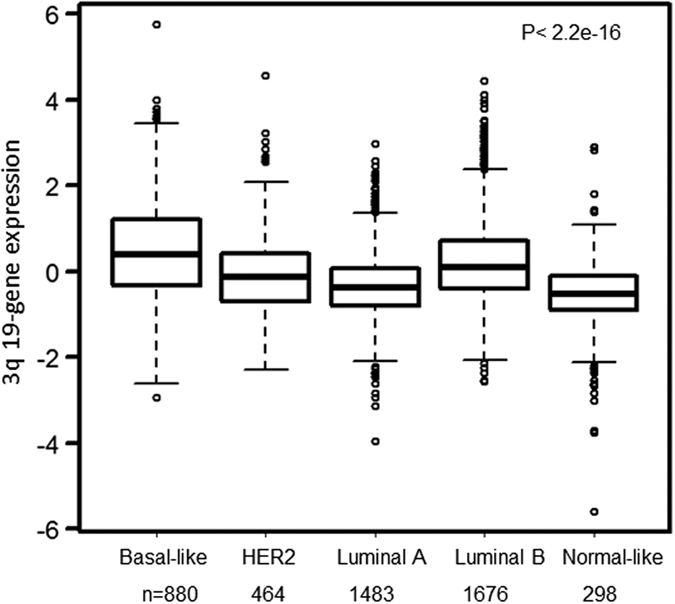
The 3q 19-gene signature is associated with Basal-like and Luminal B subtypes of breast cancer. PAM50 subtypes of 4801 tumors were calculated using genefu R package. P value was calculated using Kruskal–Wallis analysis of variance analysis (ANOVA).

Among the 4,801 patients with breast cancer, we tested the association between the 3q 19-gene signature and established prognostic variables including age, grade, tumor size, lymph node status, and the expression status of ER, PR and HER2. The 3q gene signature was significantly associated with higher grade (P < 2.2e-16), larger tumor size (P = 0.005), ER- (P = 1.42e-08) and PR- status (P = 4.75e-10), but not associated with age (P = 0.07), HER2 status (P = 0.53) or lymph node involvement (P = 0.26) (Table 1 and Supplemental Fig. 1). The 3q gene signature was significantly associated with basal-like and luminal B subtypes of breast tumors (P < 2.2e-16, Fig. 1) or TNBC (P = 3.06e-12, Supplemental Fig. 1). Moreover, both univariable Cox analysis and a meta-analysis indicated that the high 3q gene signature was significantly associated with worse distant metastasis–free survival (DMFS) (P = 3.25e-05), but not recurrence-free survival (RFS) (P = 0.07), OS (P = 0.29) or DSS (P = 0.24) (Table 1 and Supplemental Figs 2 and 3).

### 3q gene signature is associated with distant metastasis in node-negative breast cancer

**Figure 2:**
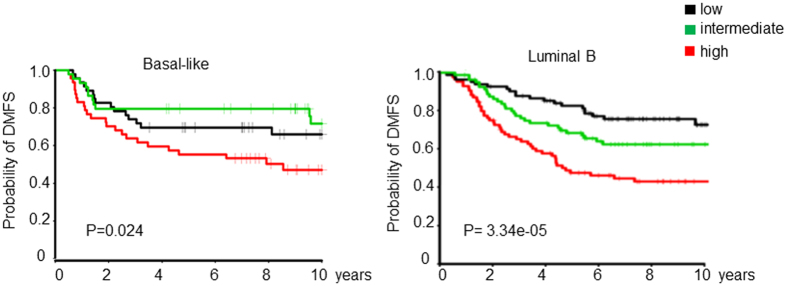
KM plot showed that high 3q gene signature was significantly associated with worse distant metastasis free survive (DMFS) in basal-like (n = 137) and luminal B (n = 254) subtypes of node-negative breast cancer patients (n = 788). The log-rank P values are shown.

To identify determinants of recurrence independent of treatment, we focused on a subset of lymph node-negative breast tumor samples in three datasets (GSE11121, GSE2034 and TRANSBIG) obtained from 788 patients who did not receive systemic neoadjuvant or adjuvant treatment and had DMFS information available (Supplemental Table 2). The expression of the 3q gene signature was not different across three datasets (P = 0.99, Supplemental Figure 4) but was significantly associated with DMFS in all three cohorts (Supplemental Table 3). After adjustment for age, ER, PR and HER2 status, grade and/or tumor size, multivariable Cox analysis demonstrated that the 3q gene signature remained an independent prognostic marker for DMFS in GSE11121 (HR 1.83, 95% CI 1.41–2.37, P = 6.45e-06) and GSE2034 (HR 1.38, 95% CI 1.11–1.72, P = 0.004, Supplemental Table 4), respectively. In the TRANSBIG dataset, the 3q gene signature tended to be an independent prognostic marker for DMFS (HR 1.18, 95% CI 0.95–1.47, P = 0.13, Supplemental Table 4). In the combined two datasets (GSE11121 + TRANSBIG, P = 0.03) or three datasets (GSE2034 + GSE11121 + TRANSBIG, P = 0.0001), the association remained significant independent of variables available (Supplemental Table 4). The association also remained statistically significant when adjusted for PAM50 molecular subtypes (HR 1.38, 95% CI 1.23–1.55, P = 8.43e-08, Supplemental Table 5) or five well-established gene signatures such as the 70-gene, 76-gene, Genomic Grade Index (GGI), Oncotype Dx and proliferating cell nuclear antigen (PCNA)-117 gene signatures in pooled datasets (HR 1.24, 95% CI 1.14–1.36, P = 7.8e-07, Supplemental Table 6). Schmidt *et al*. reported a B-cell metagene as a prognostic factor for highly proliferating tumors, independent of the proliferation, ER, and T-cell metagenes in the same combined cohort^11^. Our study showed that the 3q gene signature was also significantly associated with worse DMFS independently of B-cell metagene (HR 1.26, 95% CI 1.17–1.36, P = 2.96e-09, Supplemental Table 7). Further analysis showed that the 3q gene signature was able to identify high risk patients in fast-proliferating tumors such as Basal-like and Luminal B subtypes of breast cancer (Fig. 2 and Supplemental Table 8), but not in HER2 or Normal-like subtypes after adjusting for B-cell and proliferation metagene signatures (Supplemental Table 9). Therefore, we concluded that high expression of the 3q genes is an independent prognostic factor for DMFS in Basal-like and Luminal B subtypes of node-negative breast cancer patients and has added prognostic value to known prognostic variables and known proliferation or immune response related gene signatures.

### 3q gene signature is an independent prognostic marker for triple negative breast cancer lung and brain metastasis

The ability to predict the risk and location of tumor recurrence could influence the surveillance for patients with a history of breast cancer. Therefore, we sought to ask whether the 3q gene signature (originally derived from lung cancer gene expression analysis) is associated with breast cancer metastasis to specific organ sites such as the lung. To test this hypothesis, we analyzed expression profiles of three independent cohorts of patients with breast cancer where the outcome information on lung, brain and bone specific metastasis was available (EMC344, GSE12276 and GSE2603, Supplemental Table 10). The univariable Cox analysis showed that the 3q gene signature was significantly associated with shorter lung metastasis–free survival in all three dataset (P = 0.0008, 8.58e-06, and 0.0032 for EMC344, GSE12276, and GSE2603, respectively), whereas only associated with shorter brain metastasis–free survival in two datasets (P = 0.00003 in EMC344 and P = 0.008 in GSE12276), and associated with shorter bone metastasis–free survival only in EMC344 (P = 0.013, Supplemental Table 11). When tested on the combined cohort (n = 618), the 3q gene signature was highly correlated to lung (P < 2.0e-16, HR 1.74, 95% CI 1.60 to 1.9) and brain metastasis (P = 4.79e-11, HR 1.99, 95% CI 1.62 to 2.45) but not bone metastasis (P = 0.71) (Supplemental Table 11). The multivariable Cox model further showed that the 3q gene signature was an independent predictor of metastases to lung (P < 2.0e-16, HR 1.58, 95% CI 1.42 to 1.76) or brain (P = 0.001, HR 1.61, 95% CI 1.21 to 2.13), after adjusting for age, ER, PR, HER2 and lymph node status, five common variables in the combined cohort (Supplemental Table 12). In addition, the 3q gene signature was significantly associated with lung metastases in basal-like (P < 2.0e-16), HER2 (P = 0.01) and luminal A (P = 0.01) subtypes, whereas was associated with brain metastases in basal-like (P < 2.0e-16) and luminal B (P = 1.94e-14) subtypes (Table 2). Basal-like breast cancer is known to have a large overlap with triple negative breast cancer, a subtype associated with distant metastasis. Further multivariable Cox model indicated that the 3q gene signature remained strongly associated with the risk of development of lung metastasis in TNBC (P = 8.48e-13, HR 1.44, 95% CI 1.31to 1.60) or basal like (P = 1.86e-07, HR 1.47, 95% CI 1.27 to 1.70) breast cancer patients after adjusting for age, node status and five known gene signatures (including two proliferating related signatures and three reported lung specific metastasis gene signatures), but not in non-TNBC breast cancer patients (P = 0.09, Table 2 and Supplemental Table 13). In contrast, the 3q gene signature was significantly associated with brain metastasis in both TNBC (P = 0.02) and non-TNBC patients (P = 8.46e-10, Table 2 and Supplemental Table 14). Together, these data suggest that the 3q gene signature is an independent prognostic marker for lung and brain specific metastasis in breast cancer, especially for lung metastasis in TNBC.

### 3q gene signature is associated with response to neoadjuvant chemotherapy

Among 4,801 patients, there are 1,058 patients in four datasets (GSE16446, GSE25066, GSE20194 and GSE20271) who had information on the response to neoadjuvant chemotherapy (Supplemental Table 15). All these patients received anthracycline (Epirubicin) monotherapy (GSE16446), taxane-anthracycline based (GSE25066) or paclitaxel followed by 5-fluorouracil, doxorubicin and cyclophosphamide (FAC) (GSE20194 and GSE20271) based neoadjuvant chemotherapy. In the combined dataset, multiple logistic regression analysis showed that the 3q gene signature was significantly associated with pathological complete response (pCR) after adjusting for clinical variables known to be associated with pCR including grade, status of ER, PR, HER2 and node (P < 0.0001, OR = 1.32, 95% CI 1.24 to 1.41, Supplemental Table 16). Further analysis showed that the 3q gene signature was associated with better pCR in TNBC (P < 0.0001, OR = 1.50, 95% CI 1.33 to 1.71) but worse pCR in non-TNBC patients (P = 0.35), after adjusting for known gene signatures and clinical variables (Supplemental Table 17). These data suggest that the 3q gene signature is an independent predictive biomarker for better response to neoadjuvant chemotherapy in TNBC.

### FXR1 protein overexpression predicts distant metastasis in TNBC

**Figure 3:**
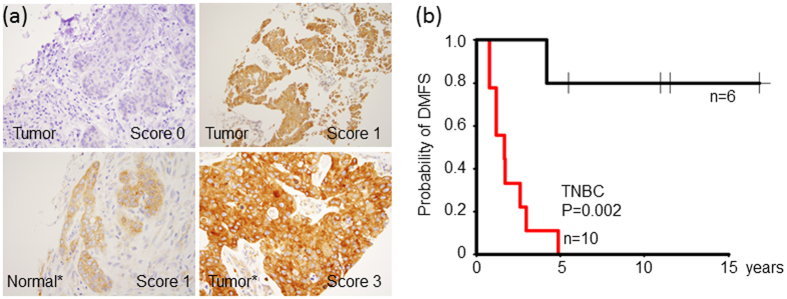
FXR1 overexpression was associated with poor DMFS in TNBC patients. (**a**) Representative immunohistochemical staining of FXR1 protein expression in sections of formalin-fixed paraffin-embedded breast tumor and adjacent normal* tissues. (**b**) Kaplan–Meier plot of DMFS of 16 TNBC patients stratified by median FXR1 protein expression. The log-rank P value is shown.

We previously identified FXR1 as a novel cancer gene that is associated with poor outcomes in multiple human cancers including lung and breast^9^. FXR1 was most significantly associated with lung metastasis only in TNBC (Supplemental Table 18), therefore we elected to validate this finding using immunohistochemistry on 69 breast tumors (Fig. 3 and Supplemental Table 19). Univariable Cox analysis showed that elevated FXR1 expression was associated with DMFS in TNBC (P = 0.01, HR, 9.63, 95% CI, 1.7–43.96, Fig. 3B) but not in non-TNBC (Supplemental Table 20). Multivariable Cox analysis further indicates that FXR1 protein expression was associated with DMFS after adjusting for tumor stage in TNBC (P = 0.03, HR, 6.37, 95% CI, 1.2–33.7, Supplemental Table 20). Together, these results suggest that FXR1 is a novel prognostic biomarker for distant metastasis in TNBC. Nonetheless, organ specific metastasis information was not available on these patients and thus we were unable to further analyze this association.

## Discussion

In a study including 27 publicly available gene expression datasets of clinically annotated breast cancer on a total of 4,801 patients, we found that the 3q 19-gene signature has strong association with breast cancer features of aggressiveness, reduced DMFS and response to neo-adjuvant chemotherapy, especially in triple negative breast cancer (TNBC). TNBC which are defined by a lack of ER, PR and HER2 expression, are still poorly characterized at the molecular level, and lack prognostic markers or targets for therapy^12^.

The majority of breast cancer deaths result from metastases rather than from direct effects of the primary tumor itself. The most common site of breast metastasis includes bone, lung and brain^1^. The prediction of a metastatic behavior remains a major challenge with only few metastasis-inducing proteins experimentally validated so far^6^. In a subset of 618 patients who had lung, brain and bone specific metastasis information available, the 3q 19-gene signature independently predicted breast cancer to metastasize to the lung or brain but not bone in TNBC. While gene signatures have been associated with breast outcome^13^, gene signatures that significantly associate with specific organ metastases are very limited^6^. Many gene signatures are not able to predict organ specific metastasis when adjusted for molecular subtype. For instance, the LMS 6-gene is highly correlated with basal subtype but its ability to predict lung metastasis is controversial^14^,^15^. Venet *et al*. showed that even randomly selected gene expression signatures could be significantly associated with breast cancer outcome, due to a high correlation with proliferation^16^. In contrast, the 3q gene signature is able to predict lung and brain metastasis in basal and luminal A/B. Furthermore, when compared with other lung metastasis signatures or well-studied proliferation signatures, the 3q gene signature maintained significance especially in TNBC. Likewise, in the setting of neoadjuvant chemotherapy, we found that the 3q gene signature was associated with pathological complete response (pCR) in TNBC patients. Together, the 3q gene signature provides additional prognostic information beyond the previously reported organ metastasis signatures, shedding new light on the possible biological processes relevant for predicting outcome or response to therapy in TNBC.

To date molecular mechanisms underlying breast cancer metastasis to the lungs and brain are still poorly understood. Our study highlights the molecular pathways possibly driven by 3q genes that might contribute to the organ preference of breast tumor cells for lung or brain. There have been studies showing that basal-like breast tumors represent a unique molecular entity strikingly similar to squamous cell lung cancers^17^, supporting clinical trials testing immune checkpoint agents in basal-like breast cancer as well as TNBC patients. We previously found the 3q gene signature is inversely associated with the suppressed immune response pathway in squamous carcinoma of the lung^18^. Whether and how 3q genes contribute to the regulation of immune pathway in human cancer warrants further study. Moreover, none of our 3q 19 genes overlaps with known gene signatures tested in this study, arguing a lack of efficacy of using these known gene signatures as prognostic factors in TNBC patients. Although the exact role of these 19 genes in breast cancer metastasis is unclear, 12 genes including FXR1 have been studied functionally or mechanistically in human cancers including lung and breast (Supplemental Table 21)^9^,^19^,^20^,^21^,^22^,^23^,^24^,^25^. For instance, PIK3CA is the most well-studied oncogene among our 3q gene signature due to its high mutation rate in human cancer, especially in breast cancer^23^. However, the prognostic value of PIK3CA mutation status in breast cancer is controversial^23^. PIK3CA mutation is associated with ER + and PR + breast cancer. A PIK3CA-mutated gene signature actually predicted better outcome in ER + breast cancer^26^. In contrast, our study showed that PIK3CA expression is significantly associated with lung or brain metastasis in TNBC (Supplemental Table 18). An analysis using TCGA breast cancer datasets showed that PIK3CA expression was indeed associated with copy number but not mutation (Supplemental Figure 5). It is PIK3CA amplification rather than PIK3CA mutation status that is associated with basal-like breast cancer (Supplemental Table 22). Early clinical trials also showed that PIK3CA mutations do not result in a dramatic responses to PI3K inhibitors^23^. Collectively, the significance of genotyping *PIK3CA* in clinical practice and targeted therapies based on the PIK3CA mutation in breast cancer remains unclear. Our study further argues that PIK3CA amplification or PIK3CA overexpression is more relevant than PIK3CA mutation status in breast cancer.

PRKCI is another well-known oncogene in human cancers and has been proposed to be a novel therapeutic target^27^. A recent study specifically demonstrated that PRKCI signaling promotes TNBC cell growth and metastasis through NF-κB pathway which could be regulated by TGFβ and IL1β^25^. We previously reported a new oncogenic role for RNA binding protein FXR1 linking to PRKCI and ECT2 in NSCLC^9^. In this study, multivariable cox analysis indicated that among 19 3q genes, FXR1 was actually most specifically associated with lung but not brain metastasis in TNBC (Supplemental Table 18). Therefore, we postulate that FXR1 is a novel prognostic biomarker specific for lung metastasis in TNBC and further validated the association of FXR1 protein with distant metastasis in TNBC using IHC on 69 breast tumors. We were not able to analyze organ specific metastasis due to the lack of such information on this small cohort of patients, a limitation we acknowledge. Future studies on a larger cohort are warranted.

Although the possible role of the 3q genes in specific steps of the metastatic process needs to be functionally validated, our study suggests an unrecognized role of certain 3q genes or gene interaction in the 3q amplicon in the basal cell type of human cancers and TNBC. A better knowledge of their function might lead to new insights into the mechanisms of disease progression and to the development of new predictive markers of metastatic behavior in breast cancer.

## Methods

### Breast tumor microarray datasets

We interrogated gene expression profiles from a total of 4,801 breast cancer patients in 27 publically available breast cancer datasets (Supplemental Table 1). 129 raw CEL files of E-TABM-158 (U133AAofAv2) were obtained from ArrayExpress and normalized using affy R package and Robust Multi-array Average (RMA) method^28^,^29^. Raw CEL files of GSE25066 (n = 508) were downloaded from the NCBI Gene Expression Omnibus (GEO) database and normalized using frozen Robust Multi-Array Analysis (fRMA) method, a procedure that allows one to pre-process microarrays individually or in small batches and to then combine the data into a single comparable dataset for further analyses^30^,^31^. The other 4,164 breast cancer gene expression profiles from 25 breast cancer datasets (GSE11121, GSE12093, GSE12276, GSE1456, GSE16391, GSE16446, GSE17705, GSE19615, GSE20194,GSE20271, GSE2034, GSE20685, GSE20711, GSE21653, GSE25066,GSE2603, GSE26971, GSE31519, GSE3494, GSE42568, GSE45255, GSE4922, GSE5327, GSE6532, GSE7390 and GSE9195) were obtained on the Affymetrix U133A or U133 Plus 2.0 expression array^11^,^30^,^31^,^32^,^33^,^34^,^35^,^36^,^37^,^38^,^39^,^40^,^41^,^42^,^43^,^44^,^45^,^46^,^47^,^48^,^49^,^50^,^51^,^52^,^53^,^54^,^55^,^56^,^57^. These samples were collected from InsilicoDB database^58^ and normalized using fRMA method. A final combined dataset compiling 4,801 breast tumor samples was generated using inSilicoMerging R package and COMBAT algorithm^59^, an Empirical Bayes method to adjust for potential batch effects in the dataset. TRANSBIG (n = 731) cohort consists of samples from GSE3494, GSE4922, GSE6532 and GSE7390 and the replicates within the studies were removed^49^. EMC344 (n = 344) consists of GSE2034 and GSE5327. After integration of the datasets, all gene expression profiles were filtered to include 21,172 probes on the HG-U133A platform and were further collapsed to 13,129 gene symbols. TCGA breast mutation (wustl curated), copy number (gistic2_thresholded) and mRNA expression dataset (IllumninaHiSeq_RNASeqV2) were downloaded using the UCSC Cancer Genomics Browser^60^ as described before^9^.

In addition to the raw expression data, we also obtained clinical outcome data from a subset of the samples (Supplemental Table 1), which included data on overall survival (OS, n = 990), recurrence-free survival (RFS, n = 1,814), distant metastasis free survival (DMFS, n = 3,715), as well as disease specific survival (DSS, event of death from breast cancer, n = 614). Four datasets included data on response to neoadjuvant chemotherapy (n = 1,028). For samples not characterized by immunohistochemistry (IHC) in this cohort, the final calls for estrogen receptor (ER) progesterone receptor (PR) and epidermal growth factor receptor 2 (HER2) statuses was defined by analyzing mRNA expression bimodal cutoffs in pooled 4801 samples using a 2-component Gaussian mixture distribution model and parameters were estimated by maximum likelihood optimization in optim R package^61^. PAM50 (basal-like, luminal A, luminal B, HER2-enriched, and normal-like) molecular subtype was calculated using genefu R package^62^,^63^. Triple-negative breast cancer (TNBC) was defined as ER-, PR- and HER2- based on mRNA expression cutoff.

### Breast tumor biospecimens

Breast cancer tissues were collected from surgical specimens through the Specialized Program of Research Excellence (SPORE) in breast at Vanderbilt University Medical Center in Nashville, Tennessee. All samples were reviewed by a pathologist (R.E). 69 breast cancer tissues contained in tumor tissue microarrays were used for the evaluation of FXR1 protein expression using immunohistochemistry as described before^9^. Clinical characteristics of these patients are described in Supplemental Table 19. All primary tumors were fresh-frozen, with efforts made to use samples with tumor content >70%. This study using human biospecimens was approved by the Vanderbilt University Internal Review Board and complied with all state, federal, and NIH regulations. Informed consent was obtained from all patients.

### Immunohistochemistry study

Immunohistochemical staining and scoring were performed as previously described^9^. Briefly, the staining index was considered as the sum of the intensity score (0, no staining; 1 + , weak; 2 + , moderate; 3 + , strong) and the distribution score (0, no staining; 0.1, staining of 1%-9% of cells; 0.5, 10%-49% and 1 if >50% of cells). The final immunoreactivity H score was determined by multiplying the intensity and extent of positivity scores of stained cells, with the minimum score of 0 and a maximum score of 3. The median value of all the H scores was a priori chosen as the cutoff point for separating FXR1-high tumors from FXR1-low tumors.

### Statistical analysis

Known gene signatures including MammaPrint (70-gene)^64^, Veridex (76-gene)^51^, GGI^65^ and Oncotype DX^66^ were calculated using genefu R package. 18-gene Lung metastasis signature (LMS) was defined as a linear combination of the gene-expression values weighted by their estimated regression coefficients obtained from univariable Cox proportional-hazard regression modeling as originally published^46^,^52^,^67^. The 3q 19-gene signature (19 out of 20 genes were mapped to the HG-U133A platform) and other published gene signatures including LMS 6-gene^14^, TGFβ pathway (152-gene)^67^ and PCNA (117-gene)^16^ were summarized to the mean expression within each sample and standardized to zero mean and unit variances before further analyses were performed. Pearson correlations between the signature indices were performed. Genes in signatures were mapped to the combined dataset by gene symbol.

Mann-Whitney-Wilcoxon test and Kruskal–Wallis one way analysis of variance (ANOVA) were used to compare the difference of gene signatures for groups of interest including dichotomized category clinical variables such as age (<50 or >50), tumor size (<2 cm or >2 cm), status of ER, PR, HER2 (positive vs negative), and lymph node (positive or negative), grade (grade III vs grade I/II), PAM50 subtypes, status of triple negative breast cancer (TNBC vs non-TNBC) and chemotherapy response (pathological complete response; pCR vs residual disease; RD). The distribution of gene signatures were visualized by using Box-and-whisker plots. Multiple logistic regression models were used to analyze the association between chemotherapy response and gene signatures as well as other clinical variables of interest. Cox proportional hazard (CPH) regression was used to analyze time to event data and the survival curve was calculated from Kaplan-Meier (KM) method. Robust sandwich covariance estimator was used for logistic and CPH regressions to account for the gene expression cluster (dataset) effect. The estimated odds ratio (OR), hazard ratio (HR) and 95% confidence intervals were provided to measure the effect of the association. In addition to the univariate CPH analysis, meta-analysis was conducted to confirm the finding in the univariate CPH regression. When 3q gene signature was used as dichotomized variable, the patients were divided into tertiles (high, intermediate and low) according to gene expression value. All p-values were based on two-sided tests and differences were considered statistically significant when p-value < 0.05. Analyses were performed using R version 3.3.1.

## Additional Information

**How to cite this article:** Qian, J. *et al*. A 3q gene signature associated with triple negative breast cancer organ specific metastasis and response to neoadjuvant chemotherapy. *Sci. Rep.* **7**, 45828; doi: 10.1038/srep45828 (2017).

**Publisher's note:** Springer Nature remains neutral with regard to jurisdictional claims in published maps and institutional affiliations.

## Supplementary Material

Supplementary Materials

Supplementary Dataset 1

## Figures and Tables

**Table 1:** Association between 3q gene signature and clinical parameters in 4,801 breast tumors.

Clinical parameters		Sample size	P value	HR^a^	95% C.I.
Age	<50 v>50	3675	0.07		
Grade	I/II v III	3176	<2.2e-16		
Tumor size	<2 v>2 cm	3528	0.005		
Nodal status	Yes v No	4554	0.26		
ER	Negative v Positive	3881	1.42e-08		
PR	Negative v Positive	2056	4.75e-09		
HER2	Negative v Positive	1777	0.53		
PAM50		4461	<2.2e-16		
TNBC	Yes vs No	4801	3.06e-12		
DMFS		3715	3.25e-05	1.19	1.10–1.3
RFS		1841	0.07	1.10	0.99–1.22
OS		990	0.29	1.06	0.95–1.17
DSS		614	0.24	1.13	0.93–1.37

^a^Hazard ratio (HR) was derived from univariable Cox analysis. ER, estrogen receptor; PR, progesterone receptor; HER2, epidermal growth factor receptor 2; TNBC, triple negative breast cancer; DMFS, distant metastasis free survival; RFS, recurrence-free survival; OS, overall survival; DSS, disease specific survival.

**Table 2:** Association between 3q gene signature and lung/brain metastasis free survival in subtypes of breast cancer.

Lung Met										
	Univariable Cox				Multivariable Cox				No. of	No. of
	HR	95%	C.I.	P value	HR	95%	C.I.	P value	events	patients
Basal-like^a^	1.37	1.28	1.46	<2.0e-16	1.47	1.27	1.7	1.86e-07	42	160
HER2^b^	3.29	1.39	7.82	0.01	3.65	0.93	14.31	0.06	8	57
Luminal A^b^	1.88	1.17	3.03	0.01	2.17	1.42	3.3	0.0003	10	204
Luminal B	1.29	0.71	2.35	0.41					21	174
Normal-like	1.08	0.82	1.42	0.58					4	23
TNBC^a^	1.39	1.34	1.45	<2.0e-16	1.44	1.31	1.6	8.48e-13	43	194
non-TNBC^a^	1.87	1.4	2.49	2.21E-05	1.36	0.95	1.96	0.09	42	424
Brain Met^b^										
Basal-like	1.79	1.57	2.04	<2.0e-16	1.87	1.57	2.22	1.2E-12	11	
HER2	0.89	0.15	5.25	0.89					3	
Luminal A	0.35	0.09	1.35	0.13					2	
Luminal B	4.4	3.02	6.41	1.19e-14	6.67	2.2	20.34	0.001	7	
Normal-like	1.16	0.21	6.25	0.87					5	
TNBC	1.46	1.15	1.87	0.002	1.5	1.07	2.12	0.02	12	
non-TNBC	2.45	2.01	2.99	<2.0e-16	2.44	1.83	3.24	8.46e-10	16	

a) Hazard ratio (HR) in multivariable Cox model was calculated and adjusted for age, node and four known gene signatures; b) HR in multivariable Cox model was adjusted for only age and node due to the limited number of events. C.I., confidence interval. The status of triple negative breast cancer (TNBC) was determined using microarray expression value of ER,PR and HER2 as described in the Methods.
