# Supplementary material for: A 3q gene signature associated with triple negative breast cancer organ specific metastasis and response to neoadjuvant chemotherapy
Source: Sci Rep. 2017 Apr 7;7:45828. doi: 10.1038/srep45828 (PMC5384279; doi:10.1038/srep45828)
Supplement: Supplementary Materials [file srep45828-s1.pdf]

Supplemental materials for the manuscript including:

1. **Supplemental Figures**
2. **Supplemental Tables**
3. **A Sweave file with R code and output for microarray expression analysis**
4. **R function code**

A 3q gene signature associated with triple negative breast cancer organ specific metastasis and response to neoadjuvant chemotherapy

Jun Qian[1,#*], Heidi Chen[2,#], Xiangming Ji[1], Rosana Eisenberg[3], A. Bapsi Chakravarthy[4], Ingrid A. Mayer[5], Pierre P. Massion[1,6,*].

[1]Division of Pulmonary and Critical Care Medicine, Department of Medicine, [2]Vanderbilt Center for Quantitative Sciences, Department of Statistics, Vanderbilt University Medical Center, [3]Department of Pathology, Microbiology and Immunology, Vanderbilt University, [4]Department of Radiation Oncology, [5]Divsion of Oncology, Department of Medicine, Vanderbilt-Ingram Cancer Center, Vanderbilt University Medical Center, Nashville, TN, [6]Veterans Affairs Medical Center, Nashville, TN,USA

Figure S1

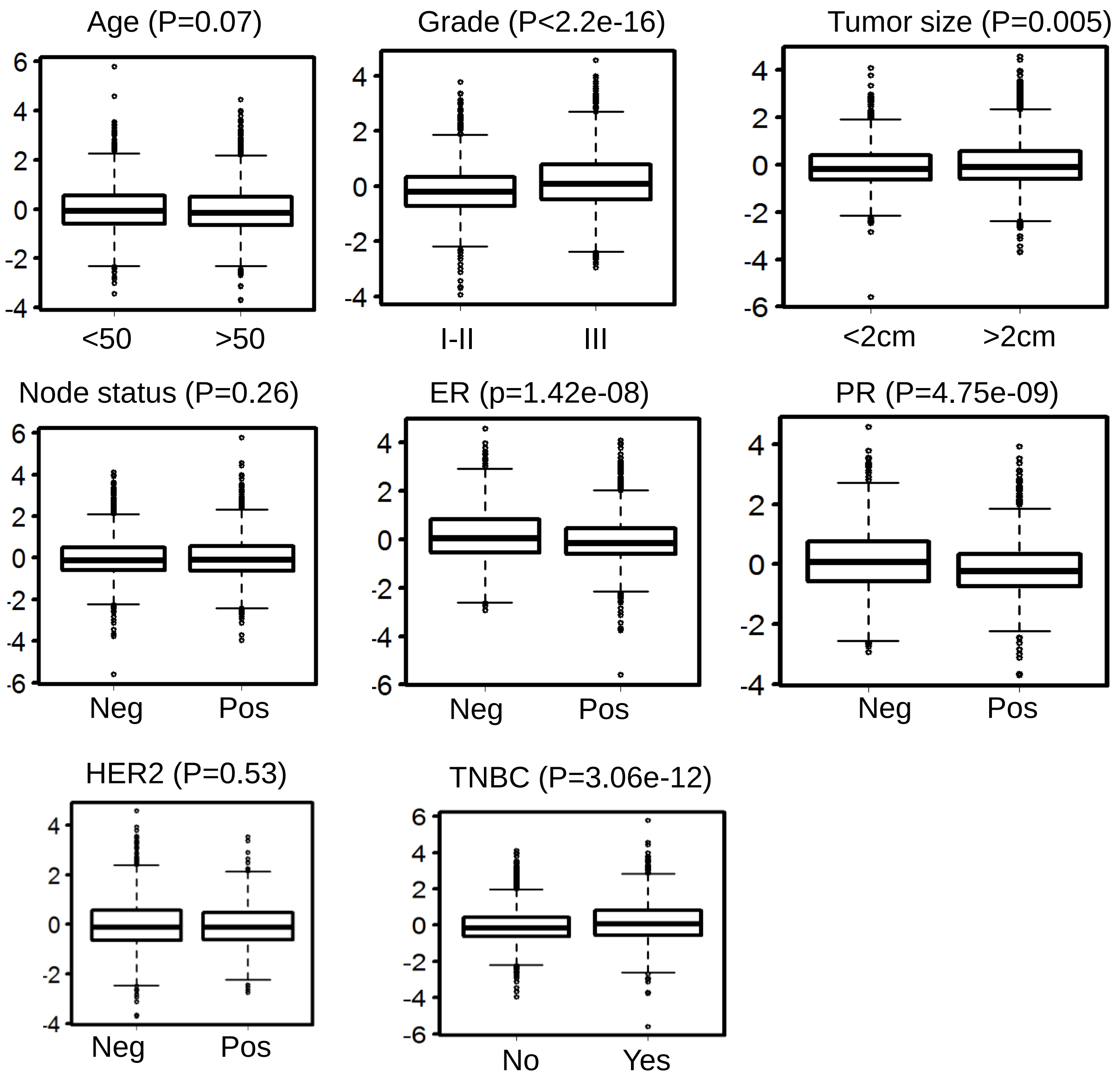

Age (P=0.07)
<50
>50
Grade (P<2.2e-16)
I-II
III
Tumor size (P=0.005)
<2cm
>2cm
Node status (P=0.26)
Neg
Pos
ER (p=1.42e-08)
Neg
Pos
PR (P=4.75e-09)
Neg
Pos
HER2 (P=0.53)
Neg
Pos
TNBC (P=3.06e-12)
No
Yes

Figure S2

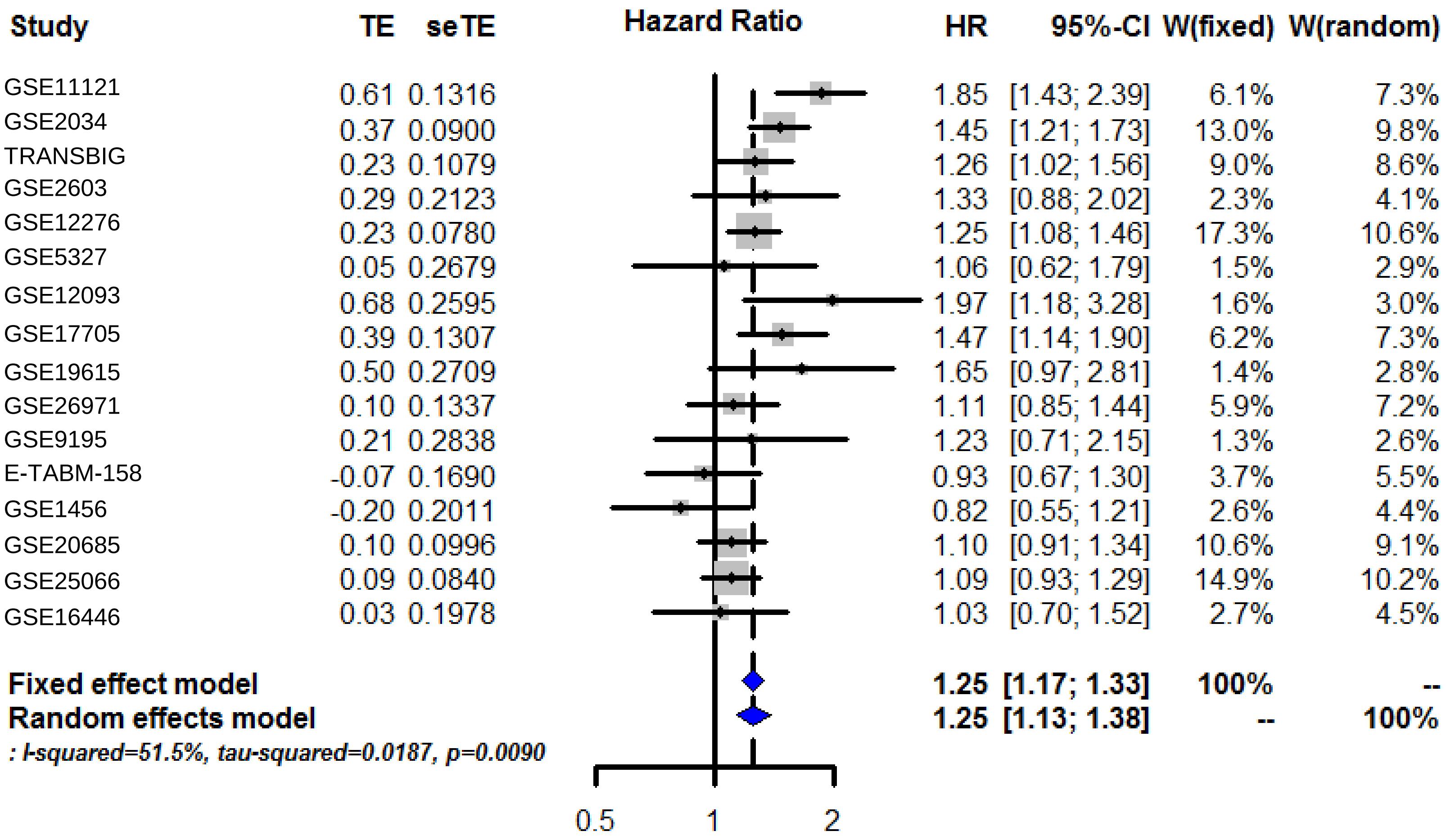
Study
TE
seTE
Hazard Ratio
HR
95%-CI
W(fixed)
W(random)
GSE11121 0.61 0.1316 1.85 [1.43; 2.39] 6.1% 7.3%
GSE2034 0.37 0.0900 1.45 [1.21; 1.73] 13.0% 9.8%
TRANSBIG 0.23 0.1079 1.26 [1.02; 1.56] 9.0% 8.6%
GSE2603 0.29 0.2123 1.33 [0.88; 2.02] 2.3% 4.1%
GSE12276 0.23 0.0780 1.25 [1.08; 1.46] 17.3% 10.6%
GSE5327 0.05 0.2679 1.06 [0.62; 1.79] 1.5% 2.9%
GSE12093 0.68 0.2595 1.97 [1.18; 3.28] 1.6% 3.0%
GSE17705 0.39 0.1307 1.47 [1.14; 1.90] 6.2% 7.3%
GSE19615 0.50 0.2709 1.65 [0.97; 2.81] 1.4% 2.8%
GSE26971 0.10 0.1337 1.11 [0.85; 1.44] 5.9% 7.2%
GSE9195 0.21 0.2838 1.23 [0.71; 2.15] 1.3% 2.6%
E-TABM-158 -0.07 0.1690 0.93 [0.67; 1.30] 3.7% 5.5%
GSE1456 -0.20 0.2011 0.82 [0.55; 1.21] 2.6% 4.4%
GSE20685 0.10 0.0996 1.10 [0.91; 1.34] 10.6% 9.1%
GSE25066 0.09 0.0840 1.09 [0.93; 1.29] 14.9% 10.2%
GSE16446 0.03 0.1978 1.03 [0.70; 1.52] 2.7% 4.5%
Fixed effect model 1.25 [1.17; 1.33] 100% --
Random effects model 1.25 [1.13; 1.38] -- 100%
: I-squared=51.5%, tau-squared=0.0187, p=0.0090
0.5
1
2

Figure S3

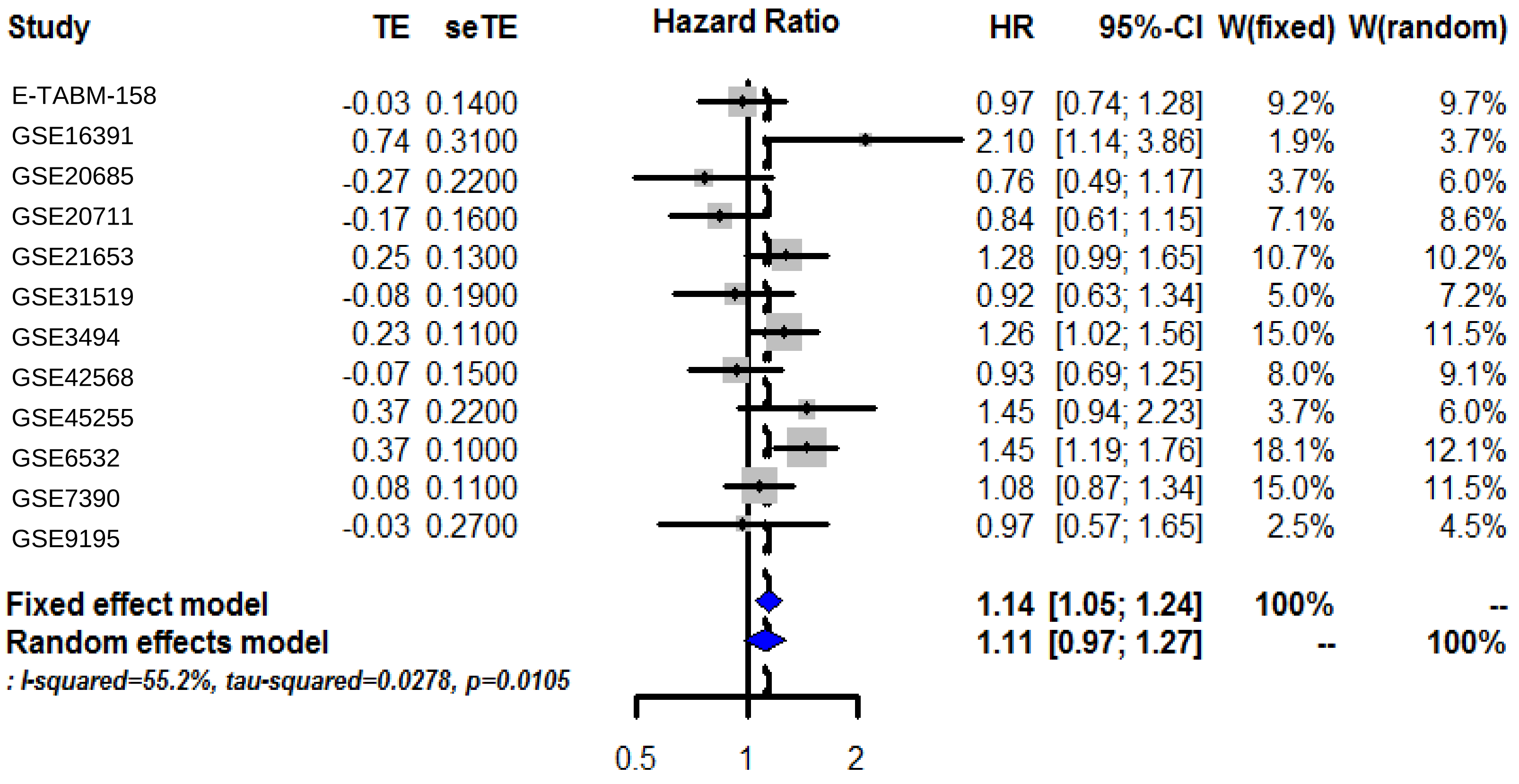
Study
TE
seTE
Hazard Ratio
HR
95%-CI
W(fixed)
W(random)
E-TABM-158 -0.03 0.1400 0.97 [0.74; 1.28] 9.2% 9.7%
GSE16391 0.74 0.3100 2.10 [1.14; 3.86] 1.9% 3.7%
GSE20685 -0.27 0.2200 0.76 [0.49; 1.17] 3.7% 6.0%
GSE20711 -0.17 0.1600 0.84 [0.61; 1.15] 7.1% 8.6%
GSE21653 0.25 0.1300 1.28 [0.99; 1.65] 10.7% 10.2%
GSE31519 -0.08 0.1900 0.92 [0.63; 1.34] 5.0% 7.2%
GSE3494 0.23 0.1100 1.26 [1.02; 1.56] 15.0% 11.5%
GSE42568 -0.07 0.1500 0.93 [0.69; 1.25] 8.0% 9.1%
GSE45255 0.37 0.2200 1.45 [0.94; 2.23] 3.7% 6.0%
GSE6532 0.37 0.1000 1.45 [1.19; 1.76] 18.1% 12.1%
GSE7390 0.08 0.1100 1.08 [0.87; 1.34] 15.0% 11.5%
GSE9195 -0.03 0.2700 0.97 [0.57; 1.65] 2.5% 4.5%
Fixed effect model 1.14 [1.05; 1.24] 100% --
Random effects model 1.11 [0.97; 1.27] -- 100%
: I-squared=55.2%, tau-squared=0.0278, p=0.0105
0.5
1
2

Figure S4

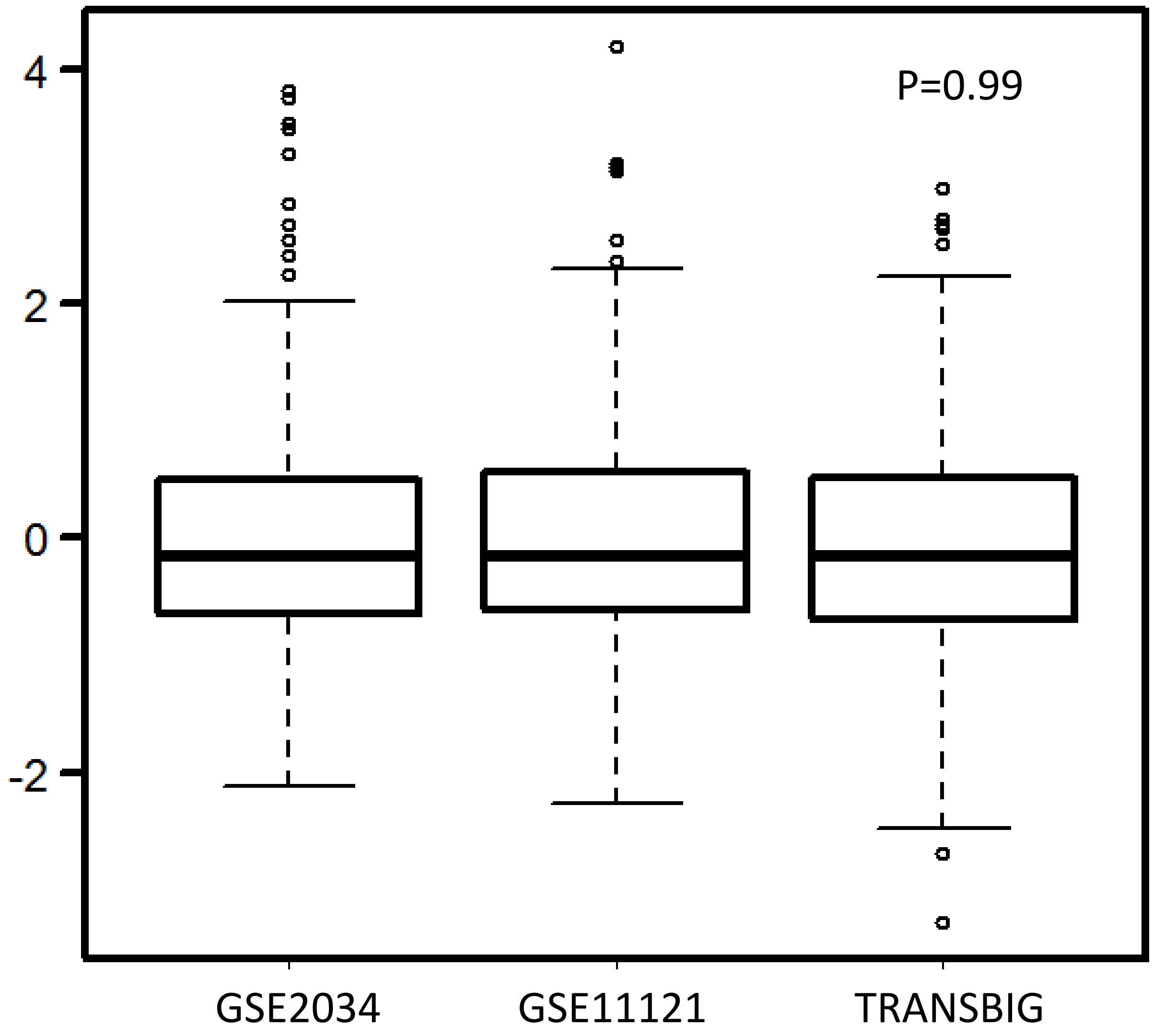

Figure S5

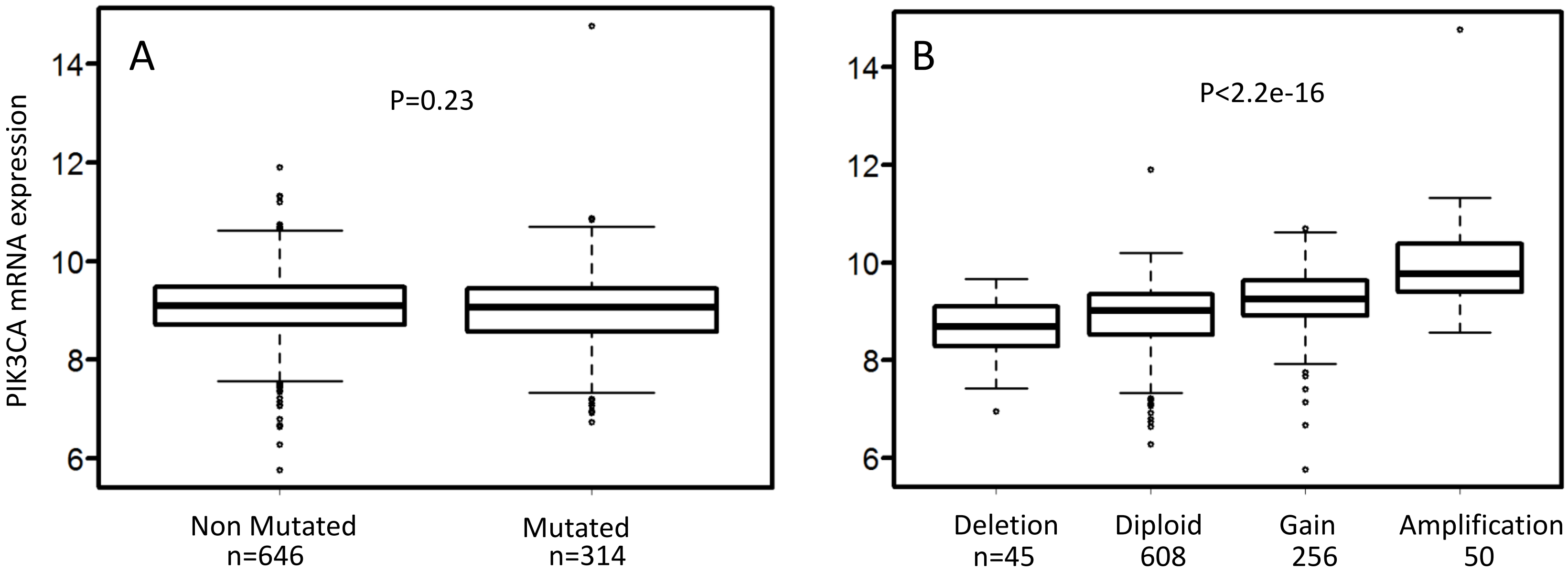

A
P=0.23
PIK3CA mRNA expression
14
12
10
8
6
Non Mutated
n=646
Mutated
n=314
B
P<2.2e-16
14
12
10
8
6
Deletion
n=45
Diploid
608
Gain
256
Amplification
50

Supplemental Figure legend

Supplemental Figure 1. Association between 3q gene signature and clinical parameters in 4,801 breast tumors. Neg, negative; Pos, positive. ER, estrogen receptor; PR, progesterone receptor; HER2, epidermal growth factor receptor 2; TNBC, triple negative breast cancer. P value was calculated using student T test.

Supplemental Figure 2. Forest plot of hazard ratios and distant metastasis free survival plot of 3q gene signature in 16 breast cancer datasets. Gray squares indicate hazard ratios (HRs), and solid horizontal lines represent 95% confidence intervals (CIs). The vertical solid line indicates point of no effect. Blue diamond indicates overall effect.

Supplemental Figure 3. Forest plot of hazard ratios and distant metastasis free survival plot of 3q gene signature in 12 breast cancer datasets. Gray squares indicate hazard ratios (HRs), and solid horizontal lines represent 95% confidence intervals (CIs). The vertical solid line indicates point of no effect. Blue diamond indicates overall effect.

Supplemental Figure 4. Level of 3q 19-gene signature was no difference in three node-negative breast cancer datasets. P value was calculated using Kruskal–Wallis one-way analysis of variance (ANOVA) analysis.

Supplemental Figure 5. PIK3CA mRNA expression is correlated to its copy number (CN) alterations but not mutation status in The Cancer Genome Atlas (TCGA) breast cancer (n=960). CN alterations were derived from GISTIC score. "-1" is deletion, "0" is diploid, "1" indicates a low-level gain, and "2" is a high-level amplification.

Table S1. Summary of 27 Affymetrix microarray datasets used in this study

Dataset	No. of patients	Number of events														Refs.
		DMFS		RFS		OS		DSS		Hormone		Chemo		pCR		
		No	Yes	No	Yes	No	Yes	No	Yes	No	Yes	No	Yes	No	Yes	
E-TABM-158	129	101	27	89	39			99	29	53	74	60	68			11
GSE11121	200	154	46							200		200				15
GSE12093	136	116	20								136					16
GSE12276	192	19	173													17
GSE1456	159	119	40			119	40	130	29							18
GSE16391	55			38	10						55	35	20			19
GSE16446	120	89	25			98	16						120	98	16	20
GSE17705	298	227	71								298					21
GSE19615	115	101	14							47	64	28	81			22
GSE20194	248												248	198	50	23
GSE20271	178												178	152	26	24
GSE2034	286	179	107							286		286				40
GSE20685	327	244	83	282	25	244	83					54	268			25
GSE20711	88			49	39	63	25									26
GSE21653	266			169	83											27
GSE25066	508	397	111										508	389	99	13
GSE2603	82	55	27													28
GSE26971	277	200	58								277					29
GSE31519	67			41	23							9	58			30
GSE3494	230	164	66	143	87	0	0	178	52	150	80	206	24			31
GSE42568	104			56	48	69	35									35
GSE45255	100	73	27	44	15			81	16	39	57	39	57			36
GSE4922	38	2		2												32
GSE5327	58	47	11													41
GSE6532	265	183	64	170	87					69	196	265				33
GSE7390	198	136	62	107	91	142	56			198		198				34
GSE9195	77	67	10	64	13						77					37
Total	4801	2673	1042	1254	560	735	255	488	126	1100	1314	1380	1630	837	191	

DMFS, distant metastasis free survival; RFS, recurrence-free survival; OS, overall survival; DSS, disease specific survival. Hormone, hormone treatment. Chemo, chemotherapy, including neoadjuvant , adjuvant and other unspecified chemotherapy. pCR, pathological complete response

Table S2. Summary of node-negative breast cancer datasets (n=788)

Dataset	Age		Tumor size		Grade		ER		PR		HER2		Distant Met.		No. of Patients
	<50	>50	<2	>2cm	I-II	III	Neg	Pos	Neg	Pos	Neg	Pos	No	Yes	
GSE11121	47	153	99	101	165	35	38	162					154	46	200
GSE2034	120	166			49	148	77	209	107	166	207	46	179	107	286
TRANSBIG	174	128	107	173	178	105	93	203	11	34			211	91	302
Total	341	447	206	274	392	288	208	574	118	200	207	46	544	244	788

Neg, negative; Pos, positive; ER, estrogen receptor; PR, progesterone receptor; HER2, epidermal growth factor receptor 2; Met, metastasis.

Table S3. Univariable Cox analysis of 3q gene signature for DMFS in node-negative breast cancer datasets

Dataset	HR	95% C.I.		P
GSE11121	1.84	1.43	2.39	3.25e-06
GSE2034	1.45	1.21	1.73	3.97e-05
TRANSBIG	1.26	1.02	1.56	0.03

HR, hazard ratio; DMFS, distant metastasis free survival.

Table S4. Multivariable Cox analysis for DMFS in node-negative breast cancer datasets

Covariables	GSE2034				GSE11121				TRANSBIG				GSE11121+TRANSBIG				GSE2034+ GSE11121+TRANSBIG			
	HR	95% C.I.		P	HR	95% C.I.		P	HR	95% C.I.		P	HR	95% C.I.		P	HR	95% C.I.		P
3q 19-gene	1.38	1.11	1.72	0.004	1.83	1.41	2.37	6.45e-06	1.18	0.95	1.47	0.13	1.36	1.04	1.78	0.03	1.36	1.16	1.58	0.0001
ER (Pos vs Neg)	1.41	0.82	2.42	0.21	0.67	0.33	1.35	0.26	0.70	0.42	1.16	0.17	0.69	0.64	0.73	<2e-16	0.95	0.65	1.40	0.81
PR (Pos vs Neg)	0.54	0.30	0.99	0.05	1.26	0.59	2.69	0.55	0.77	0.44	1.35	0.37	0.85	0.66	1.09	0.20	0.76	0.60	0.97	0.03
HER2 (Pos vs Neg)	0.79	0.41	1.52	0.48	1.84	0.87	3.88	0.11	0.91	0.51	1.64	0.76	1.16	0.75	1.80	0.50	0.96	0.72	1.28	0.77
Grade (III vs I-II)	2.26	1.14	4.48	0.02	1.57	0.73	3.36	0.25	0.74	0.44	1.25	0.26	0.92	0.60	1.40	0.70	1.54	0.94	2.52	0.09
Age (>50 vs <50)	0.81	0.50	1.32	0.40	1.40	0.70	2.82	0.34	1.13	0.71	1.80	0.62	1.12	1.02	1.22	0.01	1.09	0.93	1.29	0.29
Tumor size (> 2 vs <2 cm)					0.94	0.49	1.79	0.84	3.12	1.75	5.57	0.0001	1.89	0.88	4.07	0.10				

HR, hazard ratio; ER, estrogen receptor; PR, progesterone receptor; HER2, epidermal growth factor receptor 2; Pos, positive; Neg, negative; DMFS, distant metastasis free survival. The status of ER, PR and HER2 was determined using mRNA expression cutoff.

Table S5. Multivariable Cox proportional hazards analysis for DMFS in combined node-negative breast cancer datasets

Covariables	HR	95% C.I.		P value
3q 19-gene	1.38	1.23	1.55	8.43e-08
Basal like	1.76	1.27	2.44	0.00071
HER2	1.95	1.23	3.11	0.00474
Luminal A	1.09	0.74	1.62	0.66196
Luminal B	2.25	1.54	3.30	2.96e-05

DMFS, distant metastasis free survival; HR, hazard ratio; C.I., confidence interval. Normal-like subtype was used as baseline for HR calculation.

Table S6. Multivariable Cox proportional hazards analysis for DMFS in combined node-negative breast cancer datasets

Covariables	HR	95% C.I.		P value
3q 19-gene	1.24	1.14	1.36	7.80e-07
GENE70	1.62	0.51	5.18	0.42
GENE76	1.01	1.01	1.02	0.0001
GGI	2.04	0.23	17.90	0.52
Oncotype DX	1.00	0.99	1.01	0.89
PCNA 117-gene	0.66	0.26	1.66	0.38

DMFS, distant metastasis free survival. ; HR, hazard ratio; C.I., confidence interval.

Table S7. Multivariable Cox proportional hazards analysis for DMFS in combined node-negative breast cancer datasets

Covariables	HR	95% C.I.		P value
3q 19-gene	1.26	1.17	1.36	2.96e-09
T cell metagene	0.94	0.90	0.99	0.017
B cell metagene	0.71	0.67	0.75	<2.0e-16
Proliferation metagene	1.22	1.19	1.26	<2.0e-16
ER metagene	0.88	0.81	0.96	0.003

DMFS, distant metastasis free survival; HR, hazard ratio. C.I., confidence interval. ER, estrogen receptor.

Table S8. Univariable Cox proportional hazards analysis of 3q gene signature for DMFS in PAM50 subtypes of node-negative breast cancer patients.

Subtype	HR	95% C.I.		P
Basal-like	1.27	1.19	1.37	3.48e-11
Luminal B	1.66	1.53	1.80	<2e-16
Luminial A	1.02	0.65	1.59	0.94
HER2	1.40	1.01	1.94	0.04
Normal-like	2.26	1.30	3.95	0.004

DMFS, distant metastasis free survival; HR, hazard ratio. C.I., confidence interval.

Table S9. Multivariable Cox proportional hazards analysis for DMFS in Basal-like (n=137), Luminal B (n=254), HER2 (n=81) and Normal-like (n=28) subtypes of node-negative breast cancer patients

	Basal-like				Luminal B				HER2				Normal-like			
Covariables	HR	95% C.I.		P	HR	95% C.I.		P	HR	95% C.I.		P	HR	95% C.I.		P
3q 20-gene	1.5	1.4	1.6	8.88E-16	1.4	1.2	1.6	1.06E-07	1.26	0.84	1.90	0.27	3.37	0.35	32.15	0.29
B cell metagene	0.6	0.6	0.7	<2.0e-16	0.7	0.6	0.8	1.04E-05	0.53	0.42	0.66	4.01E-08	0.21	0.09	0.51	0.0005
Proliferation metagene	0.6	0.4	0.8	0.001	1.5	1.1	2.1	0.02	0.57	0.29	1.12	0.1	19.53	0.37	1030.47	0.14

DMFS, distant metastasis free survival; HR, hazard ratio. C.I., confidence interval.

Table S10. Summary of three breast cancer datasets that had lung, brain and bone metastasis information (n=618)

Dataset	Age		Grade		Tumor size		Node		ER*		PR*		HER2*		TNBC*		Distant Met.			No. of Patients
	<50	>50	I-II	III	<2	>2cm	Neg	Pos	Neg	Pos	Neg	Pos	Neg	Pos	No	Yes	Lung	Brain	Bone	
EMC344	120	166	49	148			344		159	185	237	107	274	70	240	104	31	10	69	344
GSE12276	91	98					48	144	89	103	136	56	148	44	129	63	40	13	102	192
GSE2603	30	52			7	75	28	54	36	46	57	25	61	21	55	27	14	5	14	82
Total	241	316	49	148	7	75	420	198	284	334	430	188	483	135	424	194	85	28	185	618

*The status of ER, PR , HER2 and TNBC was determined using microarray expression value of ER,PR and HER2 as described in the Methods. Neg, negative. Pos, positive. ER, estrogen receptor. PR, progesterone receptor. HER2, epidermal growth factor receptor 2. TNBC, triple negative breast cancer. Met, metastasis.

Table S11. Univariable Cox proportional hazards analysis of 3q 19-gene signature for DMFS in breast cancer datasets that had lung ,brain and bone metastasis information.

Dataset	HR	95% C.I.		P value	
EMC344	1.68	1.24	2.28	0.0008	
GSE12276	1.88	1.42	2.48	8.58e-06	
GSE2603	2.35	1.33	4.15	0.0032	(lung)
Combined	1.74	1.60	1.9	<2.0e-16	
EMC344	2.50	1.62	3.84	3.17e-05	
GSE12276	1.91	1.19	3.09	0.008	
GSE2603	1.35	0.51	3.58	0.55	(brain)
Combined	1.99	1.62	2.45	4.79e-11	
EMC344	1.34	1.06	1.68	0.013	
GSE12276	1.00	0.81	1.23	0.99	(bone)
GSE2603	0.76	0.42	1.40	0.38	
Combined	1.04	0.83	1.31	0.71	

HR, hazard ratio. C.I., confidence interval. DMFS, distant metastasis free survival.

Table S12. Multivariable Cox proportional hazards analysis for lung and brain metastasis free survival in breast cancer dataset (n=618)

Covariables	Lung metastasis				Brain metastasis			
	HR	95% C.I.		P	HR	95% C.I.		P
3q 19-gene	1.58	1.42	1.76	<2.0e-16	1.61	1.21	2.13	0.001
Age (>50 vs <50)	1.19	0.82	1.72	0.36	0.98	0.43	2.24	0.96
Node (Pos vs Neg)	2.82	1.00	7.97	0.05	3.04	1.14	8.09	0.03
ER (Pos vs Neg)*	0.49	0.18	1.27	0.14	0.39	0.31	0.48	<2.0e-16
PR (Pos vs Neg)*	0.63	0.50	0.79	6.19e-05	0.30	0.06	1.38	0.12
HER2 (Pos vs Neg)*	0.75	0.37	1.52	0.42	0.60	0.47	0.77	4.70e-05

*The status was determined using microarray expression value of ER,PR and HER2 as described in the Methods.

Table S13. Multivariable Cox proportional hazards analysis for lung metastasis free survival in subtypes of breast cancer (n=618)

Covariables	TNBC (n=194)				Non-TNBC (n=424)				Basal-like (n=157)			
	HR	95% C.I.		P	HR	95% C.I.		P	HR	95% C.I.		P
3q 19-gene	1.44	1.31	1.60	8.48e-13	1.36	0.95	1.96	0.09	1.47	1.27	1.70	1.86e-07
GENE70	0.19	0.06	0.60	0.005	34.55	0.28	4232.84	0.15	0.22	0.14	0.34	8.93e-11
GENE76	1.02	1.01	1.02	1.98e-10	1.02	0.99	1.04	0.21	1.01	1.00	1.02	0.0531
LMS 18-gene	1.17	1.07	1.29	0.001	1.05	0.91	1.22	0.47	1.17	0.99	1.37	0.0592
TGFb	1.11	0.78	1.58	0.56	1.76	1.15	2.69	0.01	1.27	0.87	1.86	0.2119
LMS 6-gene	0.98	0.76	1.26	0.87	0.61	0.38	1.00	0.05	1.13	0.88	1.46	0.3357
Age (>50 vs <50)	0.77	0.52	1.12	0.17	2.16	1.77	2.63	3.74E-14	0.76	0.47	1.22	0.2528
Node (Pos vs Neg)	4.67	2.36	9.22	9.28e-06	3.25	0.93	11.31	0.06	5.24	4.39	6.27	<2.0e-16

* HR, harzad ratio. C.I., confidence interval. TNBC status was determined using microarray expression value of ER,PR and HER2 as described in the Methods.

Table S14. Multivariable Cox proportional hazards analysis for brain metastasis free survival in subtypes of breast cancer (n=618)

Covariables	TNBC (n=191)				Non-TNBC (n=424)				Basal-like (n=157)			
	HR	95% C.I.		P	HR	95% C.I.		P	HR	95% C.I.		P
3q 19-gene	1.50	1.07	2.12	0.02	2.44	1.83	3.24	8.46e-10	1.87	1.57	2.22	1.20e-12
Age (>50 vs <50)	0.77	0.26	2.26	0.63	1.53	1.03	2.26	0.03	1.04	0.33	3.34	0.94
Node (Pos vs Neg)	2.27	1.06	4.89	0.04	4.89	1.53	15.6	0.007	2.64	1.04	6.69	0.04

* HR, harzad ratio. C.I., confidence interval. TNBC status was determined using microarray expression value of ER,PR and HER2 as described in the Methods.

Table S15. Summary of four datasets of breast cancer patients who received neoadjuvant chemotheropy (n=1054)

Dataset	Age		Tumor size		Grade		Node		ER		PR		HER2		TNBC		pCR		Distant Met.		Total
	<50	>50	<2	>2	I-II	III	Neg	Pos	Neg	Pos	Neg	Pos	Neg	Pos	No	Yes	RD	pCR	No	Yes	
GSE16446			17	103	22	92	55	65					62	31	82	38	98	16	89	25	120
GSE20194	107	140	24	222	109	128	70	177	102	146	139	109	205	43	161	87	198	50			248
GSE20271	94	84	13	164	76	72	59	118	80	98	95	83	152	26	110	68	152	26			178
GSE25066	264	244	33	475	212	259	157	351	208	300	258	243	479	29	296	212	389	99	397	111	508
Total	482	451	87	964	419	551	341	711	508	546	590	451	898	129	649	405	837	191	486	136	1054

Neg, negative; Pos, positive; ER, estrogen receptor; PR, progesterone receptor; HER2, epidermal growth factor receptor 2; Met, metastasis. The status of triple negative breast cancer (TNBC) was determined using microarray expression value of ER,PR and HER2.

Table S16. Multivariable logistic regression analysis for variables associated with pCR in combined datasets of breast cancer patients who received neoadjuvant chemotherapy

Covariables	OR	95% C.I.		P
3q 19-gene	1.32	1.24	1.41	<0.0001
Grade (III vs I-II)	3.26	2.99	3.55	<0.0001
Age (>50 VS <50)	0.73	0.63	0.84	<0.0001
Tumor size (>2 vs <2 cm)	0.59	0.32	1.09	0.09
Node status (Pos vs Neg)	1.27	0.86	1.87	0.23
ER (Pos vs Neg)	0.25	0.17	0.36	<0.0001
PR (Pos vs Neg)	0.78	0.39	1.57	0.49
HER2 (Pos vs Neg)	1.39	0.55	3.51	0.48

OD, odds ratio. C.I., confidence interval. Neg, negative; Pos, positive; ER, estrogen receptor; PR, progesterone receptor; HER2, epidermal growth factor receptor 2. The status of ER ,PR and HER2 was determined using microarray expression value.

Table S17. Multivariable logistic regression analysis for variables associated with pCR in TNBC and non-TNBC patients who received neoadjuvant chemotherapy

Covariables	TNBC (n=405)				Non-TNBC (n=649)			
	OR	95% C.I.		P	OR	95% C.I.		P
3q 19-gene	1.50	1.33	1.71	2.33e-10	0.85	0.78	0.92	0.0002
Age (>50 VS <50)	0.61	0.48	0.76	2.03e-05	1.30	0.85	1.99	0.23
Tumor size (>2 vs <2 cm)	0.89	0.60	1.31	0.54	0.43	0.16	1.20	0.11
Grade (III vs I-II)	1.94	1.49	2.53	1.05e-06	3.63	2.57	5.11	1.82e-13
Node status (Pos vs Neg)	1.46	0.70	3.04	0.31	1.17	0.96	1.42	0.12
GENE70	10.20	4.90	21.22	5.19e-10	2.91	0.10	85.28	0.53
GGI	0.10	0.05	0.17	1.11e-15	10.53	4.92	22.53	1.31e-09
PCNA 117-gene	2.35	1.95	2.83	0.00	0.56	0.38	0.81	0.003
LMS 6-gene	1.30	1.13	1.51	0.0003	1.53	1.22	1.93	0.0002

OD, odds ratio. C.I., confidence interval. Neg, negative; Pos, positive; ER, estrogen receptor; PR, progesterone receptor; HER2, epidermal growth factor receptor 2. The status of ER ,PR, HER2 and TNBC was determined using microarray expression value.

Table S18. Multivariable Cox analysis of 19 3q genes for lung and brain metastasis free survival in breast cancer

	Lung metastasis[a]						Brain metastasis[b]					
	TNBC			Non-TNBC			TNBC			Non-TNBC		
	HR	95% CI	P	HR	95% CI	P	HR	95% CI	P	HR	95% CI	P
PIK3CA	1.39	1.32 1.47	<2E-16	1.47	0.93 2.34	0.10	1.72	1.35 2.19	1.0E-05	1.40	0.70 2.80	0.34
**FXR1**	1.64	1.39 1.92	**2E-09**	1.53	0.80 2.92	**0.20**	1.50	0.80 2.80	**0.21**	1.33	0.49 3.61	**0.58**
MFN1	1.83	1.41 2.36	4E-06	1.52	1.33 1.73	6.7E-10	1.65	0.73 3.72	0.23	1.49	0.37 6.00	0.57
PSMD2	2.45	1.67 3.60	4E-06	1.79	0.97 3.33	0.06	2.96	1.23 7.12	0.02	6.09	1.64 22.60	0.007
NDUFB5	3.49	1.95 6.24	2E-05	2.02	1.29 3.16	0.002	5.43	1.92 15.32	0.001	2.74	1.57 4.76	0.0004
ATP11B	2.20	1.48 3.27	0.0001	1.00	0.90 1.11	1.00	2.51	1.38 4.55	0.003	1.68	1.11 2.55	0.01
ZNF639	29.3	3.00 285.81	0.004	2.41	0.10 59.28	0.59	92.07	26.77 316.72	7.2E-13	0.40	0.01 27.64	0.68
**ABCC5**	2.02	1.20 3.39	**0.008**	1.14	0.68 1.91	**0.63**	0.82	0.18 3.70	**0.80**	1.13	0.98 1.30	**0.11**
EIF2B5	2.09	1.20 3.65	0.01	1.03	0.47 2.28	0.94	2.18	1.17 4.06	0.01	1.62	0.72 3.63	0.24
NCBP2	1.36	1.05 1.77	0.02	0.82	0.53 1.27	0.37	1.54	1.01 2.35	0.05	1.25	0.82 1.90	0.30
ACTL6A	1.62	1.01 2.60	0.04	1.33	0.92 1.92	0.13	1.63	0.87 3.06	0.13	1.76	1.50 2.08	8.45E-12
PRKCI	1.40	0.95 2.07	0.09	1.03	0.71 1.50	0.86	1.38	0.60 3.18	0.45	1.96	0.78 4.93	0.16
DCUN1D1	1.58	0.87 2.86	0.13	1.48	0.70 3.13	0.30	4.43	3.76 5.23	<2e-16	1.55	0.22 11.06	0.66
DVL3	1.69	0.61 4.66	0.31	0.71	0.31 1.63	0.42	2.06	1.93 2.21	<2e-16	0.88	0.35 2.19	0.78
SENP2	1.48	0.58 3.76	0.41	2.46	0.70 8.61	0.16	0.87	0.74 1.04	0.12	4.69	1.30 16.88	0.02
SENP5	1.27	0.55 2.92	0.57	1.16	0.66 2.02	0.61	3.41	1.04 11.12	0.04	2.64	2.18 3.19	<2e-16
LSG1	1.32	0.51 3.46	0.57	1.08	0.53 2.22	0.82	1.33	0.28 6.38	0.72	2.78	1.65 4.70	0.0001
UBXN7	1.05	0.85 1.28	0.66	2.48	1.77 3.46	1.E-07	1.99	1.24 3.19	0.004	0.51	0.30 0.88	0.01
DLG1	0.97	0.52 1.81	0.93	1.01	0.83 1.21	0.96	2.96	1.05 8.36	0.04	1.70	0.14 20.50	0.68

a. HR was adjusted for age, node and five proliferation gene signatures. b. HR was adjusted for age, node and PCNA proliferation gene signature. TNBC, triple negative breast cancer. HR, hazard ratio. CI, confidence interval.

Table S19 . Clinical characteristics of 69 breast cancer patients for IHC study

Variables		No. of patients
Age	>50	41
	<50	25
Stage	I	20
	II	24
	III	17
Size	>2 cm	55
	<2 cm	8
Node status	Negative	29
	Positive	32
ER	Negative	23
	Positive	35
HER2	Negative	28
	Positive	8
PR	Negative	21
	Positive	30
TNBC	Negative	42
	Positive	16
Outcome events		
OS		38
DMFS		29
RFS		23
Neoadjuvant chemotherapy	Yes	4
	No	62
Hormone therapy	Yes	30
	No	29

ER, estrogen receptor; PR, progesterone receptor; HER2, epidermal growth factor receptor 2. DMFS, distant metastasis free survival; RFS, recurrence-free survival; OS, overall survival;

Table S20. Cox proportional hazards analysis for association of FXR1 protein expression with DMFS, OS and RFS in breast cancer

	Univariable								Multivariable*			
	TNBC (n=16)				Non-TNBC (n=42)				TNBC			
	HR	95% CI		P	HR	95% CI		P	HR	95% CI		P
DMFS	8.63	1.69	43.96	0.01	0.93	0.50	1.72	0.82	6.37	1.20	33.73	0.03
OS	4.11	1.16	14.57	0.03	1.19	0.67	2.13	0.55	3.81	1.03	6.17	0.04
RFS	7.25	1.01	52.23	0.05	0.90	0.46	1.75	0.75	7.63	0.95	17.69	0.06

DMFS, distant metastasis free survival; RFS, recurrence-free survival; OS, overall survival. HR, hazard ration.
*HR was adjusted for stage in multivariable Cox model.

Table S21. The number of literatures on biological role for 19 3q genes in human cancers

	Lung cancer	Brest cancer	Ovarian cancer	human cancers	Function/mechanism study	Function implication
ABCC5	14	32	5	134	Yes*	drug resistance[20]
ACTL6A		2		10	Yes	
ATP11B	3	4	1	8	Yes	
DCUN1D1	8	1		18	Yes	
DLG1	3	6		72	Yes	
DVL3	13	7		44	Yes*	metastasis[21]
EIF2B5	1	1	1	5		
FXR1	5	2	2	28	Yes*	invasion[9]
LSG1		1		1		
MFN1	1	2		37	Yes*	Invasion[22]
NCBP2		1	1	3		
NDUFB5	2	6		8		
PIK3CA	408	659	207	2823	Yes*	invasion2[4], etc
PRKCI	8	2	1	22	Yes*	metastasis[26]
PSMD2	2	1		6		
SENP2	3	5		40	Yes*	repression of estrogen receptor[25]
SENP5		1		6	Yes*	invasion[23]
UBXN7	1		1	2		
ZNF639	2			8		

*Studies were done using breast cancer cell lines or tissues.

Table S22. Prevalence of PIK3CA genomic alterations in TCGA breast cancer dataset (n=809)

PAM50 subtype	Mutation (%)	Amplification (%)[a]	No. of Patients	P value
Basal-like	6.87	63.36	131	<0.0001
HER2	31.82	42.42	66	0.28
Luminal A	43.21	19.75	405	<0.0001
Luminal B	30.27	41.08	185	0.04
Normal-like	22.73	18.18	22	1.0
Total	32.96	33.50	809	

a . The prevalence includes both low level gain and high level amplification derived from the copy-number analysis algorithms GISTIC. The P value was calculated using X^2 test.

A 3q gene signature associated with triple negative breast cancer organ specific metastasis and response to neoadjuvant chemotherapy

Jun Qian[1] and Heidi Chen[2]

[1]Division of Pulmonary and Critical Care Medicine [2]Vanderbilt Center for Quantitative Sciences, Vanderbilt University Medical Center, Nashivlle,TN,USA

Contents

1 Figure 1 **3**

2 Figure S1 **5**

3 Figure S2 **7**

4 Figure S3 **12**

5 Figure S4 **15**

6 Table S3 **17**
- 6.1 GSE1121 17
- 6.2 GSE2034 17
- 6.3 TRANSBIG 17

7 Table S4 **18**
- 7.1 GSE2034 18
- 7.2 GSE11121 18
- 7.3 TRANSBIG 18
- 7.4 GSE11121+TRANSBIG 19
- 7.5 GSE2034+GSE11121+TRANSBIG 19

8 Table S5 **21**

9 Table S6 **21**

10 Table S7 **21**

11 univariable analysis : Table S8 23
11.1 Basal 23
11.2 LumB 23
11.3 LumA 23
11.4 HER2 24
11.5 Normal 24

12 Table S9 24
12.1 Basal 24
12.2 LumB 25
12.3 Her2 25
12.4 Normal 25

13 Figure 2 : KM plot for Basal-like 27

14 Figure 2 : KM plot for LumB 28

15 Table S11 30
15.1 Lung 30
15.1.1 EMC344 30
15.1.2 gse12276 30
15.1.3 gse2603 30
15.1.4 Combine 31
15.2 Brain 31
15.2.1 EMC344 31
15.2.2 gse12276 31
15.2.3 gse2603 31
15.2.4 Combine 32
15.3 Bone 32
15.3.1 EMC344 32
15.3.2 gse12276 32
15.3.3 gse2603 33
15.3.4 Combine 33

16 Table S12 34
16.0.5 Lung 34
16.1 Brain 34

17 Table 2 UniCox model for lung metastasis 35
17.1 Lung met in Basal-like 35
17.2 Lung met in Her2 35
17.3 Lung met in LumA 35
17.4 Lung met in LumB 36

17.5 Lung met in Normal . . . 36
17.6 Lung met in TNBC . . . 36
17.7 Lung met in Non-TNBC . . . 37

18 Table 2 multiCox model for lung metastasis **38**
18.1 see Table S13 for lung met in TNBC, non-TNBC and Basal-like . . . 38
18.2 Lung met in Her2 . . . 38
18.3 Lung met in LumA . . . 38

19 Table 2 UniCox model for brain metastasis **39**
19.1 brain met in Basal-like . . . 39
19.2 brain met in Her2 . . . 39
19.3 brain met in LumA . . . 39
19.4 brain met in LumB . . . 40
19.5 brain met in Normal . . . 40
19.6 brain met in TNBC . . . 40
19.7 brain met in Non-TNBC . . . 41

20 Table 2 multiCox model for brain metastasis **42**
20.1 see Table S14 for brain met in TNBC, non-TNBC and Basal-like . . . 42
20.2 Lung met in LumB . . . 42

21 Table S13 **43**
21.1 Lung TNBC . . . 43
21.2 Lung Non-TNBC . . . 43
21.3 Lung Basal-like . . . 43

22 Table S14 **45**
22.1 Brain TNBC . . . 45
22.2 Brain Non-TNBC . . . 45
22.3 Brain Basal-like . . . 45

23 Table S16 **46**

24 Table S17 **47**
24.1 TNBC . . . 47
24.2 non-TNBC . . . 47

25 Table S18 **48**
25.1 TNBC . . . 48
25.2 Non-TNBC . . . 49

26 Table S19 lung metastasis **52**
26.1 TNBC 52
26.2 non-TNBC 57

27 Table S19 brain metastasis **64**
27.1 TNBC 64
27.2 non-TNBC 67

28 Table S22 **72**

```r
library(meta)
library(rms)
library(xtable)
library(gridExtra)

source("C:/Users/qianj/OneDrive/temp8/paper/to ppm 08262016 3rd version/TO Nature SR 10112016/response 12202016/from Heidi/sweav file and dat

###### read in data

all <- read.csv("C:/Users/qianj/OneDrive/temp8/paper/to ppm 08262016 3rd version/TO Nature SR 10112016/response 12202016/from Heidi/sweav fil

all <- upData(all, levels = list(pam50.robust.1=list(Normal="Normal",
                                                      Basal="Basal",  Her2="Her2",  LumA="LumA", LumB="LumB")))
```

1 Figure 1

```
  ww <- kruskal.test(ch3q20.19.n.scale ~ pam50.robust.1, data=all)
if( ww$p.value < 0.001) tittxt="P < 0.001"  else
  tittxt <- paste0("P= ", round(ww$p.value,3))
  g1 <- ggplot(all,aes(y=ch3q20.19.n.scale,x=pam50.robust.1))+geom_boxplot()+ggtitle(tittxt)
  g1
```

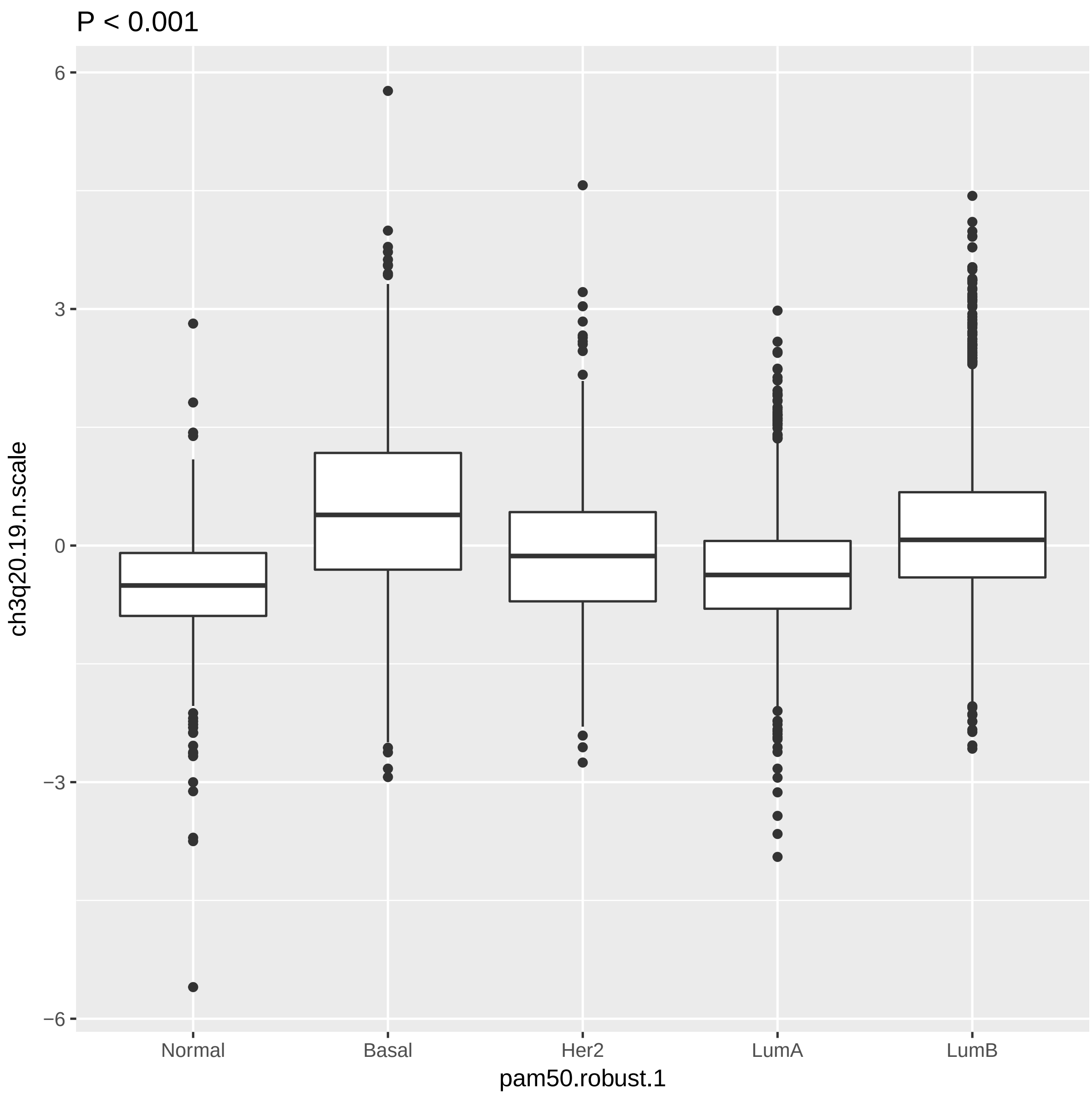
P < 0.001
6
3
0
−3
−6
ch3q20.19.n.scale
Normal
Basal
Her2
LumA
LumB
pam50.robust.1

2 Figure S1

```
selgroup <- c("age", "grade", "size","node","er.orig", "pr.orig", "her2.orig", "TRNeg")
pp=list()

for ( i in 1:length(selgroup) ) {

  temp <- subset(all, select=c("ch3q20.19.n.scale", selgroup[i]))
  colnames(temp) <- c("ch3q20.19.n.scale", "group")
  ww <- wilcox.test(ch3q20.19.n.scale ~ group, data=temp)
 if( ww$p.value < 0.001) tittxt="P < 0.001"  else
  tittxt <- paste0("P= ", round(ww$p.value,3))

  g1 <- ggplot(temp,aes(y=ch3q20.19.n.scale,x=group))+geom_boxplot()+xlab(selgroup[i])+ggtitle(tittxt)
  pp[[i]] <- g1

}


grid.arrange(pp[[1]],pp[[2]],pp[[3]], pp[[4]], pp[[5]],pp[[6]], pp[[7]], pp[[8]], ncol=3)
```

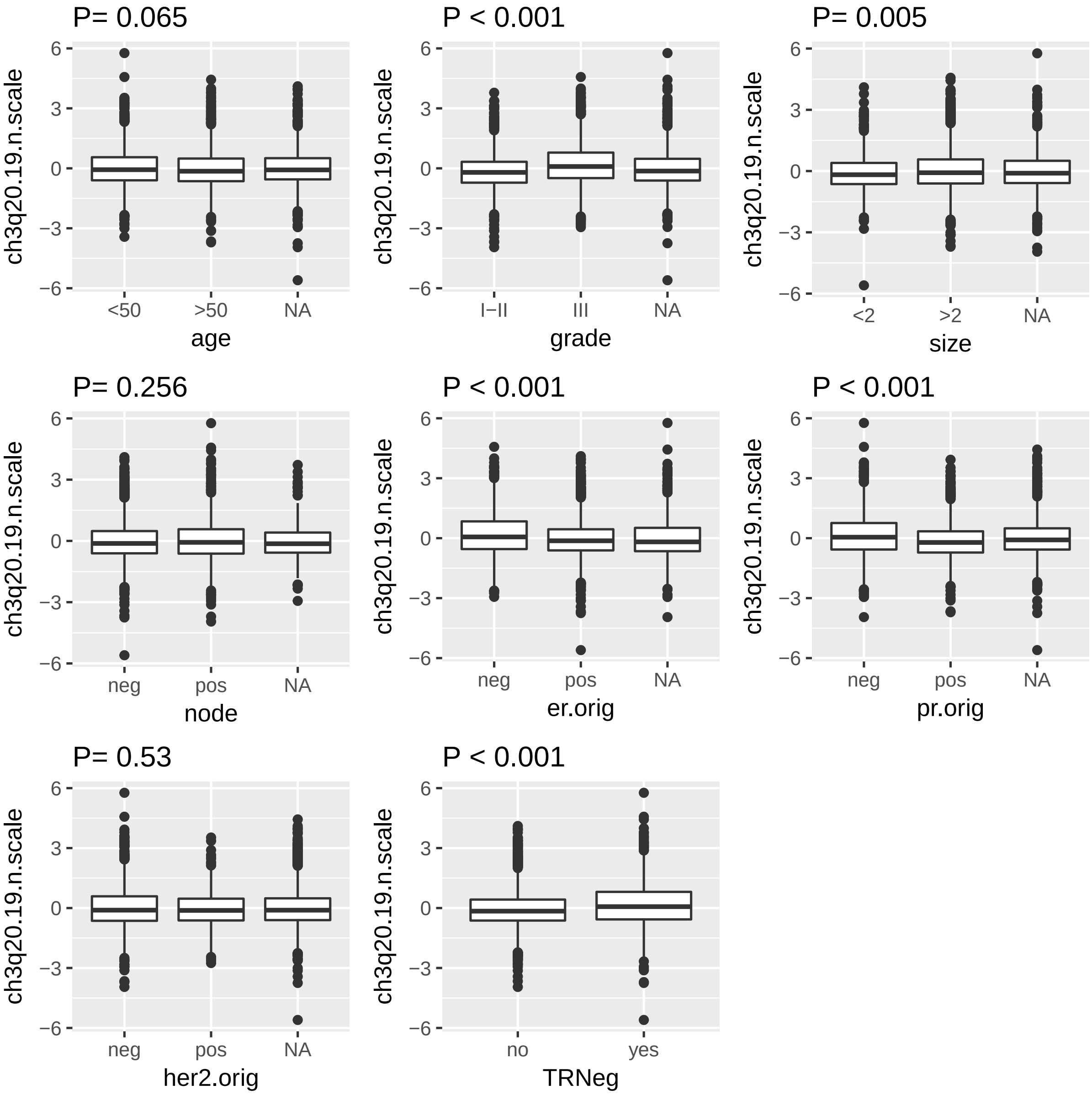
P= 0.065
P < 0.001
P= 0.005
P= 0.256
P < 0.001
P < 0.001
P= 0.53
P < 0.001
ch3q20.19.n.scale
6
3
0
−3
−6
<50
>50
NA
age
I−II
III
NA
grade
<2
>2
NA
size
neg
pos
NA
node
er.orig
pr.orig
her2.orig
no
yes
TRNeg

3 Figure S2

```
temp <- all
temp$dataset5 <- temp$dataset4
temp$dataset5 <-ifelse(temp$tbig302  %nin% c("tbig302"), temp$dataset5, "TRANSBIG")
temp$dataset3 <- temp$dataset5
temp <- subset(temp, dataset3 != "gse7390" )

temp <- subset(temp, dataset3 %in%  Cs(gse11121,
gse2034,
TRANSBIG,
gse2603,
gse12276,
gse5327,
gse12093,
gse17705,
gse19615,
gse26971,
gse9195,
etabm158,
gse1456,
gse20685,
gse25066,
gse16446)
)


temp$dataset3 <-factor(temp$dataset3, levels=Cs(gse11121,
gse2034,
TRANSBIG,
gse2603,
gse12276,
gse5327,
gse12093,
gse17705,
gse19615,
gse26971,
gse9195,
etabm158,
gse1456,
gse20685,
gse25066,
```

```
gse16446)
)
```

```r
library(knitr)
knitr::opts_chunk$set(echo=F, eval=T, message = F, warning=F, cache = F, fig=TRUE)
```

```
##
##  ############################## Meta analysis
##              HR           95%-CI %W(fixed) %W(random)
## gse11121 1.8452 [1.4256; 2.3882]       6.1        7.3
## gse2034  1.4476 [1.2135; 1.7269]      13.0        9.8
## TRANSBIG 1.2619 [1.0213; 1.5592]       9.0        8.6
## gse2603  1.3344 [0.8802; 2.0229]       2.3        4.1
## gse12276 1.2544 [1.0766; 1.4614]      17.3       10.6
## gse5327  1.0564 [0.6248; 1.7861]       1.5        2.9
## gse12093 1.9701 [1.1846; 3.2764]       1.6        3.0
## gse17705 1.4743 [1.1410; 1.9049]       6.2        7.3
## gse19615 1.6517 [0.9713; 2.8088]       1.4        2.8
## gse26971 1.1091 [0.8535; 1.4413]       5.9        7.2
## gse9195  1.2318 [0.7062; 2.1485]       1.3        2.6
## etabm158 0.9326 [0.6696; 1.2989]       3.7        5.5
## gse1456  0.8186 [0.5520; 1.2140]       2.6        4.4
## gse20685 1.1016 [0.9062; 1.3391]      10.6        9.1
## gse25066 1.0949 [0.9286; 1.2908]      14.9       10.2
## gse16446 1.0289 [0.6982; 1.5161]       2.7        4.5
##
## Number of studies combined: k = 16
##
##                          HR           95%-CI    z  p-value
## Fixed effect model   1.2463 [1.1696; 1.3281] 6.79 < 0.0001
## Random effects model 1.2471 [1.1282; 1.3787] 4.32 < 0.0001
##
## Quantifying heterogeneity:
##  tau^2 = 0.0187; H = 1.44 [1.08; 1.91]; I^2 = 51.5% [14.1%; 72.6%]
##
## Test of heterogeneity:
##      Q d.f.  p-value
##  30.93   15   0.0090
##
## Details on meta-analytical method:
```

```
## - Inverse variance method
## - DerSimonian-Laird estimator for tau^2
```

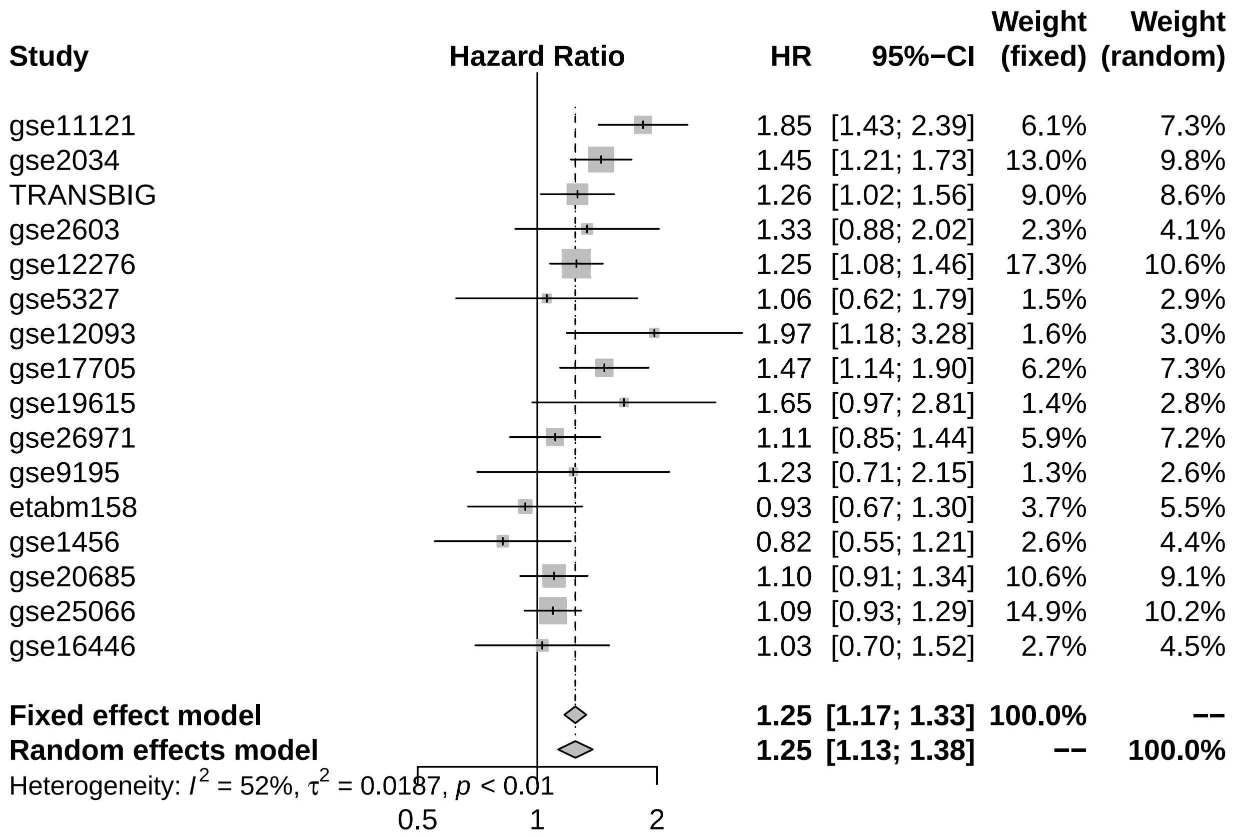
Study
Hazard Ratio
HR
95%−CI
Weight (fixed)
Weight (random)
gse11121 1.85 [1.43; 2.39] 6.1% 7.3%
gse2034 1.45 [1.21; 1.73] 13.0% 9.8%
TRANSBIG 1.26 [1.02; 1.56] 9.0% 8.6%
gse2603 1.33 [0.88; 2.02] 2.3% 4.1%
gse12276 1.25 [1.08; 1.46] 17.3% 10.6%
gse5327 1.06 [0.62; 1.79] 1.5% 2.9%
gse12093 1.97 [1.18; 3.28] 1.6% 3.0%
gse17705 1.47 [1.14; 1.90] 6.2% 7.3%
gse19615 1.65 [0.97; 2.81] 1.4% 2.8%
gse26971 1.11 [0.85; 1.44] 5.9% 7.2%
gse9195 1.23 [0.71; 2.15] 1.3% 2.6%
etabm158 0.93 [0.67; 1.30] 3.7% 5.5%
gse1456 0.82 [0.55; 1.21] 2.6% 4.4%
gse20685 1.10 [0.91; 1.34] 10.6% 9.1%
gse25066 1.09 [0.93; 1.29] 14.9% 10.2%
gse16446 1.03 [0.70; 1.52] 2.7% 4.5%
Fixed effect model 1.25 [1.17; 1.33] 100.0% --
Random effects model 1.25 [1.13; 1.38] -- 100.0%
Heterogeneity: I^2 = 52%, τ^2 = 0.0187, p < 0.01
0.5
1
2

```
##
##
##  ################################### Adjust for gse cluster
##                    exp(coef) exp(-coef) lower .95 upper .95 pvalue    se
## ch3q20.19.n.scale     1.182      0.846     1.079     1.296      0 0.032
##                    rob.se
## ch3q20.19.n.scale  0.047
```

4 Figure S3

```
##
##  ############################### Meta analysis
##                HR           95%-CI %W(fixed) %W(random)
## etabm158 0.9692 [0.7354; 1.2774]       9.3        9.7
## gse16391 2.1013 [1.1472; 3.8488]       1.9        3.7
## gse20685 0.7577 [0.4879; 1.1768]       3.7        5.8
## gse20711 0.8451 [0.6150; 1.1612]       7.0        8.5
## gse21653 1.2797 [0.9915; 1.6517]      10.9       10.3
## gse31519 0.9204 [0.6347; 1.3346]       5.1        7.2
## gse3494  1.2635 [1.0176; 1.5689]      15.1       11.6
## gse42568 0.9292 [0.6892; 1.2529]       7.9        9.0
## gse45255 1.4467 [0.9320; 2.2456]       3.7        5.9
## gse6532  1.4554 [1.1850; 1.7876]      16.8       11.9
## gse7390  1.0765 [0.8725; 1.3281]      16.1       11.8
## gse9195  0.9746 [0.5767; 1.6471]       2.6        4.6
##
## Number of studies combined: k = 12
##
##                          HR           95%-CI    z  p-value
## Fixed effect model   1.1380 [1.0461; 1.2379] 3.01   0.0026
## Random effects model 1.1138 [0.9764; 1.2706] 1.60   0.1087
##
## Quantifying heterogeneity:
##  tau^2 = 0.0268; H = 1.47 [1.06; 2.04]; I^2 = 53.9% [11.5%; 76.0%]
##
## Test of heterogeneity:
##      Q d.f.  p-value
##  23.87   11   0.0133
##
## Details on meta-analytical method:
## - Inverse variance method
## - DerSimonian-Laird estimator for tau^2
```

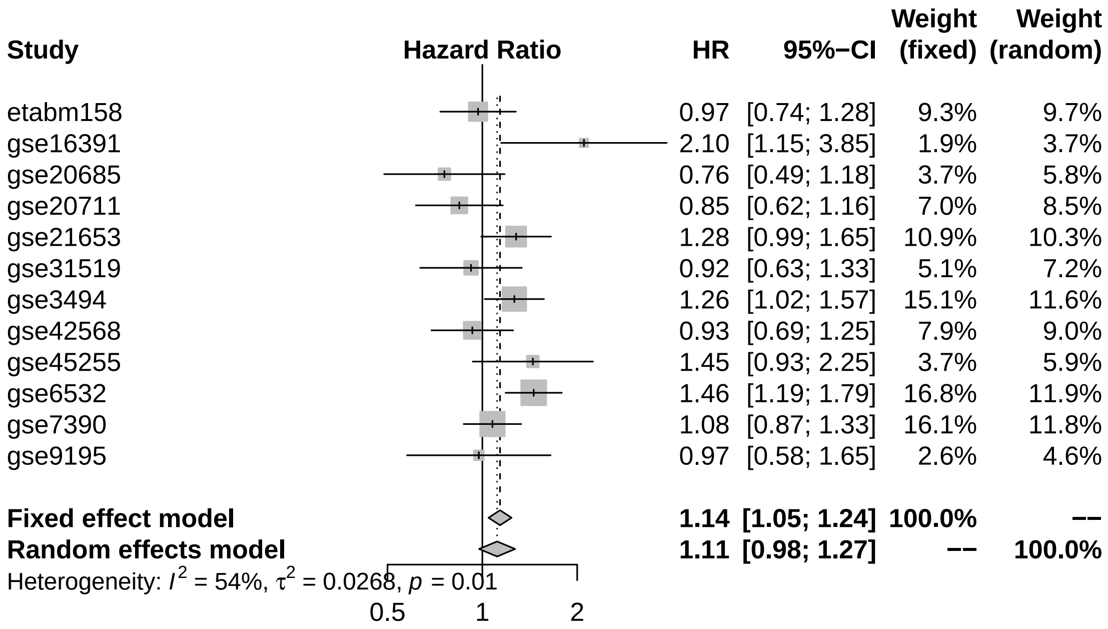
Study
Hazard Ratio
HR
95%−CI
Weight (fixed)
Weight (random)
etabm158 0.97 [0.74; 1.28] 9.3% 9.7%
gse16391 2.10 [1.15; 3.85] 1.9% 3.7%
gse20685 0.76 [0.49; 1.18] 3.7% 5.8%
gse20711 0.85 [0.62; 1.16] 7.0% 8.5%
gse21653 1.28 [0.99; 1.65] 10.9% 10.3%
gse31519 0.92 [0.63; 1.33] 5.1% 7.2%
gse3494 1.26 [1.02; 1.57] 15.1% 11.6%
gse42568 0.93 [0.69; 1.25] 7.9% 9.0%
gse45255 1.45 [0.93; 2.25] 3.7% 5.9%
gse6532 1.46 [1.19; 1.79] 16.8% 11.9%
gse7390 1.08 [0.87; 1.33] 16.1% 11.8%
gse9195 0.97 [0.58; 1.65] 2.6% 4.6%
Fixed effect model 1.14 [1.05; 1.24] 100.0% −−
Random effects model 1.11 [0.98; 1.27] −− 100.0%
Heterogeneity: I² = 54%, τ² = 0.0268, p = 0.01
0.5
1
2

```
##
##
```

```
##  ################################### Adjust for gse cluster
##                    exp(coef) exp(-coef) lower .95 upper .95 pvalue    se
## ch3q20.19.n.scale     1.102      0.907     0.993     1.222  0.066 0.042
##                    rob.se
## ch3q20.19.n.scale  0.053
```

5 Figure S4

```
temp788 <- subset(all, dataset4 %in% c("gse11121","gse2034" ) | tbig302 %in% c("tbig302","gse2034"))  ### 788
temp788$dataset3 <- ifelse(temp788$dataset4 %in% c("gse11121","gse2034" ) , temp788$dataset4, "transbig" )
temp <- temp788
temp$dataset3 <- factor(temp$dataset3, levels=c("gse2034", "gse11121", "transbig"))

  ww <- kruskal.test(ch3q20.19.n.scale ~ dataset3, data=temp)
if( ww$p.value < 0.001) tittxt="P < 0.001"  else
  tittxt <- paste0("P= ", round(ww$p.value,3))
  g1 <- ggplot(temp,aes(y=ch3q20.19.n.scale,x=dataset3))+geom_boxplot()+ggtitle(tittxt)
  g1
```

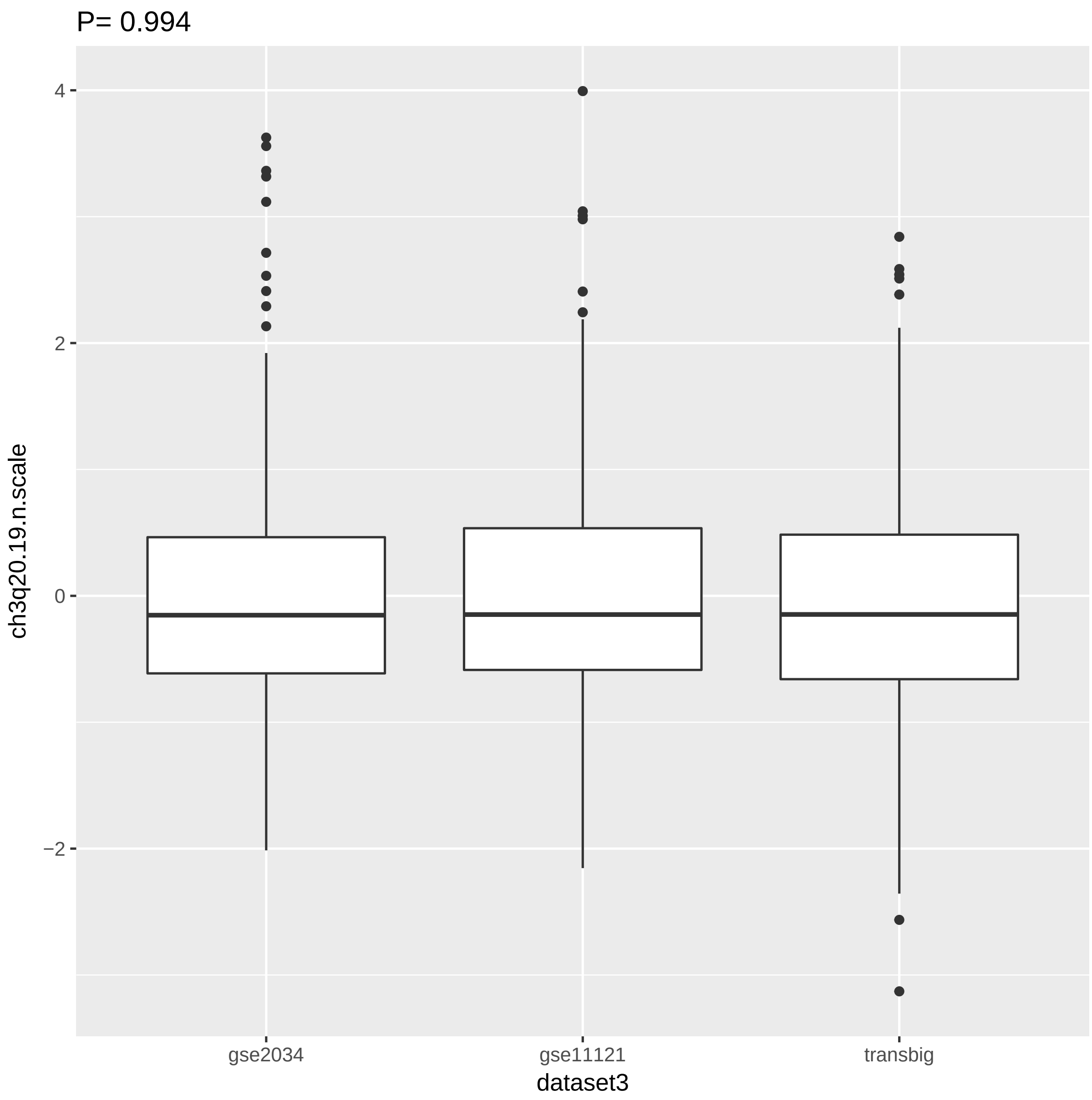
P= 0.994
4
2
0
−2
ch3q20.19.n.scale
gse2034
gse11121
transbig
dataset3

6 Table S3

6.1 GSE1121

```
temp <- subset(temp788, dataset3=="gse11121")
get_analysis(datain=temp, outcome="DMFS",xin="ch3q20.19.n.scale", metflag="no")


   ################################### Not Adjust for gse cluster ( one gse)
                  exp(coef) exp(-coef) lower .95 upper .95 pvalue
ch3q20.19.n.scale     1.845      0.542     1.426     2.388      0
```

6.2 GSE2034

```
temp <- subset(temp788, dataset3=="gse2034")
get_analysis(datain=temp, outcome="DMFS",xin="ch3q20.19.n.scale", metflag="no")


   ################################### Not Adjust for gse cluster ( one gse)
                  exp(coef) exp(-coef) lower .95 upper .95 pvalue
ch3q20.19.n.scale     1.448      0.691     1.213     1.727      0
```

6.3 TRANSBIG

```
temp <- subset(temp788, dataset3=="transbig")
get_analysis(datain=temp, outcome="DMFS",xin="ch3q20.19.n.scale", metflag="no")


   ################################### Not Adjust for gse cluster ( one gse)
                  exp(coef) exp(-coef) lower .95 upper .95 pvalue
ch3q20.19.n.scale     1.262      0.792     1.021     1.559  0.031
```

7 Table S4

7.1 GSE2034

```
temp <- subset(temp788, dataset3=="gse2034")

get_analysis(datain=temp, outcome="DMFS",xin="ch3q20.19.n.scale+er+pr+her2+grade+age", metflag="no")


   #################################### Not Adjust for gse cluster ( one gse)
                  exp(coef) lower .95 upper .95 pvalue
ch3q20.19.n.scale     1.382     1.109     1.721  0.004
erpos                 1.410     0.823     2.417  0.211
prpos                 0.544     0.298     0.991  0.047
her2pos               0.791     0.412     1.519  0.481
gradeIII              2.261     1.140     4.482  0.020
age>50                0.810     0.497     1.323  0.400
```

7.2 GSE11121

```
temp <- subset(temp788, dataset3=="gse11121")

get_analysis(datain=temp, outcome="DMFS",xin="ch3q20.19.n.scale+er+pr+her2+grade+age+size", metflag="no")


   #################################### Not Adjust for gse cluster ( one gse)
                  exp(coef) lower .95 upper .95 pvalue
ch3q20.19.n.scale     1.825     1.405     2.370  0.000
erpos                 0.670     0.332     1.351  0.263
prpos                 1.258     0.589     2.685  0.554
her2pos               1.839     0.871     3.879  0.110
gradeIII              1.567     0.731     3.357  0.248
age>50                1.402     0.698     2.816  0.343
size>2                0.936     0.490     1.789  0.841
```

7.3 TRANSBIG

```
temp <- subset(temp788, dataset3=="transbig")

get_analysis(datain=temp, outcome="DMFS",xin="ch3q20.19.n.scale+er+pr+her2+grade+age+size", metflag="no")


   ################################### Not Adjust for gse cluster ( one gse)
                  exp(coef) lower .95 upper .95 pvalue
ch3q20.19.n.scale     1.184     0.954     1.470  0.125
erpos                 0.701     0.423     1.160  0.167
prpos                 0.773     0.443     1.350  0.366
her2pos               0.913     0.508     1.642  0.761
gradeIII              0.740     0.436     1.254  0.263
age>50                1.125     0.705     1.796  0.620
size>2                3.120     1.747     5.570  0.000
```

7.4 GSE11121+TRANSBIG

```
temp <- subset(temp788, dataset3 %in% c("transbig", "gse11121"))
get_analysis(datain=temp, outcome="DMFS",xin="ch3q20.19.n.scale+er+pr+her2+grade+age+size", metflag="no")



 ################################### Adjust for gse cluster
                  exp(coef) lower .95 upper .95 pvalue    se rob.se
ch3q20.19.n.scale     1.361     1.038     1.783  0.026 0.085  0.138
erpos                 0.685     0.644     0.729  0.000 0.204  0.032
prpos                 0.847     0.657     1.092  0.199 0.226  0.130
her2pos               1.161     0.750     1.796  0.504 0.229  0.223
gradeIII              0.919     0.602     1.403  0.697 0.220  0.216
age>50                1.115     1.023     1.215  0.013 0.188  0.044
size>2                1.894     0.880     4.075  0.102 0.205  0.391
```

7.5 GSE2034+GSE11121+TRANSBIG

```
get_analysis(datain=temp788, outcome="DMFS",xin="ch3q20.19.n.scale+er+pr+her2+grade+age", metflag="no")



 ################################### Adjust for gse cluster
```

```
                  exp(coef) lower .95 upper .95 pvalue    se rob.se
ch3q20.19.n.scale     1.355     1.162     1.580  0.000 0.067  0.078
erpos                 0.954     0.651     1.399  0.810 0.160  0.195
prpos                 0.763     0.600     0.971  0.028 0.178  0.123
her2pos               0.957     0.717     1.278  0.767 0.183  0.147
gradeIII              1.537     0.937     2.523  0.089 0.152  0.253
age>50                1.095     0.927     1.293  0.285 0.146  0.085
```

8 Table S5

```
get_analysis(datain=temp788, outcome="DMFS",xin="ch3q20.19.n.scale+pam50.robust.1", metflag="no")
```

```
 #################################### Adjust for gse cluster
                   exp(coef) lower .95 upper .95 pvalue    se rob.se
ch3q20.19.n.scale       1.380     1.227     1.553  0.000 0.066  0.060
pam50.robust.1Basal     1.758     1.268     2.438  0.001 0.476  0.167
pam50.robust.1Her2      1.952     1.227     3.105  0.005 0.487  0.237
pam50.robust.1LumA      1.092     0.737     1.618  0.662 0.466  0.201
pam50.robust.1LumB      2.252     1.539     3.296  0.000 0.460  0.194
```

9 Table S6

```
get_analysis(datain=temp788, outcome="DMFS",xin="ch3q20.19.n.scale+GENE70+GENE76+GGI+oncotypedx2+pcna117.n.scale", metflag="no")
```

```
 #################################### Adjust for gse cluster
                 exp(coef) lower .95 upper .95 pvalue    se rob.se
ch3q20.19.n.scale     1.243     1.140     1.355  0.000 0.069  0.044
GENE70                1.618     0.505     5.182  0.418 0.714  0.594
GENE76                1.011     1.006     1.017  0.000 0.002  0.003
GGI                   2.040     0.233    17.898  0.520 0.816  1.108
oncotypedx2           1.000     0.995     1.006  0.891 0.005  0.003
pcna117.n.scale       0.661     0.263     1.660  0.379 0.367  0.470
```

10 Table S7

```
get_analysis(datain=temp788, outcome="DMFS",xin="ch3q20.19.n.scale+tcell.g11121.scale+bcell.g11121.scale+prof.g11121.scale+er.g11121.scale",
```

```
 #################################### Adjust for gse cluster
                  exp(coef) lower .95 upper .95 pvalue    se rob.se
```

```
ch3q20.19.n.scale       1.258     1.166     1.357  0.000 0.068  0.039
tcell.g11121.scale      0.945     0.902     0.990  0.017 0.090  0.024
bcell.g11121.scale      0.705     0.667     0.746  0.000 0.096  0.029
prof.g11121.scale       1.224     1.193     1.256  0.000 0.088  0.013
er.g11121.scale         0.883     0.812     0.960  0.003 0.087  0.043
```

11 univariable analysis : Table S8

11.1 Basal

```
temp <- subset(temp788, pam50.robust.1== "Basal")
get_analysis(datain=temp, outcome="DMFS",xin="ch3q20.19.n.scale", metflag="no")



 ################################### Adjust for gse cluster
                  exp(coef) exp(-coef) lower .95 upper .95 pvalue    se
ch3q20.19.n.scale     1.274      0.785     1.186     1.368      0 0.112
                  rob.se
ch3q20.19.n.scale  0.037
```

11.2 LumB

```
temp <- subset(temp788, pam50.robust.1== "LumB")
get_analysis(datain=temp, outcome="DMFS",xin="ch3q20.19.n.scale", metflag="no")



 ################################### Adjust for gse cluster
                  exp(coef) exp(-coef) lower .95 upper .95 pvalue    se
ch3q20.19.n.scale     1.662      0.602     1.531     1.804      0 0.107
                  rob.se
ch3q20.19.n.scale  0.042
```

11.3 LumA

```
temp <- subset(temp788, pam50.robust.1== "LumA")
get_analysis(datain=temp, outcome="DMFS",xin="ch3q20.19.n.scale", metflag="no")



 ################################### Adjust for gse cluster
                  exp(coef) exp(-coef) lower .95 upper .95 pvalue    se
ch3q20.19.n.scale     1.018      0.982     0.653     1.588  0.936 0.168
                  rob.se
ch3q20.19.n.scale  0.227
```

11.4 HER2

```
temp <- subset(temp788, pam50.robust.1== "Her2")
get_analysis(datain=temp, outcome="DMFS",xin="ch3q20.19.n.scale", metflag="no")
```

```
 ################################### Adjust for gse cluster
                 exp(coef) exp(-coef) lower .95 upper .95 pvalue    se
ch3q20.19.n.scale     1.399      0.715      1.01     1.939  0.043 0.206
                 rob.se
ch3q20.19.n.scale  0.166
```

11.5 Normal

```
temp <- subset(temp788, pam50.robust.1== "Normal")
get_analysis(datain=temp, outcome="DMFS",xin="ch3q20.19.n.scale", metflag="no")
```

```
 ################################### Adjust for gse cluster
                 exp(coef) exp(-coef) lower .95 upper .95 pvalue    se
ch3q20.19.n.scale     2.262      0.442     1.296     3.948  0.004 0.735
                 rob.se
ch3q20.19.n.scale  0.284
```

12 Table S9

12.1 Basal

```
temp=subset(temp788, pam50.robust.1=="Basal" )
get_analysis(datain=temp, outcome="DMFS",xin="ch3q20.19.n.scale+bcell.g11121.scale+prof.g11121.scale", metflag="no")
```

```
 ################################### Adjust for gse cluster
                  exp(coef) lower .95 upper .95 pvalue    se rob.se
ch3q20.19.n.scale      1.486     1.349     1.636  0.000 0.126  0.049
```

```
bcell.g11121.scale     0.632     0.597     0.669  0.000 0.147  0.029
prof.g11121.scale      0.585     0.424     0.805  0.001 0.202  0.163
```

12.2 LumB

```
temp=subset(temp788, pam50.robust.1=="LumB" )

get_analysis(datain=temp, outcome="DMFS",xin="ch3q20.19.n.scale+bcell.g11121.scale+prof.g11121.scale", metflag="no")



 ################################### Adjust for gse cluster
                   exp(coef) lower .95 upper .95 pvalue    se rob.se
ch3q20.19.n.scale      1.406     1.240     1.594  0.000 0.122  0.064
bcell.g11121.scale     0.737     0.644     0.844  0.000 0.111  0.069
prof.g11121.scale      1.517     1.084     2.124  0.015 0.149  0.172
```

12.3 Her2

```
temp=subset(temp788, pam50.robust.1=="Her2" )

get_analysis(datain=temp, outcome="DMFS",xin="ch3q20.19.n.scale+bcell.g11121.scale+prof.g11121.scale", metflag="no")



 ################################### Adjust for gse cluster
                   exp(coef) lower .95 upper .95 pvalue    se rob.se
ch3q20.19.n.scale      1.263     0.838     1.904  0.265 0.203  0.209
bcell.g11121.scale     0.528     0.420     0.663  0.000 0.199  0.116
prof.g11121.scale      0.566     0.287     1.116  0.100 0.319  0.347
```

12.4 Normal

```
temp=subset(temp788, pam50.robust.1=="Normal" )

get_analysis(datain=temp, outcome="DMFS",xin="ch3q20.19.n.scale+bcell.g11121.scale+prof.g11121.scale", metflag="no")
```

```
################################### Adjust for gse cluster
                  exp(coef) lower .95 upper .95 pvalue    se rob.se
ch3q20.19.n.scale      3.374     0.354    32.152  0.290 1.260  1.150
bcell.g11121.scale     0.213     0.089     0.506  0.000 0.839  0.442
prof.g11121.scale     19.528     0.370  1030.472  0.142 1.871  2.023
```

13 Figure 2 : KM plot for Basal-like

```r
temp=subset(temp788, pam50.robust.1=="Basal" )

temp$ch3q.group3 <- ifelse(temp$ch3q20.19.n.scale < quantile(temp$ch3q20.19.n.scale, 0.33), "Low",
                          ifelse(temp$ch3q20.19.n.scale < quantile(temp$ch3q20.19.n.scale, 0.66), "Median", "High"))

get_KMplot(datain=temp, outcome="DMFS", group="ch3q.group3")
```

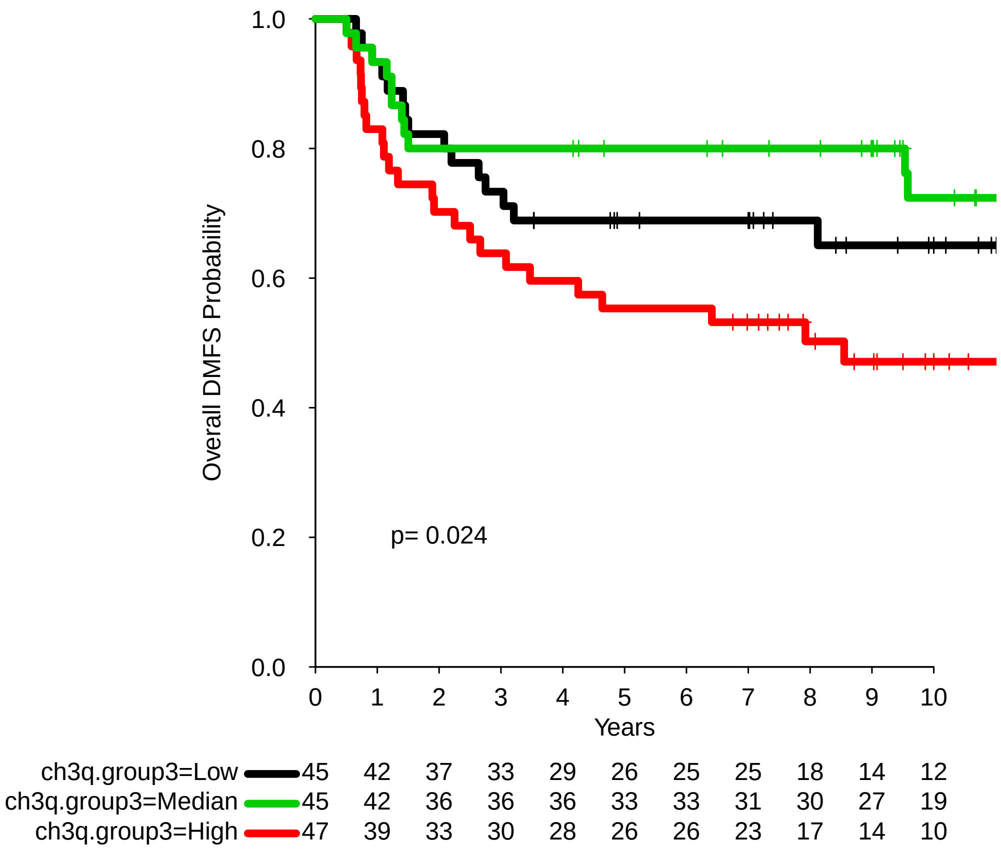

14 Figure 2 : KM plot for LumB

```
temp=subset(temp788, pam50.robust.1=="LumB" )
temp$ch3q.group3 <- ifelse(temp$ch3q20.19.n.scale < quantile(temp$ch3q20.19.n.scale, 0.33), "Low",
                          ifelse(temp$ch3q20.19.n.scale < quantile(temp$ch3q20.19.n.scale, 0.66), "Median", "High"))
get_KMplot(datain=temp, outcome="DMFS", group="ch3q.group3")
```

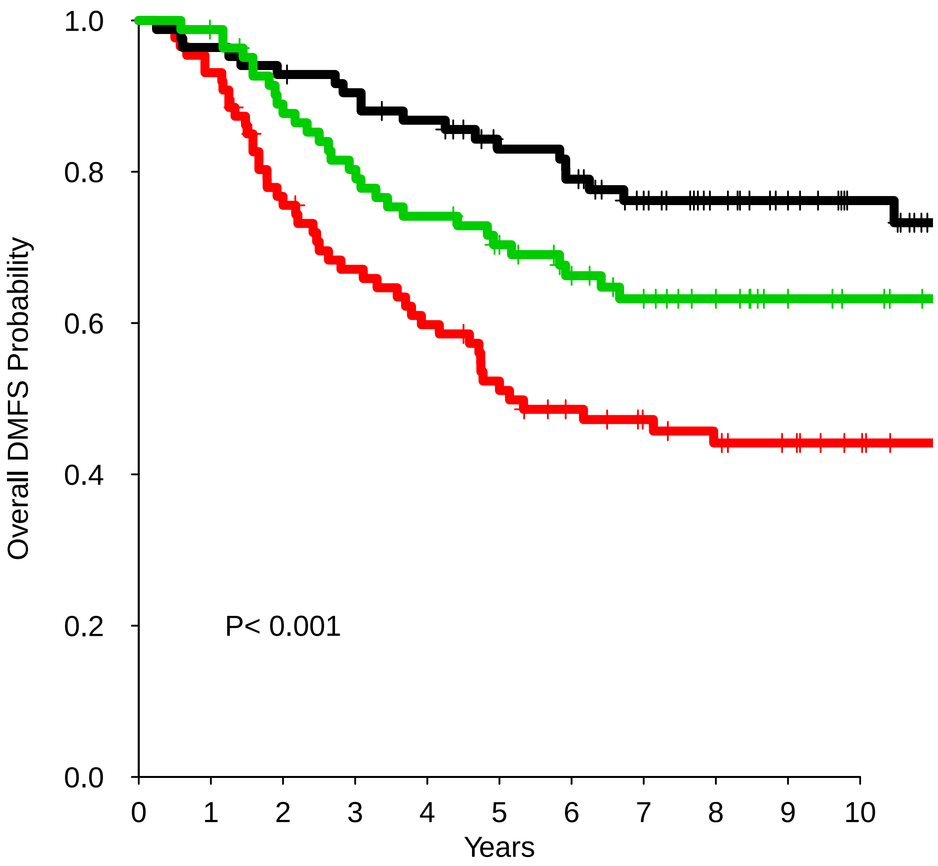

	0	1	2	3	4	5	6	7	8	9	10
ch3q.group3=Low	84	81	78	75	71	63	59	50	40	33	26
ch3q.group3=Median	83	81	72	65	60	55	46	41	35	29	25
ch3q.group3=High	87	81	65	55	49	42	36	31	28	25	21

```
temp618 <- subset(all, dataset3 %in% c("gse12276","EMC344", "gse2603" ))

temp.TNBC1 <- subset(temp618,  TRNeg=="yes")
temp.TNBC0 <- subset(temp618,  TRNeg=="no")
```

15 Table S11

15.1 Lung

15.1.1 EMC344

```
temp <- subset(temp618, dataset3=="EMC344")
get_analysis(datain=temp, outcome="DMFS.lung",xin="ch3q20.19.n.scale", metflag="no")


   ################################### Not Adjust for gse cluster ( one gse)
                  exp(coef) exp(-coef) lower .95 upper .95 pvalue
ch3q20.19.n.scale     1.683      0.594     1.243     2.278  0.001
```

15.1.2 gse12276

```
temp <- subset(temp618, dataset3=="gse12276")
get_analysis(datain=temp, outcome="DMFS.lung",xin="ch3q20.19.n.scale", metflag="no")


   ################################### Not Adjust for gse cluster ( one gse)
                  exp(coef) exp(-coef) lower .95 upper .95 pvalue
ch3q20.19.n.scale     1.881      0.532     1.424     2.484      0
```

15.1.3 gse2603

```
temp <- subset(temp618, dataset3=="gse2603")
get_analysis(datain=temp, outcome="DMFS.lung",xin="ch3q20.19.n.scale", metflag="no")


   ################################### Not Adjust for gse cluster ( one gse)
                  exp(coef) exp(-coef) lower .95 upper .95 pvalue
ch3q20.19.n.scale     2.352      0.425     1.333      4.15  0.003
```

15.1.4 Combine

```
get_analysis(datain=temp618, outcome="DMFS.lung",xin="ch3q20.19.n.scale", metflag="no")
```

```
 #################################### Adjust for gse cluster
                  exp(coef) exp(-coef) lower .95 upper .95 pvalue    se
ch3q20.19.n.scale     1.743      0.574     1.598       1.9      0 0.095
                  rob.se
ch3q20.19.n.scale  0.044
```

15.2 Brain

15.2.1 EMC344

```
temp <- subset(temp618, dataset3=="EMC344")
get_analysis(datain=temp, outcome="DMFS.brain",xin="ch3q20.19.n.scale", metflag="no")
```

```
   #################################### Not Adjust for gse cluster ( one gse)
                  exp(coef) exp(-coef) lower .95 upper .95 pvalue
ch3q20.19.n.scale     2.497      0.401     1.622     3.842      0
```

15.2.2 gse12276

```
temp <- subset(temp618, dataset3=="gse12276")
get_analysis(datain=temp, outcome="DMFS.brain",xin="ch3q20.19.n.scale", metflag="no")
```

```
   #################################### Not Adjust for gse cluster ( one gse)
                  exp(coef) exp(-coef) lower .95 upper .95 pvalue
ch3q20.19.n.scale     1.914      0.523     1.185     3.091  0.008
```

15.2.3 gse2603

```
temp <- subset(temp618, dataset3=="gse2603")
get_analysis(datain=temp, outcome="DMFS.brain",xin="ch3q20.19.n.scale", metflag="no")


   ################################### Not Adjust for gse cluster ( one gse)
                  exp(coef) exp(-coef) lower .95 upper .95 pvalue
ch3q20.19.n.scale     1.349      0.741     0.508     3.579  0.548
```

15.2.4 Combine

```
get_analysis(datain=temp618, outcome="DMFS.brain",xin="ch3q20.19.n.scale", metflag="no")



 ################################### Adjust for gse cluster
                  exp(coef) exp(-coef) lower .95 upper .95 pvalue    se
ch3q20.19.n.scale     1.993      0.502     1.623     2.448      0 0.153
                  rob.se
ch3q20.19.n.scale  0.105
```

15.3 Bone

15.3.1 EMC344

```
temp <- subset(temp618, dataset3=="EMC344")
get_analysis(datain=temp, outcome="DMFS.bone",xin="ch3q20.19.n.scale", metflag="no")


   ################################### Not Adjust for gse cluster ( one gse)
                  exp(coef) exp(-coef) lower .95 upper .95 pvalue
ch3q20.19.n.scale     1.338      0.748     1.062     1.684  0.013
```

15.3.2 gse12276

```
temp <- subset(temp618, dataset3=="gse12276")
get_analysis(datain=temp, outcome="DMFS.bone",xin="ch3q20.19.n.scale", metflag="no")
```

```
   #################################### Not Adjust for gse cluster ( one gse)
                  exp(coef) exp(-coef) lower .95 upper .95 pvalue
ch3q20.19.n.scale     0.998      1.002     0.808     1.234  0.988
```

15.3.3 gse2603

```
temp <- subset(temp618, dataset3=="gse2603")
get_analysis(datain=temp, outcome="DMFS.bone",xin="ch3q20.19.n.scale", metflag="no")


   #################################### Not Adjust for gse cluster ( one gse)
                  exp(coef) exp(-coef) lower .95 upper .95 pvalue
ch3q20.19.n.scale     0.763       1.31     0.416     1.401  0.383
```

15.3.4 Combine

```
get_analysis(datain=temp618, outcome="DMFS.bone",xin="ch3q20.19.n.scale", metflag="no")



 #################################### Adjust for gse cluster
                  exp(coef) exp(-coef) lower .95 upper .95 pvalue    se
ch3q20.19.n.scale     1.044      0.958      0.83     1.313  0.715 0.081
                  rob.se
ch3q20.19.n.scale  0.117
```

16 Table S12

16.0.5 Lung

```
get_analysis(datain=temp618, outcome="DMFS.lung",xin="ch3q20.19.n.scale+age+node+er+pr+her2", metflag="no")



 ################################### Adjust for gse cluster
                 exp(coef) lower .95 upper .95 pvalue    se rob.se
ch3q20.19.n.scale     1.584     1.424     1.761  0.000 0.105  0.054
age>50                1.189     0.820     1.723  0.360 0.236  0.189
nodepos               2.821     0.998     7.974  0.050 0.236  0.530
erpos                 0.485     0.185     1.274  0.142 0.280  0.492
prpos                 0.631     0.504     0.790  0.000 0.363  0.115
her2pos               0.748     0.367     1.525  0.424 0.312  0.363
```

16.1 Brain

```
get_analysis(datain=temp618, outcome="DMFS.brain",xin="ch3q20.19.n.scale+age+node+er+pr+her2", metflag="no")



 ################################### Adjust for gse cluster
                 exp(coef) lower .95 upper .95 pvalue    se rob.se
ch3q20.19.n.scale     1.605     1.210     2.130  0.001 0.169  0.144
age>50                0.982     0.430     2.240  0.965 0.390  0.421
nodepos               3.040     1.143     8.090  0.026 0.394  0.499
erpos                 0.386     0.311     0.478  0.000 0.487  0.110
prpos                 0.298     0.064     1.382  0.122 0.802  0.782
her2pos               0.596     0.465     0.765  0.000 0.553  0.127
```

17 Table 2 UniCox model for lung metastasis

17.1 Lung met in Basal-like

```
temp=subset(temp618, pam50.robust.1=="Basal" )
get_analysis(datain=temp, outcome="DMFS.lung",xin="ch3q20.19.n.scale", metflag="no")



 ################################### Adjust for gse cluster
                  exp(coef) exp(-coef) lower .95 upper .95 pvalue    se
ch3q20.19.n.scale     1.366      0.732     1.277     1.462      0 0.132
                  rob.se
ch3q20.19.n.scale  0.035
```

17.2 Lung met in Her2

```
temp=subset(temp618, pam50.robust.1=="Her2" )
get_analysis(datain=temp, outcome="DMFS.lung",xin="ch3q20.19.n.scale", metflag="no")



 ################################### Adjust for gse cluster
                  exp(coef) exp(-coef) lower .95 upper .95 pvalue    se
ch3q20.19.n.scale     3.292      0.304     1.385     7.822  0.007 0.533
                  rob.se
ch3q20.19.n.scale  0.442
```

17.3 Lung met in LumA

```
temp=subset(temp618, pam50.robust.1=="LumA" )
get_analysis(datain=temp, outcome="DMFS.lung",xin="ch3q20.19.n.scale", metflag="no")



 ################################### Adjust for gse cluster
                  exp(coef) exp(-coef) lower .95 upper .95 pvalue    se
ch3q20.19.n.scale     1.884      0.531     1.171     3.032  0.009 0.389
                  rob.se
ch3q20.19.n.scale  0.243
```

17.4 Lung met in LumB

```
temp=subset(temp618, pam50.robust.1=="LumB" )
get_analysis(datain=temp, outcome="DMFS.lung",xin="ch3q20.19.n.scale", metflag="no")



 ################################### Adjust for gse cluster
                  exp(coef) exp(-coef) lower .95 upper .95 pvalue    se
ch3q20.19.n.scale     1.289      0.776     0.707     2.349  0.407 0.267
                  rob.se
ch3q20.19.n.scale  0.306
```

17.5 Lung met in Normal

```
temp=subset(temp618, pam50.robust.1=="Normal" )
get_analysis(datain=temp, outcome="DMFS.lung",xin="ch3q20.19.n.scale", metflag="no")



 ################################### Adjust for gse cluster
                  exp(coef) exp(-coef) lower .95 upper .95 pvalue    se
ch3q20.19.n.scale      1.08      0.926     0.822     1.419  0.579 0.589
                  rob.se
ch3q20.19.n.scale  0.139
```

17.6 Lung met in TNBC

```
get_analysis(datain=temp.TNBC1, outcome="DMFS.lung",xin="ch3q20.19.n.scale", metflag="no")



 ################################### Adjust for gse cluster
                  exp(coef) exp(-coef) lower .95 upper .95 pvalue    se
ch3q20.19.n.scale     1.389       0.72     1.335     1.445      0 0.121
                  rob.se
ch3q20.19.n.scale   0.02
```

17.7 Lung met in Non-TNBC

```
get_analysis(datain=temp.TNBC0, outcome="DMFS.lung",xin="ch3q20.19.n.scale", metflag="no")



 ################################### Adjust for gse cluster
                  exp(coef) exp(-coef) lower .95 upper .95 pvalue    se
ch3q20.19.n.scale     1.869      0.535       1.4     2.494      0 0.195
                  rob.se
ch3q20.19.n.scale  0.147
```

18 Table 2 multiCox model for lung metastasis

18.1 see Table S13 for lung met in TNBC, non-TNBC and Basal-like

18.2 Lung met in Her2

```
temp=subset(temp618, pam50.robust.1=="Her2" )
get_analysis(datain=temp, outcome="DMFS.lung",xin="ch3q20.19.n.scale+age+node", metflag="no")
```

```
 #################################### Adjust for gse cluster
                  exp(coef) lower .95 upper .95 pvalue    se rob.se
ch3q20.19.n.scale     3.654     0.933    14.310  0.063 0.588  0.697
age>50                1.284     0.602     2.740  0.518 0.819  0.387
nodepos              13.740     3.152    59.883  0.000 0.945  0.751
```

18.3 Lung met in LumA

```
temp=subset(temp618, pam50.robust.1=="LumA" )
get_analysis(datain=temp, outcome="DMFS.lung",xin="ch3q20.19.n.scale+age+node", metflag="no")
```

```
 #################################### Adjust for gse cluster
                  exp(coef) lower .95 upper .95 pvalue    se rob.se
ch3q20.19.n.scale     2.168     1.423     3.301  0.000 0.410  0.215
age>50                2.179     0.892     5.326  0.087 0.699  0.456
nodepos               3.742     0.527    26.565  0.187 0.644  1.000
```

19 Table 2 UniCox model for brain metastasis

19.1 brain met in Basal-like

```
temp=subset(temp618, pam50.robust.1=="Basal" )
get_analysis(datain=temp, outcome="DMFS.brain",xin="ch3q20.19.n.scale", metflag="no")



 ################################### Adjust for gse cluster
                  exp(coef) exp(-coef) lower .95 upper .95 pvalue    se
ch3q20.19.n.scale      1.79      0.559     1.568     2.042      0 0.237
                  rob.se
ch3q20.19.n.scale  0.067
```

19.2 brain met in Her2

```
temp=subset(temp618, pam50.robust.1=="Her2" )
get_analysis(datain=temp, outcome="DMFS.brain",xin="ch3q20.19.n.scale", metflag="no")



 ################################### Adjust for gse cluster
                  exp(coef) exp(-coef) lower .95 upper .95 pvalue   se
ch3q20.19.n.scale     0.886      1.129      0.15     5.246  0.894 0.79
                  rob.se
ch3q20.19.n.scale  0.908
```

19.3 brain met in LumA

```
temp=subset(temp618, pam50.robust.1=="LumA" )
get_analysis(datain=temp, outcome="DMFS.brain",xin="ch3q20.19.n.scale", metflag="no")



 ################################### Adjust for gse cluster
                  exp(coef) exp(-coef) lower .95 upper .95 pvalue    se
ch3q20.19.n.scale     0.353      2.837     0.092      1.35  0.128 1.174
                  rob.se
ch3q20.19.n.scale  0.685
```

19.4 brain met in LumB

```
temp=subset(temp618, pam50.robust.1=="LumB" )
get_analysis(datain=temp, outcome="DMFS.brain",xin="ch3q20.19.n.scale", metflag="no")



 #################################### Adjust for gse cluster
                  exp(coef) exp(-coef) lower .95 upper .95 pvalue    se
ch3q20.19.n.scale     4.401      0.227     3.021     6.413      0 0.401
                  rob.se
ch3q20.19.n.scale  0.192
```

19.5 brain met in Normal

```
temp=subset(temp618, pam50.robust.1=="Normal" )
get_analysis(datain=temp, outcome="DMFS.brain",xin="ch3q20.19.n.scale", metflag="no")



 #################################### Adjust for gse cluster
                  exp(coef) exp(-coef) lower .95 upper .95 pvalue    se
ch3q20.19.n.scale     1.157      0.864     0.214     6.247  0.865 0.638
                  rob.se
ch3q20.19.n.scale   0.86
```

19.6 brain met in TNBC

```
get_analysis(datain=temp.TNBC1, outcome="DMFS.brain",xin="ch3q20.19.n.scale", metflag="no")



 #################################### Adjust for gse cluster
                  exp(coef) exp(-coef) lower .95 upper .95 pvalue    se
ch3q20.19.n.scale     1.463      0.683     1.146     1.868  0.002 0.186
                  rob.se
ch3q20.19.n.scale  0.125
```

19.7 brain met in Non-TNBC

```
get_analysis(datain=temp.TNBC0, outcome="DMFS.brain",xin="ch3q20.19.n.scale", metflag="no")



 ################################### Adjust for gse cluster
                  exp(coef) exp(-coef) lower .95 upper .95 pvalue    se
ch3q20.19.n.scale     2.451      0.408     2.007     2.992      0 0.333
                  rob.se
ch3q20.19.n.scale  0.102
```

20 Table 2 multiCox model for brain metastasis

20.1 see Table S14 for brain met in TNBC, non-TNBC and Basal-like

20.2 Lung met in LumB

```
temp=subset(temp618, pam50.robust.1=="LumB" )
get_analysis(datain=temp, outcome="DMFS.brain",xin="ch3q20.19.n.scale+age+node", metflag="no")



 ################################### Adjust for gse cluster
                  exp(coef) lower .95 upper .95 pvalue    se rob.se
ch3q20.19.n.scale     6.699     2.204    20.359  0.001 0.529  0.567
age>50                0.240     0.055     1.055  0.059 0.945  0.755
nodepos               4.140     0.667    25.692  0.127 0.791  0.931
```

21 Table S13

21.1 Lung TNBC

```
get_analysis(datain=temp.TNBC1, outcome="DMFS.lung",xin="ch3q20.19.n.scale+GENE70+GENE76+lms18.std+tgfb152.n.scale+gene6.n.scale+age+node", m
```

```
 ################################### Adjust for gse cluster
                  exp(coef) lower .95 upper .95 pvalue    se rob.se
ch3q20.19.n.scale     1.443     1.305     1.596  0.000 0.150  0.051
GENE70                0.192     0.061     0.600  0.005 1.726  0.582
GENE76                1.017     1.012     1.022  0.000 0.009  0.003
lms18.std             1.172     1.066     1.288  0.001 0.064  0.048
tgfb152.n.scale       1.108     0.779     1.575  0.567 0.201  0.179
gene6.n.scale         0.979     0.761     1.261  0.872 0.240  0.129
age>50                0.766     0.523     1.122  0.171 0.348  0.195
nodepos               4.665     2.361     9.217  0.000 0.375  0.347
```

21.2 Lung Non-TNBC

```
get_analysis(datain=temp.TNBC0, outcome="DMFS.lung",xin="ch3q20.19.n.scale+GENE70+GENE76+lms18.std+tgfb152.n.scale+gene6.n.scale+age+node", m
```

```
 ################################### Adjust for gse cluster
                  exp(coef) lower .95 upper .95 pvalue    se rob.se
ch3q20.19.n.scale     1.364     0.948     1.961  0.094 0.209  0.185
GENE70               34.548     0.282  4232.841  0.149 1.667  2.453
GENE76                1.016     0.991     1.040  0.207 0.005  0.012
lms18.std             1.055     0.912     1.219  0.474 0.065  0.074
tgfb152.n.scale       1.759     1.152     2.686  0.009 0.237  0.216
gene6.n.scale         0.613     0.376     1.000  0.050 0.407  0.249
age>50                2.156     1.767     2.631  0.000 0.377  0.102
nodepos               3.250     0.934    11.312  0.064 0.355  0.636
```

21.3 Lung Basal-like

```
temp=subset(temp618, pam50.robust.1=="Basal" )
get_analysis(datain=temp, outcome="DMFS.lung",xin="ch3q20.19.n.scale+GENE70+GENE76+lms18.std+tgfb152.n.scale+gene6.n.scale+age+node", metflag



 ################################### Adjust for gse cluster
                  exp(coef) lower .95 upper .95 pvalue    se rob.se
ch3q20.19.n.scale     1.468     1.271     1.696  0.000 0.166  0.074
GENE70                0.217     0.137     0.344  0.000 2.238  0.236
GENE76                1.012     1.000     1.025  0.053 0.010  0.006
lms18.std             1.168     0.994     1.372  0.059 0.071  0.082
tgfb152.n.scale       1.273     0.871     1.861  0.212 0.230  0.194
gene6.n.scale         1.133     0.879     1.461  0.336 0.266  0.130
age>50                0.757     0.470     1.220  0.253 0.380  0.244
nodepos               5.244     4.387     6.269  0.000 0.422  0.091
```

22 Table S14

22.1 Brain TNBC

```
get_analysis(datain=temp.TNBC1, outcome="DMFS.brain",xin="ch3q20.19.n.scale+age+node", metflag="no")



 #################################### Adjust for gse cluster
                  exp(coef) lower .95 upper .95 pvalue    se rob.se
ch3q20.19.n.scale     1.502     1.065     2.118  0.020 0.197  0.175
age>50                0.765     0.259     2.257  0.627 0.497  0.552
nodepos               2.274     1.058     4.889  0.035 0.504  0.391
```

22.2 Brain Non-TNBC

```
get_analysis(datain=temp.TNBC0, outcome="DMFS.brain",xin="ch3q20.19.n.scale+age+node", metflag="no")



 #################################### Adjust for gse cluster
                  exp(coef) lower .95 upper .95 pvalue    se rob.se
ch3q20.19.n.scale     2.437     1.834     3.239  0.000 0.339  0.145
age>50                1.527     1.032     2.260  0.034 0.634  0.200
nodepos               4.886     1.530    15.609  0.007 0.624  0.593
```

22.3 Brain Basal-like

```
temp=subset(temp618, pam50.robust.1=="Basal" )
get_analysis(datain=temp, outcome="DMFS.brain",xin="ch3q20.19.n.scale+age+node", metflag="no")



 #################################### Adjust for gse cluster
                  exp(coef) lower .95 upper .95 pvalue    se rob.se
ch3q20.19.n.scale     1.868     1.573     2.220  0.000 0.268  0.088
age>50                1.043     0.326     3.340  0.943 0.624  0.594
nodepos               2.635     1.038     6.687  0.041 0.635  0.475
```

```r
##### NeoaChemo pt
test.pcr <- subset(all,dataset4 %in% c("gse20194", "gse20271", "gse25066" ,"gse16446"))  ### 1054
test.pcr.TNBC <- subset(test.pcr, TRNeg=="yes")
test.pcr.notTNBC <- subset(test.pcr, TRNeg=="no")
```

23 Table S16

```r
get_lrm(datain=test.pcr,xin="ch3q20.19.n.scale+grade+age+size+node+er+pr+her2")
```

```
 ################################### Adjust for gse cluster
                   Beta    se    OR             CI p-value
ch3q20.19.n.scale  0.276 0.033 1.318 1.236 to 1.406  0.0000
grade=III          1.181 0.044 3.259  2.99 to 3.552  0.0000
age=>50           -0.320 0.071 0.726 0.631 to 0.835  0.0000
size=>2           -0.523 0.310 0.593 0.323 to 1.089  0.0919
node=pos           0.238 0.197 1.268 0.862 to 1.865  0.2276
er=pos            -1.394 0.190 0.248  0.171 to 0.36  0.0000
pr=pos            -0.250 0.358 0.779 0.386 to 1.572  0.4857
her2=pos           0.331 0.471 1.392 0.553 to 3.505  0.4824
```

24 Table S17

24.1 TNBC

```
get_lrm(datain=test.pcr.TNBC,xin="ch3q20.19.n.scale+age+size+grade+node+GENE70+GGI+pcna117.n.scale+gene6.n.scale")
```

```
 ################################### Adjust for gse cluster
                    Beta    se     OR              CI p-value
ch3q20.19.n.scale  0.407 0.064  1.503  1.325 to 1.705  0.0000
age=>50           -0.499 0.117  0.607  0.483 to 0.764  0.0000
size=>2           -0.122 0.199  0.886    0.6 to 1.308  0.5410
grade=III          0.663 0.136  1.940  1.487 to 2.532  0.0000
node=pos           0.377 0.374  1.458    0.7 to 3.037  0.3138
GENE70             2.322 0.374 10.198 4.902 to 21.216  0.0000
GGI               -2.347 0.293  0.096   0.054 to 0.17  0.0000
pcna117.n.scale    0.855 0.095  2.352  1.953 to 2.833  0.0000
gene6.n.scale      0.265 0.073  1.304   1.13 to 1.505  0.0003
```

24.2 non-TNBC

```
get_lrm(datain=test.pcr.notTNBC,xin="ch3q20.19.n.scale+age+size+grade+node+GENE70+GGI+pcna117.n.scale+gene6.n.scale")
```

```
 ################################### Adjust for gse cluster
                    Beta    se     OR             CI p-value
ch3q20.19.n.scale -0.164 0.043  0.849  0.78 to 0.924  0.0002
age=>50            0.261 0.217  1.298 0.849 to 1.986  0.2282
size=>2           -0.839 0.521  0.432 0.156 to 1.199  0.1070
grade=III          1.289 0.175  3.627 2.574 to 5.112  0.0000
node=pos           0.153 0.099  1.165  0.96 to 1.415  0.1224
GENE70             1.069 1.723  2.913  0.1 to 85.277  0.5348
GGI                2.354 0.388 10.530 4.922 to 22.53  0.0000
pcna117.n.scale   -0.590 0.195  0.555 0.378 to 0.813  0.0025
gene6.n.scale      0.428 0.116  1.534 1.222 to 1.926  0.0002
```

25 Table S18

25.1 TNBC

```
temp <- test.pcr.TNBC

get_lrm(datain=temp, xin="age")


 #################################### Adjust for gse cluster
          Beta    se    OR             CI p-value
age=>50 -0.573 0.116 0.564 0.45 to 0.708       0

get_lrm(datain=temp, xin="size")


 #################################### Adjust for gse cluster
         Beta    se    OR             CI p-value
size=>2 0.016 0.241 1.016 0.633 to 1.629  0.9487

get_lrm(datain=temp, xin="grade")


 #################################### Adjust for gse cluster
          Beta   se    OR             CI p-value
grade=III 0.93 0.16 2.536 1.852 to 3.472       0

get_lrm(datain=temp, xin="node")


 #################################### Adjust for gse cluster
          Beta    se    OR             CI p-value
node=pos 0.166 0.302 1.181 0.654 to 2.133  0.5809

get_lrm(datain=temp, xin="ch3q20.19.n.scale")


 #################################### Adjust for gse cluster
                   Beta    se    OR             CI p-value
ch3q20.19.n.scale 0.525 0.036 1.691 1.576 to 1.815       0

get_lrm(datain=temp, xin="GENE70")
```

```
##################################### Adjust for gse cluster
       Beta    se     OR              CI p-value
GENE70 3.559 0.661 35.125 9.615 to 128.324       0

get_lrm(datain=temp, xin="GENE76")


##################################### Adjust for gse cluster
        Beta    se    OR           CI p-value
GENE76 -0.005 0.002 0.995 0.99 to 0.999  0.0138

get_lrm(datain=temp, xin="gene6.n.scale")


##################################### Adjust for gse cluster
             Beta    se    OR           CI p-value
gene6.n.scale 0.483 0.099 1.621 1.334 to 1.97       0

get_lrm(datain=temp, xin="tgfb152.n.scale")


##################################### Adjust for gse cluster
                Beta    se    OR             CI p-value
tgfb152.n.scale -0.159 0.037 0.853 0.794 to 0.917       0

get_lrm(datain=temp, xin="pcna117.n.scale")


##################################### Adjust for gse cluster
               Beta    se    OR             CI p-value
pcna117.n.scale 0.511 0.095 1.668 1.385 to 2.007       0

get_lrm(datain=temp, xin="GGI")


##################################### Adjust for gse cluster
    Beta    se   OR             CI p-value
GGI 0.932 0.204 2.54 1.704 to 3.788       0
```

25.2 Non-TNBC

```
temp <- test.pcr.notTNBC

get_lrm(datain=temp, xin="age")


 ##################################### Adjust for gse cluster
         Beta    se    OR             CI p-value
age=>50 0.103 0.092 1.109 0.925 to 1.329  0.2627

get_lrm(datain=temp, xin="size")


 ##################################### Adjust for gse cluster
          Beta    se    OR             CI p-value
size=>2 -0.054 0.562 0.948 0.315 to 2.852  0.9237

get_lrm(datain=temp, xin="grade")


 ##################################### Adjust for gse cluster
           Beta    se    OR             CI p-value
grade=III 1.748 0.179 5.743 4.047 to 8.148       0

get_lrm(datain=temp, xin="node")


 ##################################### Adjust for gse cluster
          Beta    se   OR             CI p-value
node=pos 0.372 0.106 1.45 1.178 to 1.785   5e-04

get_lrm(datain=temp, xin="ch3q20.19.n.scale")


 ##################################### Adjust for gse cluster
                   Beta    se   OR             CI p-value
ch3q20.19.n.scale 0.165 0.038 1.18 1.094 to 1.272       0

get_lrm(datain=temp, xin="GENE70")


 ##################################### Adjust for gse cluster
        Beta    se      OR                 CI p-value
GENE70 5.947 0.387 382.483 179.097 to 816.836       0

get_lrm(datain=temp, xin="GENE76")
```

```
 #################################### Adjust for gse cluster
        Beta    se    OR             CI p-value
GENE76 0.005 0.004 1.005 0.996 to 1.013  0.2699

get_lrm(datain=temp, xin="gene6.n.scale")


 #################################### Adjust for gse cluster
               Beta    se    OR             CI p-value
gene6.n.scale 0.352 0.207 1.422 0.948 to 2.133   0.089

get_lrm(datain=temp, xin="tgfb152.n.scale")


 #################################### Adjust for gse cluster
                  Beta    se    OR             CI p-value
tgfb152.n.scale -0.112 0.122 0.894 0.704 to 1.137  0.3611

get_lrm(datain=temp, xin="pcna117.n.scale")


 #################################### Adjust for gse cluster
                 Beta    se    OR             CI p-value
pcna117.n.scale 0.793 0.236 2.211 1.391 to 3.514   8e-04

get_lrm(datain=temp, xin="GGI")


 #################################### Adjust for gse cluster
     Beta    se    OR             CI p-value
GGI 1.654 0.442 5.228 2.199 to 12.43   2e-04
```

26 Table S19 lung metastasis

26.1 TNBC

```
selgene =paste0(Cs(ABCC5 , ACTL6A , ATP11B , DCUN1D1 , DLG1 , DVL3 , EIF2B5 , FXR1 , LSG1 , MFN1 , NCBP2 , NDUFB5 , PIK3CA , PRKCI , PSMD2  ,



for ( i in 1:length(selgene)) {
 get_analysis(datain=temp.TNBC1, outcome="DMFS.lung",xin=selgene[i], metflag="no")

}



 ################################### Adjust for gse cluster
                exp(coef) lower .95 upper .95 pvalue    se rob.se
ABCC5               2.018     1.202     3.389  0.008 0.377  0.264
age>50              0.820     0.524     1.284  0.386 0.336  0.229
nodepos             4.098     2.385     7.040  0.000 0.379  0.276
GENE70              0.517     0.169     1.587  0.249 1.695  0.572
GENE76              1.020     1.013     1.026  0.000 0.009  0.003
lms18.std           1.161     1.065     1.266  0.001 0.063  0.044
tgfb152.n.scale     1.146     0.747     1.759  0.533 0.212  0.219
gene6.n.scale       0.999     0.819     1.218  0.993 0.230  0.101


 ################################### Adjust for gse cluster
                exp(coef) lower .95 upper .95 pvalue    se rob.se
ACTL6A              1.621     1.012     2.596  0.044 0.259  0.240
age>50              0.838     0.539     1.301  0.431 0.345  0.225
nodepos             4.976     2.540     9.749  0.000 0.375  0.343
GENE70              0.211     0.055     0.816  0.024 1.750  0.690
GENE76              1.020     1.016     1.024  0.000 0.009  0.002
lms18.std           1.178     1.064     1.305  0.002 0.065  0.052
tgfb152.n.scale     1.170     0.809     1.691  0.405 0.207  0.188
gene6.n.scale       0.912     0.711     1.170  0.469 0.234  0.127


 ################################### Adjust for gse cluster
                exp(coef) lower .95 upper .95 pvalue    se rob.se
ATP11B              2.198     1.477     3.271  0.000 0.314  0.203
```

```
age>50              0.812     0.567     1.161  0.254 0.349  0.183
nodepos             5.045     2.424    10.499  0.000 0.378  0.374
GENE70              0.184     0.031     1.112  0.065 1.730  0.917
GENE76              1.011     1.004     1.018  0.001 0.009  0.003
lms18.std           1.214     1.098     1.343  0.000 0.066  0.051
tgfb152.n.scale     0.996     0.645     1.536  0.984 0.215  0.221
gene6.n.scale       1.076     0.854     1.356  0.535 0.242  0.118


 #################################### Adjust for gse cluster
                exp(coef) lower .95 upper .95 pvalue    se rob.se
DCUN1D1             1.582     0.874     2.863  0.130 0.468  0.303
age>50              0.798     0.511     1.246  0.321 0.351  0.227
nodepos             4.458     2.121     9.373  0.000 0.371  0.379
GENE70              0.317     0.037     2.708  0.294 1.761  1.094
GENE76              1.017     1.011     1.023  0.000 0.009  0.003
lms18.std           1.193     1.086     1.310  0.000 0.066  0.048
tgfb152.n.scale     1.129     0.747     1.704  0.565 0.210  0.210
gene6.n.scale       0.991     0.823     1.194  0.926 0.231  0.095


 #################################### Adjust for gse cluster
                exp(coef) lower .95 upper .95 pvalue    se rob.se
DLG1                0.973     0.522     1.814  0.931 0.381  0.318
age>50              0.846     0.534     1.340  0.476 0.346  0.235
nodepos             4.760     2.419     9.369  0.000 0.368  0.345
GENE70              0.602     0.125     2.899  0.527 1.682  0.802
GENE76              1.017     1.012     1.022  0.000 0.009  0.002
lms18.std           1.178     1.089     1.274  0.000 0.065  0.040
tgfb152.n.scale     1.181     0.830     1.682  0.354 0.216  0.180
gene6.n.scale       0.985     0.814     1.192  0.874 0.227  0.097


 #################################### Adjust for gse cluster
                exp(coef) lower .95 upper .95 pvalue    se rob.se
DVL3                1.691     0.613     4.664  0.310 0.356  0.518
age>50              0.834     0.564     1.233  0.363 0.344  0.199
nodepos             4.873     2.456     9.671  0.000 0.370  0.350
GENE70              0.615     0.140     2.708  0.521 1.635  0.756
GENE76              1.017     1.011     1.023  0.000 0.009  0.003
lms18.std           1.164     1.073     1.264  0.000 0.064  0.042
tgfb152.n.scale     1.170     0.766     1.785  0.467 0.209  0.216
```

```
gene6.n.scale       0.951     0.712     1.270  0.732 0.233  0.148


 #################################### Adjust for gse cluster
                exp(coef) lower .95 upper .95 pvalue    se rob.se
EIF2B5              2.089     1.195     3.649  0.010 0.474  0.285
age>50              0.796     0.528     1.201  0.278 0.348  0.210
nodepos             4.447     2.162     9.150  0.000 0.369  0.368
GENE70              0.305     0.063     1.464  0.138 1.714  0.801
GENE76              1.016     1.010     1.021  0.000 0.009  0.003
lms18.std           1.175     1.091     1.266  0.000 0.066  0.038
tgfb152.n.scale     1.200     0.788     1.829  0.395 0.202  0.215
gene6.n.scale       1.044     0.889     1.225  0.600 0.238  0.082


 #################################### Adjust for gse cluster
                exp(coef) lower .95 upper .95 pvalue    se rob.se
FXR1                1.638     1.394     1.924  0.000 0.346  0.082
age>50              0.821     0.523     1.287  0.389 0.351  0.230
nodepos             4.477     2.147     9.336  0.000 0.368  0.375
GENE70              0.532     0.123     2.301  0.398 1.670  0.747
GENE76              1.015     1.008     1.023  0.000 0.009  0.004
lms18.std           1.163     1.096     1.235  0.000 0.065  0.031
tgfb152.n.scale     1.162     0.776     1.740  0.466 0.202  0.206
gene6.n.scale       0.946     0.751     1.191  0.635 0.239  0.118


 #################################### Adjust for gse cluster
                exp(coef) lower .95 upper .95 pvalue    se rob.se
LSG1                1.322     0.506     3.456  0.569 0.366  0.490
age>50              0.774     0.406     1.474  0.435 0.365  0.329
nodepos             4.801     2.222    10.376  0.000 0.366  0.393
GENE70              0.414     0.183     0.937  0.034 1.709  0.416
GENE76              1.018     1.010     1.026  0.000 0.009  0.004
lms18.std           1.183     1.096     1.276  0.000 0.065  0.039
tgfb152.n.scale     1.192     0.780     1.822  0.417 0.207  0.216
gene6.n.scale       1.015     0.746     1.381  0.925 0.234  0.157


 #################################### Adjust for gse cluster
                exp(coef) lower .95 upper .95 pvalue    se rob.se
MFN1                1.829     1.415     2.363  0.000 0.283  0.131
```

```
age>50               0.748     0.517     1.083  0.124 0.351  0.189
nodepos              4.751     2.299     9.817  0.000 0.374  0.370
GENE70               0.242     0.062     0.947  0.042 1.719  0.695
GENE76               1.013     1.007     1.019  0.000 0.009  0.003
lms18.std            1.185     1.069     1.315  0.001 0.065  0.053
tgfb152.n.scale      1.185     0.843     1.668  0.329 0.202  0.174
gene6.n.scale        1.031     0.835     1.272  0.780 0.237  0.108


 ################################### Adjust for gse cluster
                exp(coef) lower .95 upper .95 pvalue    se rob.se
NCBP2                1.364     1.052     1.770  0.019 0.300  0.133
age>50               0.804     0.489     1.324  0.392 0.352  0.254
nodepos              4.693     2.426     9.077  0.000 0.368  0.337
GENE70               0.398     0.115     1.382  0.147 1.704  0.635
GENE76               1.018     1.011     1.025  0.000 0.009  0.003
lms18.std            1.184     1.099     1.275  0.000 0.064  0.038
tgfb152.n.scale      1.149     0.782     1.687  0.480 0.206  0.196
gene6.n.scale        0.980     0.788     1.221  0.860 0.234  0.112


 ################################### Adjust for gse cluster
                exp(coef) lower .95 upper .95 pvalue    se rob.se
NDUFB5               3.491     1.952     6.243  0.000 0.364  0.297
age>50               0.714     0.432     1.180  0.189 0.351  0.256
nodepos              4.590     2.464     8.551  0.000 0.365  0.317
GENE70               0.232     0.109     0.493  0.000 1.730  0.384
GENE76               1.015     1.010     1.020  0.000 0.009  0.003
lms18.std            1.191     1.121     1.266  0.000 0.062  0.031
tgfb152.n.scale      1.061     0.798     1.411  0.682 0.193  0.145
gene6.n.scale        1.094     0.920     1.302  0.310 0.240  0.089


 ################################### Adjust for gse cluster
                exp(coef) lower .95 upper .95 pvalue    se rob.se
PIK3CA               1.391     1.318     1.469  0.000 0.170  0.028
age>50               0.804     0.506     1.276  0.354 0.356  0.236
nodepos              4.741     2.312     9.719  0.000 0.369  0.366
GENE70               0.794     0.295     2.138  0.649 1.646  0.505
GENE76               1.016     1.011     1.021  0.000 0.009  0.002
lms18.std            1.165     1.077     1.261  0.000 0.066  0.040
tgfb152.n.scale      1.151     0.731     1.811  0.544 0.207  0.231
```

```
gene6.n.scale       0.978     0.800     1.195  0.827 0.232  0.102


 ################################### Adjust for gse cluster
                exp(coef) lower .95 upper .95 pvalue    se rob.se
PRKCI               1.402     0.947     2.074  0.091 0.292  0.200
age>50              0.835     0.521     1.337  0.453 0.351  0.240
nodepos             4.973     2.991     8.266  0.000 0.373  0.259
GENE70              0.605     0.162     2.263  0.455 1.654  0.674
GENE76              1.016     1.010     1.023  0.000 0.009  0.003
lms18.std           1.188     1.082     1.304  0.000 0.065  0.048
tgfb152.n.scale     1.152     0.767     1.730  0.494 0.208  0.207
gene6.n.scale       0.886     0.637     1.233  0.474 0.250  0.169


 ################################### Adjust for gse cluster
                exp(coef) lower .95 upper .95 pvalue    se rob.se
PSMD2               2.453     1.672     3.599  0.000 0.393  0.196
age>50              0.751     0.423     1.336  0.330 0.351  0.294
nodepos             4.637     2.431     8.845  0.000 0.369  0.329
GENE70              0.198     0.043     0.912  0.038 1.718  0.778
GENE76              1.023     1.012     1.034  0.000 0.010  0.006
lms18.std           1.167     1.082     1.259  0.000 0.064  0.039
tgfb152.n.scale     1.146     0.851     1.544  0.369 0.201  0.152
gene6.n.scale       0.928     0.721     1.195  0.562 0.235  0.129


 ################################### Adjust for gse cluster
                exp(coef) lower .95 upper .95 pvalue    se rob.se
SENP2               1.481     0.583     3.758  0.409 0.591  0.475
age>50              0.828     0.537     1.277  0.393 0.347  0.221
nodepos             4.660     2.415     8.992  0.000 0.366  0.335
GENE70              0.637     0.135     2.995  0.568 1.657  0.790
GENE76              1.018     1.013     1.022  0.000 0.009  0.002
lms18.std           1.173     1.079     1.276  0.000 0.065  0.043
tgfb152.n.scale     1.220     0.811     1.837  0.339 0.213  0.209
gene6.n.scale       0.949     0.769     1.171  0.626 0.237  0.107


 ################################### Adjust for gse cluster
                exp(coef) lower .95 upper .95 pvalue    se rob.se
SENP5               1.273     0.555     2.923  0.569 0.522  0.424
```

```
age>50               0.843     0.535     1.327  0.461 0.345  0.232
nodepos              4.721     2.412     9.239  0.000 0.365  0.343
GENE70               0.468     0.236     0.928  0.030 1.712  0.349
GENE76               1.017     1.010     1.023  0.000 0.009  0.003
lms18.std            1.178     1.083     1.280  0.000 0.065  0.043
tgfb152.n.scale      1.167     0.765     1.779  0.473 0.209  0.215
gene6.n.scale        1.002     0.775     1.296  0.985 0.233  0.131


 ################################### Adjust for gse cluster
                exp(coef) lower .95 upper .95 pvalue    se rob.se
UBXN7                1.047     0.853     1.284  0.660 0.274  0.104
age>50               0.845     0.532     1.342  0.477 0.346  0.236
nodepos              4.740     2.395     9.380  0.000 0.365  0.348
GENE70               0.562     0.124     2.556  0.456 1.662  0.772
GENE76               1.017     1.011     1.024  0.000 0.009  0.003
lms18.std            1.177     1.088     1.272  0.000 0.065  0.040
tgfb152.n.scale      1.175     0.761     1.813  0.467 0.209  0.221
gene6.n.scale        0.985     0.812     1.194  0.875 0.228  0.098


 ################################### Adjust for gse cluster
                exp(coef) lower .95 upper .95 pvalue    se rob.se
ZNF639              29.304     3.005   285.814  0.004 1.178  1.162
age>50               0.809     0.572     1.145  0.232 0.351  0.177
nodepos              5.325     3.053     9.288  0.000 0.375  0.284
GENE70               0.660     0.193     2.255  0.507 1.649  0.627
GENE76               1.015     1.008     1.021  0.000 0.009  0.003
lms18.std            1.179     1.099     1.265  0.000 0.064  0.036
tgfb152.n.scale      1.157     0.771     1.736  0.481 0.201  0.207
gene6.n.scale        0.952     0.813     1.114  0.541 0.235  0.080
```

26.2 non-TNBC

```
for ( i in 1:length(selgene)) {
 get_analysis(datain=temp.TNBC0, outcome="DMFS.lung",xin=selgene[i], metflag="no")

}
```

```
 #################################### Adjust for gse cluster
               exp(coef) lower .95 upper .95 pvalue    se rob.se
ABCC5              1.138     0.677     1.912  0.627 0.271  0.265
age>50             2.163     1.755     2.665  0.000 0.377  0.107
nodepos            3.266     0.861    12.385  0.082 0.361  0.680
GENE70            61.188     0.877  4268.230  0.058 1.690  2.166
GENE76             1.017     0.995     1.039  0.138 0.006  0.011
lms18.std          1.041     0.905     1.197  0.576 0.064  0.072
tgfb152.n.scale    1.906     1.301     2.792  0.001 0.236  0.195
gene6.n.scale      0.629     0.379     1.045  0.073 0.413  0.259


 #################################### Adjust for gse cluster
               exp(coef) lower .95 upper .95 pvalue    se rob.se
ACTL6A             1.329     0.923     1.915  0.127 0.291  0.186
age>50             2.200     1.802     2.686  0.000 0.376  0.102
nodepos            3.389     1.023    11.223  0.046 0.356  0.611
GENE70            37.089     0.484  2840.120  0.103 1.674  2.213
GENE76             1.017     0.990     1.044  0.213 0.005  0.013
lms18.std          1.046     0.906     1.206  0.541 0.064  0.073
tgfb152.n.scale    1.876     1.260     2.793  0.002 0.234  0.203
gene6.n.scale      0.612     0.409     0.917  0.017 0.411  0.206


 #################################### Adjust for gse cluster
               exp(coef) lower .95 upper .95 pvalue    se rob.se
ATP11B             1.000     0.898     1.114  0.998 0.306  0.055
age>50             2.172     1.735     2.718  0.000 0.380  0.115
nodepos            3.365     0.962    11.768  0.057 0.358  0.639
GENE70            51.427     0.423  6255.260  0.108 1.670  2.450
GENE76             1.018     0.994     1.043  0.151 0.005  0.012
lms18.std          1.039     0.906     1.192  0.582 0.063  0.070
tgfb152.n.scale    1.945     1.315     2.877  0.001 0.249  0.200
gene6.n.scale      0.621     0.409     0.942  0.025 0.411  0.213


 #################################### Adjust for gse cluster
               exp(coef) lower .95 upper .95 pvalue    se rob.se
DCUN1D1            1.482     0.702     3.130  0.302 0.455  0.381
age>50             2.117     1.655     2.708  0.000 0.376  0.126
nodepos            3.105     0.877    10.995  0.079 0.367  0.645
```

```
GENE70              40.489     0.602  2722.006  0.085 1.650  2.147
GENE76               1.017     0.992     1.043  0.186 0.005  0.013
lms18.std            1.052     0.908     1.218  0.499 0.065  0.075
tgfb152.n.scale      1.823     1.144     2.906  0.012 0.243  0.238
gene6.n.scale        0.589     0.344     1.006  0.053 0.412  0.274


 #################################### Adjust for gse cluster
                exp(coef) lower .95 upper .95 pvalue    se rob.se
DLG1                 1.005     0.833     1.214  0.957 0.458  0.096
age>50               2.172     1.762     2.676  0.000 0.375  0.107
nodepos              3.365     0.972    11.644  0.055 0.356  0.633
GENE70              51.288     0.428  6150.009  0.107 1.652  2.442
GENE76               1.018     0.994     1.043  0.151 0.005  0.012
lms18.std            1.039     0.906     1.192  0.583 0.064  0.070
tgfb152.n.scale      1.944     1.318     2.867  0.001 0.241  0.198
gene6.n.scale        0.620     0.417     0.922  0.018 0.417  0.202


 #################################### Adjust for gse cluster
                exp(coef) lower .95 upper .95 pvalue    se rob.se
DVL3                 0.708     0.307     1.634  0.418 0.363  0.427
age>50               2.223     1.880     2.629  0.000 0.378  0.085
nodepos              3.423     1.022    11.464  0.046 0.358  0.617
GENE70              50.791     0.412  6262.456  0.110 1.662  2.456
GENE76               1.018     0.994     1.043  0.137 0.005  0.012
lms18.std            1.040     0.905     1.195  0.582 0.063  0.071
tgfb152.n.scale      1.975     1.397     2.793  0.000 0.234  0.177
gene6.n.scale        0.573     0.313     1.051  0.072 0.419  0.309


 #################################### Adjust for gse cluster
                exp(coef) lower .95 upper .95 pvalue    se rob.se
EIF2B5               1.032     0.467     2.281  0.938 0.460  0.405
age>50               2.171     1.748     2.696  0.000 0.376  0.111
nodepos              3.355     0.932    12.080  0.064 0.359  0.654
GENE70              51.359     0.464  5689.660  0.101 1.639  2.402
GENE76               1.018     0.993     1.044  0.165 0.005  0.013
lms18.std            1.040     0.896     1.207  0.609 0.064  0.076
tgfb152.n.scale      1.946     1.330     2.845  0.001 0.233  0.194
gene6.n.scale        0.620     0.400     0.961  0.033 0.411  0.224
```

```
##################################### Adjust for gse cluster
                 exp(coef) lower .95 upper .95 pvalue    se rob.se
FXR1                 1.526     0.796     2.924  0.203 0.377  0.332
age>50               2.212     1.843     2.654  0.000 0.377  0.093
nodepos              3.423     1.008    11.625  0.049 0.357  0.624
GENE70              47.621     0.522  4343.907  0.093 1.635  2.303
GENE76               1.017     0.994     1.042  0.151 0.005  0.012
lms18.std            1.049     0.906     1.216  0.521 0.064  0.075
tgfb152.n.scale      1.826     1.164     2.863  0.009 0.235  0.230
gene6.n.scale        0.587     0.354     0.972  0.038 0.412  0.258


##################################### Adjust for gse cluster
                 exp(coef) lower .95 upper .95 pvalue    se rob.se
LSG1                 1.084     0.530     2.216  0.825 0.408  0.365
age>50               2.177     1.766     2.682  0.000 0.376  0.107
nodepos              3.360     0.964    11.706  0.057 0.357  0.637
GENE70              48.583     0.549  4298.392  0.090 1.665  2.287
GENE76               1.018     0.993     1.043  0.160 0.005  0.013
lms18.std            1.040     0.903     1.197  0.587 0.064  0.072
tgfb152.n.scale      1.949     1.340     2.834  0.000 0.233  0.191
gene6.n.scale        0.626     0.422     0.928  0.020 0.412  0.201


##################################### Adjust for gse cluster
                 exp(coef) lower .95 upper .95 pvalue    se rob.se
MFN1                 1.519     1.330     1.734  0.000 0.316  0.068
age>50               2.033     1.659     2.493  0.000 0.379  0.104
nodepos              3.146     0.849    11.660  0.086 0.354  0.668
GENE70              44.135     0.397  4902.947  0.115 1.632  2.403
GENE76               1.016     0.992     1.040  0.198 0.005  0.012
lms18.std            1.050     0.921     1.196  0.467 0.064  0.067
tgfb152.n.scale      1.766     1.231     2.533  0.002 0.238  0.184
gene6.n.scale        0.628     0.398     0.991  0.046 0.408  0.233


##################################### Adjust for gse cluster
                 exp(coef) lower .95 upper .95 pvalue    se rob.se
NCBP2                0.820     0.529     1.270  0.374 0.395  0.224
age>50               2.145     1.750     2.629  0.000 0.376  0.104
nodepos              3.392     0.954    12.061  0.059 0.358  0.647
```

```
GENE70             57.926     0.568  5910.383  0.085 1.657  2.360
GENE76              1.018     0.994     1.043  0.146 0.005  0.012
lms18.std           1.032     0.884     1.206  0.687 0.065  0.079
tgfb152.n.scale     1.992     1.282     3.093  0.002 0.239  0.225
gene6.n.scale       0.629     0.415     0.952  0.028 0.413  0.212


 ################################### Adjust for gse cluster
                exp(coef) lower .95 upper .95 pvalue    se rob.se
NDUFB5              2.018     1.290     3.156  0.002 0.391  0.228
age>50              2.093     1.741     2.515  0.000 0.378  0.094
nodepos             3.477     0.998    12.109  0.050 0.357  0.637
GENE70             37.877     0.366  3921.265  0.125 1.654  2.367
GENE76              1.017     0.993     1.041  0.173 0.005  0.012
lms18.std           1.077     0.928     1.249  0.329 0.067  0.076
tgfb152.n.scale     1.696     1.140     2.522  0.009 0.238  0.203
gene6.n.scale       0.589     0.398     0.871  0.008 0.407  0.199


 ################################### Adjust for gse cluster
                exp(coef) lower .95 upper .95 pvalue    se rob.se
PIK3CA              1.472     0.925     2.341  0.103 0.269  0.237
age>50              2.190     1.774     2.703  0.000 0.375  0.107
nodepos             3.431     1.147    10.259  0.027 0.355  0.559
GENE70             67.544     1.113  4099.535  0.044 1.637  2.095
GENE76              1.016     0.993     1.040  0.177 0.005  0.012
lms18.std           1.041     0.911     1.189  0.554 0.063  0.068
tgfb152.n.scale     1.861     1.227     2.822  0.003 0.232  0.212
gene6.n.scale       0.658     0.347     1.245  0.198 0.406  0.326


 ################################### Adjust for gse cluster
                exp(coef) lower .95 upper .95 pvalue    se rob.se
PRKCI               1.033     0.712     1.500  0.863 0.326  0.190
age>50              2.171     1.766     2.669  0.000 0.375  0.105
nodepos             3.388     1.057    10.858  0.040 0.363  0.594
GENE70             50.711     0.416  6175.957  0.109 1.640  2.450
GENE76              1.018     0.994     1.043  0.150 0.005  0.012
lms18.std           1.038     0.911     1.184  0.576 0.064  0.067
tgfb152.n.scale     1.948     1.329     2.856  0.001 0.234  0.195
gene6.n.scale       0.620     0.408     0.944  0.026 0.411  0.214
```

```
 #################################### Adjust for gse cluster
                exp(coef) lower .95 upper .95 pvalue    se rob.se
PSMD2               1.795     0.967     3.332  0.064 0.473  0.316
age>50              2.176     1.779     2.661  0.000 0.376  0.103
nodepos             3.605     1.128    11.522  0.031 0.363  0.593
GENE70             23.338     0.192  2842.174  0.199 1.779  2.450
GENE76              1.017     0.993     1.041  0.168 0.005  0.012
lms18.std           1.053     0.916     1.210  0.467 0.064  0.071
tgfb152.n.scale     1.868     1.250     2.791  0.002 0.233  0.205
gene6.n.scale       0.670     0.451     0.997  0.048 0.414  0.203


 #################################### Adjust for gse cluster
                exp(coef) lower .95 upper .95 pvalue    se rob.se
SENP2               2.462     0.704     8.607  0.158 0.551  0.639
age>50              2.314     1.727     3.101  0.000 0.377  0.149
nodepos             3.256     0.986    10.756  0.053 0.356  0.610
GENE70             92.269     1.922  4429.772  0.022 1.675  1.975
GENE76              1.016     0.993     1.040  0.173 0.005  0.012
lms18.std           1.026     0.904     1.165  0.688 0.063  0.065
tgfb152.n.scale     2.121     1.374     3.273  0.001 0.235  0.221
gene6.n.scale       0.576     0.483     0.686  0.000 0.407  0.089


 #################################### Adjust for gse cluster
                exp(coef) lower .95 upper .95 pvalue    se rob.se
SENP5               1.157     0.663     2.019  0.609 0.581  0.284
age>50              2.155     1.700     2.732  0.000 0.377  0.121
nodepos             3.326     0.966    11.449  0.057 0.359  0.631
GENE70             48.685     0.543  4367.910  0.090 1.658  2.294
GENE76              1.018     0.993     1.043  0.163 0.005  0.013
lms18.std           1.040     0.907     1.192  0.574 0.064  0.070
tgfb152.n.scale     1.947     1.330     2.852  0.001 0.233  0.195
gene6.n.scale       0.614     0.385     0.979  0.041 0.411  0.238


 #################################### Adjust for gse cluster
                exp(coef) lower .95 upper .95 pvalue    se rob.se
UBXN7               2.475     1.772     3.456  0.000 0.319  0.170
age>50              2.177     2.044     2.317  0.000 0.381  0.032
nodepos             3.748     1.397    10.053  0.009 0.367  0.503
```

```
GENE70              51.647     1.248  2136.770  0.038 1.653  1.899
GENE76               1.019     1.000     1.039  0.052 0.005  0.010
lms18.std            1.027     0.905     1.166  0.675 0.064  0.064
tgfb152.n.scale      2.163     1.636     2.860  0.000 0.235  0.143
gene6.n.scale        0.534     0.287     0.993  0.048 0.395  0.316


 ################################### Adjust for gse cluster
                exp(coef) lower .95 upper .95 pvalue    se rob.se
ZNF639               2.412     0.098    59.280  0.590 1.225  1.634
age>50               2.108     1.482     2.999  0.000 0.376  0.180
nodepos              3.292     0.982    11.039  0.054 0.356  0.617
GENE70              45.284     0.691  2965.597  0.074 1.635  2.134
GENE76               1.017     0.991     1.045  0.203 0.005  0.013
lms18.std            1.048     0.903     1.217  0.537 0.065  0.076
tgfb152.n.scale      1.871     1.140     3.071  0.013 0.239  0.253
gene6.n.scale        0.597     0.344     1.036  0.067 0.418  0.281
```

27 Table S19 brain metastasis

27.1 TNBC

```
selgene =paste0(Cs(ABCC5 , ACTL6A , ATP11B , DCUN1D1 , DLG1 , DVL3 , EIF2B5 , FXR1 , LSG1 , MFN1 , NCBP2 , NDUFB5 , PIK3CA , PRKCI , PSMD2  ,


for ( i in 1:length(selgene)) {
 get_analysis(datain=temp.TNBC1, outcome="DMFS.brain",xin=selgene[i], metflag="no")

}



 #################################### Adjust for gse cluster
                exp(coef) lower .95 upper .95 pvalue    se rob.se
ABCC5               0.822     0.183     3.696  0.799 0.553  0.767
age>50              0.884     0.327     2.388  0.808 0.490  0.507
nodepos             2.419     0.829     7.057  0.106 0.529  0.546
pcna117.n.scale     0.989     0.805     1.215  0.918 0.222  0.105


 #################################### Adjust for gse cluster
                exp(coef) lower .95 upper .95 pvalue    se rob.se
ACTL6A              1.633     0.871     3.062  0.126 0.405  0.321
age>50              0.844     0.363     1.964  0.694 0.490  0.431
nodepos             2.246     0.883     5.716  0.089 0.504  0.476
pcna117.n.scale     0.777     0.541     1.116  0.172 0.293  0.185


 #################################### Adjust for gse cluster
                exp(coef) lower .95 upper .95 pvalue    se rob.se
ATP11B              2.506     1.380     4.550  0.003 0.416  0.304
age>50              0.855     0.354     2.067  0.728 0.490  0.451
nodepos             2.315     0.837     6.405  0.106 0.509  0.519
pcna117.n.scale     0.889     0.663     1.190  0.429 0.229  0.149


 #################################### Adjust for gse cluster
                exp(coef) lower .95 upper .95 pvalue    se rob.se
DCUN1D1             4.433     3.755     5.232  0.000 0.593  0.085
age>50              0.748     0.269     2.082  0.579 0.503  0.522
```

```
nodepos             1.719     0.693     4.262  0.242 0.511  0.463
pcna117.n.scale     0.747     0.562     0.993  0.045 0.258  0.145


 ################################### Adjust for gse cluster
                exp(coef) lower .95 upper .95 pvalue    se rob.se
DLG1                2.960     1.047     8.364  0.041 0.564  0.530
age>50              0.870     0.331     2.282  0.776 0.492  0.492
nodepos             2.022     0.870     4.701  0.102 0.511  0.431
pcna117.n.scale     0.925     0.744     1.150  0.482 0.227  0.111


 ################################### Adjust for gse cluster
                exp(coef) lower .95 upper .95 pvalue    se rob.se
DVL3                2.064     1.927     2.211  0.000 0.527  0.035
age>50              0.825     0.335     2.036  0.677 0.491  0.461
nodepos             2.406     0.955     6.058  0.063 0.507  0.471
pcna117.n.scale     0.922     0.670     1.269  0.618 0.225  0.163


 ################################### Adjust for gse cluster
                exp(coef) lower .95 upper .95 pvalue    se rob.se
EIF2B5              2.179     1.170     4.057  0.014 0.744  0.317
age>50              0.849     0.296     2.430  0.760 0.492  0.537
nodepos             2.168     0.958     4.907  0.063 0.512  0.417
pcna117.n.scale     0.906     0.728     1.127  0.375 0.234  0.111


 ################################### Adjust for gse cluster
                exp(coef) lower .95 upper .95 pvalue    se rob.se
FXR1                1.496     0.799     2.801  0.208 0.520  0.320
age>50              0.874     0.314     2.434  0.796 0.491  0.523
nodepos             2.202     0.971     4.992  0.059 0.509  0.418
pcna117.n.scale     0.907     0.758     1.087  0.290 0.240  0.092


 ################################### Adjust for gse cluster
                exp(coef) lower .95 upper .95 pvalue    se rob.se
LSG1                1.331     0.277     6.382  0.721 0.581  0.800
age>50              0.803     0.203     3.182  0.754 0.528  0.703
nodepos             2.249     0.939     5.386  0.069 0.506  0.446
pcna117.n.scale     0.938     0.852     1.031  0.184 0.236  0.049
```

```
 ################################### Adjust for gse cluster
               exp(coef) lower .95 upper .95 pvalue    se rob.se
MFN1               1.647     0.729     3.721  0.230 0.381  0.416
age>50             0.832     0.332     2.081  0.694 0.489  0.468
nodepos            2.079     0.809     5.344  0.129 0.511  0.482
pcna117.n.scale    0.870     0.764     0.990  0.035 0.240  0.066


 ################################### Adjust for gse cluster
               exp(coef) lower .95 upper .95 pvalue    se rob.se
NCBP2              1.538     1.006     2.350  0.047 0.488  0.216
age>50             0.825     0.273     2.486  0.732 0.497  0.563
nodepos            2.176     0.957     4.946  0.063 0.510  0.419
pcna117.n.scale    0.899     0.731     1.105  0.311 0.240  0.106


 ################################### Adjust for gse cluster
               exp(coef) lower .95 upper .95 pvalue    se rob.se
NDUFB5             5.427     1.923    15.317  0.001 0.505  0.529
age>50             0.732     0.326     1.642  0.449 0.496  0.412
nodepos            1.996     0.894     4.459  0.092 0.504  0.410
pcna117.n.scale    0.886     0.816     0.963  0.004 0.222  0.042


 ################################### Adjust for gse cluster
               exp(coef) lower .95 upper .95 pvalue    se rob.se
PIK3CA             1.720     1.352     2.188  0.000 0.241  0.123
age>50             0.821     0.300     2.251  0.702 0.499  0.515
nodepos            2.281     1.020     5.103  0.045 0.518  0.411
pcna117.n.scale    0.934     0.742     1.177  0.564 0.216  0.118


 ################################### Adjust for gse cluster
               exp(coef) lower .95 upper .95 pvalue    se rob.se
PRKCI              1.382     0.600     3.181  0.447 0.400  0.426
age>50             0.911     0.342     2.424  0.851 0.495  0.499
nodepos            2.288     1.076     4.865  0.032 0.505  0.385
pcna117.n.scale    0.922     0.723     1.175  0.511 0.229  0.124
```

```
##################################### Adjust for gse cluster
                exp(coef) lower .95 upper .95 pvalue    se rob.se
PSMD2               2.960     1.231     7.120  0.015 0.659  0.448
age>50              0.796     0.273     2.323  0.676 0.498  0.546
nodepos             2.189     0.988     4.851  0.054 0.510  0.406
pcna117.n.scale     0.668     0.385     1.161  0.152 0.321  0.282


##################################### Adjust for gse cluster
                exp(coef) lower .95 upper .95 pvalue    se rob.se
SENP2               0.874     0.737     1.037  0.122 0.795  0.087
age>50              0.887     0.343     2.295  0.805 0.490  0.485
nodepos             2.312     0.975     5.481  0.057 0.508  0.440
pcna117.n.scale     0.987     0.760     1.282  0.922 0.227  0.133


##################################### Adjust for gse cluster
                exp(coef) lower .95 upper .95 pvalue    se rob.se
SENP5               3.407     1.044    11.123  0.042 0.792  0.604
age>50              0.814     0.293     2.256  0.692 0.496  0.520
nodepos             2.210     0.887     5.506  0.089 0.507  0.466
pcna117.n.scale     0.870     0.761     0.994  0.040 0.240  0.068


##################################### Adjust for gse cluster
                exp(coef) lower .95 upper .95 pvalue    se rob.se
UBXN7               1.986     1.239     3.185  0.004 0.379  0.241
age>50              0.830     0.266     2.585  0.747 0.498  0.580
nodepos             2.310     1.006     5.301  0.048 0.513  0.424
pcna117.n.scale     0.867     0.723     1.040  0.124 0.224  0.093


##################################### Adjust for gse cluster
                exp(coef) lower .95 upper .95 pvalue    se rob.se
ZNF639             92.073    26.766   316.716  0.000 1.942  0.630
age>50              0.935     0.387     2.260  0.882 0.490  0.450
nodepos             2.545     1.394     4.645  0.002 0.511  0.307
pcna117.n.scale     0.908     0.665     1.240  0.544 0.221  0.159
```

27.2 non-TNBC

```
for ( i in 1:length(selgene)) {
 get_analysis(datain=temp.TNBC0, outcome="DMFS.brain",xin=selgene[i], metflag="no")

}
```

```
 ################################### Adjust for gse cluster
                exp(coef) lower .95 upper .95 pvalue    se rob.se
ABCC5               1.127     0.975     1.303  0.105 0.440  0.074
age>50              2.112     1.456     3.063  0.000 0.651  0.190
nodepos             8.750     3.761    20.355  0.000 0.692  0.431
pcna117.n.scale     3.956     2.592     6.037  0.000 0.406  0.216


 ################################### Adjust for gse cluster
                exp(coef) lower .95 upper .95 pvalue    se rob.se
ACTL6A              1.764     1.499     2.075      0 0.507  0.083
age>50              2.119     1.429     3.142      0 0.649  0.201
nodepos             7.954     3.394    18.643      0 0.679  0.435
pcna117.n.scale     3.274     1.896     5.654      0 0.434  0.279


 ################################### Adjust for gse cluster
                exp(coef) lower .95 upper .95 pvalue    se rob.se
ATP11B              1.685     1.111     2.554  0.014 0.512  0.212
age>50              2.362     1.422     3.922  0.001 0.658  0.259
nodepos             8.260     3.628    18.807  0.000 0.689  0.420
pcna117.n.scale     3.941     2.525     6.152  0.000 0.408  0.227


 ################################### Adjust for gse cluster
                exp(coef) lower .95 upper .95 pvalue    se rob.se
DCUN1D1             1.553     0.218    11.056  0.660 0.823  1.002
age>50              2.070     1.367     3.136  0.001 0.647  0.212
nodepos             8.219     3.728    18.121  0.000 0.702  0.403
pcna117.n.scale     3.798     2.906     4.963  0.000 0.410  0.136


 ################################### Adjust for gse cluster
                exp(coef) lower .95 upper .95 pvalue    se rob.se
DLG1                1.697     0.140    20.495  0.677 0.804  1.271
```

```
age>50              2.134     1.340     3.397  0.001 0.646  0.237
nodepos             9.111     3.902    21.274  0.000 0.687  0.433
pcna117.n.scale     3.813     3.034     4.792  0.000 0.400  0.117


 ################################### Adjust for gse cluster
                exp(coef) lower .95 upper .95 pvalue    se rob.se
DVL3                0.877     0.351     2.190  0.779 0.714  0.467
age>50              2.157     1.280     3.634  0.004 0.662  0.266
nodepos             8.908     3.876    20.471  0.000 0.688  0.425
pcna117.n.scale     3.939     2.664     5.825  0.000 0.406  0.200


 ################################### Adjust for gse cluster
                exp(coef) lower .95 upper .95 pvalue    se rob.se
EIF2B5              1.618     0.722     3.629  0.243 0.794  0.412
age>50              2.038     1.346     3.084  0.001 0.653  0.211
nodepos             8.518     3.639    19.941  0.000 0.692  0.434
pcna117.n.scale     3.773     2.514     5.665  0.000 0.415  0.207


 ################################### Adjust for gse cluster
                exp(coef) lower .95 upper .95 pvalue    se rob.se
FXR1                1.326     0.488     3.605  0.580 0.643  0.510
age>50              2.169     1.292     3.641  0.003 0.653  0.264
nodepos             8.857     3.810    20.589  0.000 0.687  0.430
pcna117.n.scale     3.927     2.637     5.849  0.000 0.405  0.203


 ################################### Adjust for gse cluster
                exp(coef) lower .95 upper .95 pvalue    se rob.se
LSG1                2.781     1.648     4.695  0.000 0.751  0.267
age>50              1.964     1.282     3.007  0.002 0.650  0.217
nodepos             8.029     3.170    20.337  0.000 0.697  0.474
pcna117.n.scale     3.039     1.978     4.670  0.000 0.441  0.219


 ################################### Adjust for gse cluster
                exp(coef) lower .95 upper .95 pvalue    se rob.se
MFN1                1.492     0.371     5.999  0.573 0.604  0.710
age>50              2.043     1.493     2.794  0.000 0.649  0.160
nodepos             8.768     3.572    21.521  0.000 0.685  0.458
```

```
pcna117.n.scale     3.824     2.751     5.316  0.000 0.409  0.168


 ################################### Adjust for gse cluster
                exp(coef) lower .95 upper .95 pvalue    se rob.se
NCBP2               1.250     0.822     1.900  0.296 0.650  0.214
age>50              2.182     1.454     3.274  0.000 0.659  0.207
nodepos             8.919     3.936    20.206  0.000 0.689  0.417
pcna117.n.scale     3.880     2.406     6.258  0.000 0.410  0.244


 ################################### Adjust for gse cluster
                exp(coef) lower .95 upper .95 pvalue    se rob.se
NDUFB5              2.736     1.573     4.758  0.000 0.687  0.282
age>50              2.098     1.270     3.466  0.004 0.654  0.256
nodepos             8.798     3.621    21.380  0.000 0.683  0.453
pcna117.n.scale     3.574     2.378     5.372  0.000 0.414  0.208


 ################################### Adjust for gse cluster
                exp(coef) lower .95 upper .95 pvalue    se rob.se
PIK3CA              1.403     0.702     2.803  0.338 0.593  0.353
age>50              2.105     1.441     3.074  0.000 0.650  0.193
nodepos             8.577     3.698    19.896  0.000 0.682  0.429
pcna117.n.scale     3.967     2.489     6.323  0.000 0.404  0.238


 ################################### Adjust for gse cluster
                exp(coef) lower .95 upper .95 pvalue    se rob.se
PRKCI               1.955     0.775     4.931  0.155 0.600  0.472
age>50              1.862     1.112     3.116  0.018 0.649  0.263
nodepos             9.153     3.779    22.172  0.000 0.681  0.451
pcna117.n.scale     3.596     2.080     6.216  0.000 0.403  0.279


 ################################### Adjust for gse cluster
                exp(coef) lower .95 upper .95 pvalue    se rob.se
PSMD2               6.087     1.639    22.604  0.007 0.868  0.669
age>50              2.003     1.091     3.677  0.025 0.645  0.310
nodepos            10.717     4.802    23.919  0.000 0.710  0.410
pcna117.n.scale     2.436     1.166     5.089  0.018 0.479  0.376
```

```
#################################### Adjust for gse cluster
               exp(coef) lower .95 upper .95 pvalue    se rob.se
SENP2              4.687     1.302    16.876  0.018 0.882  0.654
age>50             2.524     1.273     5.004  0.008 0.670  0.349
nodepos            7.926     3.205    19.601  0.000 0.672  0.462
pcna117.n.scale    3.723     2.025     6.842  0.000 0.415  0.311


#################################### Adjust for gse cluster
               exp(coef) lower .95 upper .95 pvalue    se rob.se
SENP5              2.635     2.177     3.190  0.000 0.869  0.097
age>50             2.006     1.195     3.367  0.008 0.650  0.264
nodepos            7.838     3.399    18.073  0.000 0.697  0.426
pcna117.n.scale    3.402     2.255     5.134  0.000 0.423  0.210


#################################### Adjust for gse cluster
               exp(coef) lower .95 upper .95 pvalue    se rob.se
UBXN7              0.508     0.295     0.876  0.015 0.740  0.278
age>50             2.099     1.330     3.315  0.001 0.647  0.233
nodepos            9.596     3.773    24.403  0.000 0.705  0.476
pcna117.n.scale    4.193     2.696     6.520  0.000 0.417  0.225


#################################### Adjust for gse cluster
               exp(coef) lower .95 upper .95 pvalue    se rob.se
ZNF639             0.405     0.006    27.637  0.675 2.306  2.155
age>50             2.125     1.497     3.016  0.000 0.652  0.179
nodepos            8.539     3.274    22.269  0.000 0.694  0.489
pcna117.n.scale    4.061     2.868     5.751  0.000 0.414  0.177
```

28 Table S22

```
s20 <- data.frame("gene"=c("Basal", "Her2", "LumA", "LumB", "Normal"), "mut.pos"=c(9,21,175,56,5), "mut.neg"=c(122,45,230,129,17),
                  "amp.pos"=c(83,28,80,76,4), "amp.neg"=c(48,38,325,109,18))

s20$total <- s20[,2]+s20[,3]
s20$p=NA


for ( i in 1:nrow(s20)) {
p1= prop.test(as.numeric(c(s20[i,c(2,4)])), as.numeric(c(s20[i,6], s20[i,6])) )

# temp <- matrix(c(as.numeric(c(s20[i,c(2,4)])), as.numeric(c(s20[i,c(3,5)]))), ncol=2)
# f1 <- fisher.test(temp)
# ch1 <- chisq.test(temp)
s20$p[i] <- p1$p.value

}

s20$mut.per <- round(100*s20[,2]/(s20[,2]+s20[,3]),2)
s20$amp.per <- round(100*s20[,4]/(s20[,4]+s20[,5]),2)

xtable(s20[, c(1,8,9,6,7)])
```

	gene	mut.per	amp.per	total	p
1	Basal	6.87	63.36	131.00	0.00
2	Her2	31.82	42.42	66.00	0.28
3	LumA	43.21	19.75	405.00	0.00
4	LumB	30.27	41.08	185.00	0.04
5	Normal	22.73	18.18	22.00	1.00

```
####Supplemental material 4
####R functions used in the manuscript" A 3q gene signature associated with triple
negative breast cancer organ specific metastasis and
##response to neoadjuvant chemotherapy"

################### begin of function

get_KMplot <- function(datain=test.pcr, outcome="DMFS", group="pCR.vs.no") {



  if(outcome=="DMFS") {
    form1 <- paste( "Surv(t.dmfs, e.dmfs) ~ " , group, sep="")
    ylabin <- 'Overall DMFS Probability'
  }



  surv01 <- survfit(formula = as.formula(form1),
                    data = datain, na.action = na.exclude, conf.int = 0.95, se.fit =
T, type
                    = "kaplan-meier", error = "greenwood", conf.type = "log-log",
conf.lower
                    = "usual")

  #print(surv01)
  #print(summary(surv01, times=2))
  ptext <- " "

  if(group !="1") {

    survdif01 <- survdiff(formula = as.formula(form1),
                          data = datain, rho=0)

    tt <- table(datain[,group])

    surv.pvalue <- round(1-pchisq(survdif01$chisq, length(tt)-1) ,3)

    if(surv.pvalue < 0.001) ptext="P< 0.001"

    else ptext <- paste("p= ", surv.pvalue, sep="")

  }

  if(group !="FXR1") {
    # xaxisin=c(0:8)
    xaxisin=c(0:10)

    kmplot(surv01, mark=3, simple=FALSE, ylim=c(0,1), xlim=c(0,10),
           xaxis.at=xaxisin, xaxis.lab=xaxisin,# n.risk.at
           lty.surv=c(1,1,1), lwd.surv=5, col.surv=c(2,1,3), # survival.curves
           lty.ci=2,
           col.ci=0, # confidence intervals not plotted
           #group.names=c('No pCR','pCR'),
           group.order=c(2,3,1),
           extra.left.margin=6, label.n.at.risk=FALSE, draw.lines=TRUE,
           cex.axis=1.0, xlab='Years', ylab=ylabin, # labels
           lty.grid=1, lwd.grid=1, col.grid=grey(.9),
           legend=FALSE, loc.legend='bottomleft',  grid=FALSE,
           cex.lab=1.0, xaxs='r', bty='L', las=1, tcl=-.2

    )
```

```
  }  else
  {

    xaxisin=c(0:15)
    kmplot(surv01, mark=3, simple=FALSE, ylim=c(0,1), xlim=c(0,15),
           xaxis.at=xaxisin, xaxis.lab=xaxisin,# n.risk.at
           lty.surv=c(1,1,1), lwd.surv=2, col.surv=c(2,1,3), # survival.curves
           lty.ci=2,
           col.ci=0, # confidence intervals not plotted
           #group.names=c('No pCR','pCR'),
           #group.order=c(2,3,1),
           extra.left.margin=6, label.n.at.risk=FALSE, draw.lines=TRUE,
           cex.axis=1.0, xlab='Years', ylab=ylabin, # labels
           lty.grid=1, lwd.grid=1, col.grid=grey(.9),
           legend=FALSE, loc.legend='bottomleft',  grid=FALSE,
           cex.lab=1.0, xaxs='r', bty='L', las=1, tcl=-.2

    )


  }


  #abline(h=0.5, lty=2, col="gray")
  text(2, 0.2,  ptext, cex=1.0)




}




#####################################
get_lrm <- function(datain,  xin) {
  form1 <- paste("pCR.vs.no ~ ", xin, sep="")
  lrm1 <- lrm(as.formula(form1), data=datain, x=TRUE, y=TRUE)
  lrm1.rob <- robcov(lrm1, cluster=datain$dataset3)


  cat("\n ################################## Adjust for gse cluster   \n")
  lrm_OR(lrm1.rob)

}


lrm_OR <- function(lrmin) {

  temp <- data.frame("coef"=lrmin$coefficients)
  temp$se <- NA
  for ( i in 1:nrow(temp)) {
    temp$se[i] <- sqrt(lrmin$var[i,i])
  }

  temp$OR <- round(exp(temp$coef),3)
  temp$LCI <- round(exp(temp$coef-1.96*temp$se),3)
  temp$UCI <- round(exp(temp$coef+1.96*temp$se),3)
  temp$CI <- paste(temp$LCI,temp$UCI,sep=" to " )
  temp$p <- round(1-pchisq((temp$coef/temp$se)^2,1) ,4)
  temp$coef <- round(temp$coef ,3)
  temp$se <- round(temp$se,3)

  colnames(temp) <- c("Beta", "se", "OR", "LCI", "UCI", "CI","p-value")
```

```
  print(temp[-1,c(1:3,6,7)])

}



#######################
get_analysis <- function(datain, outcome, xin, metflag) {

  if(outcome=="DMFS") form1 <- "Surv(t.dmfs, e.dmfs) ~ "
  if(outcome=="RFS") form1 <- "Surv(t.rfs, e.rfs) ~ "
  if(outcome=="OS") form1 <- "Surv(t.os, e.os) ~ "
  if(outcome=="DSS") form1 <- "Surv(t.os, e.dss) ~ "
  if(outcome=="DMFS.lung") form1 <- "Surv(t.dmfs, e.met.lung) ~ "
  if(outcome=="DMFS.brain") form1 <- "Surv(t.dmfs, e.met.brain) ~ "
  if(outcome=="DMFS.bone") form1 <- "Surv(t.dmfs, e.met.bone) ~ "

  form1 <- paste(form1, xin, sep="")

  a3 <- names(table(datain$dataset3))

  # if(length(a3) > 2) {
  if(metflag=="yes") {
    cname <- c("beta", "se", "p", "gse", "n.all", "n.event")
    result <- data.frame(matrix(rep(NA, length=length(cname)*length(a3)),
ncol=length(cname)))
    colnames(result) <- cname

    for ( i in 1:length(a3)) {
      # for ( i in 24:26 ) {
      #  print(i)
      temp <- subset(datain, dataset3 %in% a3[i])
      #temp[,Cs(t.dmfs, e.dmfs, ch3q20.19.n.scale, dataset3)]

      #cph1 <- cph(Surv(t.dmfs, e.dmfs) ~ ch3q20.19.n.scale, data=temp)
      cox1 <- try(coxph(as.formula(form1), data=temp), silent=TRUE)
      if(class(cox1) !="try-error") {
        aa <- summary(cox1)$coefficients
        result[i,c(1:3)] <- aa[c(1,3,5)]
      }

      result$n.event[i] <- sum(temp$e.dmfs, na.rm=TRUE)
      result$n.all[i] <- sum(table(temp$e.dmfs))
      result$gse[i] <- a3[i]
    }

    GSEresult <- subset(result, !is.na(p))

    met.result <- metagen(GSEresult$beta, GSEresult$se, sm="HR",
studlab=GSEresult$gse)
    cat("\n ############################## Meta analysis \n")
    print(met.result)
    forest(met.result, leftcols="studlab")
    #forest(met.result)

  }

  if(length(a3) > 1 ) {
    ####### adjust for cluster
    cat("\n \n ################################### Adjust for gse cluster   \n")
    cox2 <- try(coxph(as.formula(paste(form1,"+cluster(dataset3)", sep="")),
data=datain), silent=TRUE)
```

```
    cph2 <- cph(as.formula(form1), data=datain, x=TRUE, y=TRUE)
    cph2.rob <- robcov(cph2, cluster=datain$dataset3)

    if(class(cox2) != "try-error") {
      aa<-summary(cox2)
      #print(round(aa$coefficients,4))
      #print(round(aa$conf.int,4))
      aa1 <- aa$coefficients
      aa2 <- aa$conf.int
      aa3 <- cbind(aa2,"pvalue"=aa1[,ncol(aa1)], "se"=aa1[,3], "rob.se"=aa1[,4])

      if(nrow(aa3) > 1) print(round(aa3[,-c(2)],3)) else
        print(round(aa3,3))
    }  else
      print( "ERROR !!!!!!!")
  }  else

  {



    cat("\n   #################################### Not Adjust for gse cluster ( one
gse)   \n")
    cox2.1 <- try(coxph(as.formula(form1), data=datain), silent=TRUE)
    if(class(cox2.1) != "try-error") {
      aa<-summary(cox2.1)
      #print(round(aa$coefficients,4))
      #print(round(aa$conf.int,4))
      aa1 <- aa$coefficients
      aa2 <- aa$conf.int
      aa3 <- cbind(aa2,"pvalue"=aa1[,ncol(aa1)])
      if(nrow(aa3) > 1) print(round(aa3[,-c(2)],3)) else
        print(round(aa3,3))
    } else
      print( "ERROR !!!!!!!")

  }




}


#####################
kmplot <- function(km, mark=3, simple=FALSE,
                   xaxis.at=pretty(km$time), xaxis.lab=xaxis.at,
                   yaxis.at=pretty(c(0,1))  ,     ##### HC added
                   lty.surv=1, lwd.surv=1, col.surv=1,
                   lty.ci=0, lwd.ci=1, col.ci=col.surv, #By default (lty.ci=0),
confidence intervals are not plotted.
                   group.names=NULL, group.order=seq(length(km$n)),
extra.left.margin=4,
                   label.n.at.risk=FALSE, draw.lines=TRUE, cex.axis=1,
                   xlab='', ylab='', main='', xlim=c(0,max(km$time)), ylim=c(0,1),
                   grid=TRUE, lty.grid=1, lwd.grid=1, col.grid=grey(.9),
                   legend=!is.null(km$strata), loc.legend='topright', add=FALSE,
                   firstyin=1,
                   ... # ... is passed to par()
){
```

```
  ng0 <- length( km$strata ) ; ng <- max(ng0,1)
  # When only one group...
  if(ng0==0){ km$strata <- length(km$time) ; names(km$strata) <- 'All' ; legend <-
draw.lines <- FALSE }

  lty.surv <- rep(lty.surv, ng) ; lwd.surv <- rep(lwd.surv, ng) ; col.surv <-
rep(col.surv, ng)
  lty.ci <- rep(lty.ci, ng) ;     lwd.ci <- rep(lwd.ci, ng)     ; col.ci <-
rep(col.ci, ng)

  ## group names and error checking
  gr <- c(km$strata)
  if( is.null(group.names) ){ group.names <- names(km$strata) }
  if( length(unique(group.names)) != ng ){ stop('\n','length(unique(group.names)) !=
number of groups.') }
  if( suppressWarnings(any( sort(group.order) != 1:ng)) )
  { stop('\n', 'Something wrong with group.order.','\n','sort(group.order) must
equal 1:', ng, '.') }
  group.names <- gsub(' *$', '', group.names)  #to remove unwanted white spaces in
group.names.
  if(ng==1 & (group.names[1]=='group.names') ){ group.names <- 'N at risk' ;
label.n.at.risk = FALSE }

  ## graphic parameters
  if(!add){
    par(list(oma=c(1,1,1,1), mar=c(4+ng,4+extra.left.margin,4,2)+.1))
    if(simple) par( mar=c(3,4,2,1)+.1 )
    par( list(...) )
  }

  ## reformat survival estimates
  dat <- data.frame(time=km$time, n.risk=km$n.risk, n.event=km$n.event,
survival=km$surv, std.err=km$std.err,
                    lower=km$lower, upper=km$upper, group=rep( group.names, gr) )
  dat.list <- split(dat, f=dat$group)

  ## plot (but not survival curves)
  par(xaxs="i")  ### Shilin added to make
  par(yaxs="i")  ### Shilin added to make y begin at 0, end at 1
  plot(0,type='n', xlim=xlim, ylim=ylim, xaxt='n', yaxt='n', xlab='', ylab='' )
  if(grid){
    par('xpd'=FALSE)
    abline(v=xaxis.at,       lty=lty.grid, lwd=lwd.grid, col=col.grid )
    abline(h=pretty(c(0,1)), lty=lty.grid, lwd=lwd.grid, col=col.grid )
  }
  #     axis( side=2, at=pretty(c(0,1)), cex.axis=cex.axis )
  axis( side=2, at=yaxis.at, cex.axis=cex.axis )
  axis( side=1, at=xaxis.at, label=xaxis.lab, line=-0.5, tick=FALSE,
cex.axis=cex.axis )
  axis( side=1, at=xaxis.at, label=rep('',length(xaxis.at)), line=0, tick=TRUE )
  title(xlab=xlab, line=1.5, adj=.5, ...) ; title(ylab=ylab, ... )

  if(!simple){
    ## write group names
    group.name.pos <- (par()$usr[2]-par()$usr[1]) / -8 ; padding <- abs(
group.name.pos / 8 )
    line.pos <- (1:ng)[order(group.order)] + 2
    mtext( group.names, side=1, line=line.pos, at=group.name.pos, adj=1, col=1,
las=1, cex=cex.axis )

    ## draw matching lines for n at risk.
    if(draw.lines){
      par('xpd'=TRUE)
```

```
      for(i in 1:ng){
        axis(side=1, at=c(group.name.pos+padding,0-2*padding), labels=FALSE,
line=line.pos[i]+0.6, lwd.ticks=0,
             col=col.surv[i], lty=lty.surv[i], lwd=lwd.surv[i] ) }
    }

    ## numbers at risk
    kms <- summary(km, times=xaxis.at) ; if(is.null(kms$strata)) kms$strata <-
rep(1,length(kms$time) )
    d1 <- data.frame(time = kms$time, n.risk = kms$n.risk, strata = c(kms$strata))
    d2 <- split(d1, f=d1$strata)

    ## Right-justifying the numbers
    ndigits <- lapply(d2, function(x) nchar(x[,2]) )
    max.len <- max( sapply(ndigits, length) )
    L <- do.call('rbind', lapply(ndigits, function(z){ length(z) <- max.len ; z} ) )
    nd <- apply( L, 2, max, na.rm=T )
    for( i in seq(ng) ){
      this <- d2[[i]]
      w.adj <- strwidth('0', cex=cex.axis, font=par('font')) / 2 * nd[1:nrow(this)]
      mtext( side=1, at=this$time+w.adj, text=this$n.risk, line=line.pos[i],
cex=cex.axis, adj=1, col=1, las=1)
    }
    if(label.n.at.risk) mtext( side=1, text='N at risk', at=group.name.pos,
line=1.5, adj=1, col=1, las=1, cex=cex.axis )
  } ## End of if(!simple)

  # Legend
  rlp <- group.order
  if(legend){
    bgc <- ifelse( par('bg')=='transparent', 'white', par('bg') )
    legend(x=loc.legend, legend=group.names[rlp], col=col.surv[rlp],
lty=lty.surv[rlp], lwd=lwd.surv[rlp],
           bty='o', cex=cex.axis, bg=bgc, box.col='transparent', inset=.01 )
  }


  lines(km, conf.int=FALSE, col=col.surv, lty=lty.surv, lwd=lwd.surv, mark=mark,
xpd=FALSE, firsty=firstyin ) ### Heidi add firstyin

}
```
